# NMR lineshape analysis using analytical solutions of multi-state chemical exchange with applications to kinetics of host–guest systems

**DOI:** 10.1038/s41598-022-20136-4

**Published:** 2022-10-17

**Authors:** Václav Březina, Lenka Hanyková, Nadiia Velychkivska, Jonathan P. Hill, Jan Labuta

**Affiliations:** 1 International Center for Materials Nanoarchitectonics (WPI-MANA), National Institute for Materials Science (NIMS), 1-1 Namiki, Tsukuba, Ibaraki 305-0044 Japan; 2 Faculty of Mathematics and Physics, Charles University, V Holešovičkách 2, 180 00 Prague 8, Czech Republic; 3 Institute of Macromolecular Chemistry, Academy of Sciences of the Czech Republic, Heyrovsky Sq. 2, 162 06 Prague 6, Czech Republic

**Keywords:** Supramolecular chemistry, Physical chemistry, Chemical physics, Reaction kinetics and dynamics, Solution-state NMR

## Abstract

Nuclear magnetic resonance (NMR) lineshape analysis is a powerful tool for the study of chemical kinetics. Here we provide techniques for analysis of the relationship between experimentally observed spin kinetics (transitions between different environments $A,B,\dots$) and corresponding chemical kinetics (transitions between distinct chemical species; e.g., free host and complexed host molecule). The advantages of using analytical solutions for two-, three- or generally *N*-state exchange lineshapes (without J-coupling) over the widely used numerical calculation for NMR spectral fitting are presented. Several aspects of exchange kinetics including the generalization of coalescence conditions in two-state exchange, the possibility of multiple processes between two states, and differences between equilibrium and steady-state modes are discussed. ‘Reduced equivalent schemes’ are introduced for spin kinetics containing fast-exchanging states, effectively reducing the number of exchanging states. The theoretical results have been used to analyze a host–guest system containing an oxoporphyrinogen complexed with camphorsulfonic acid and several other literature examples, including isomerization, protein kinetics, or enzymatic reactions. The theoretical treatment and experimental examples present an expansion of the systematic approach to rigorous analyses of systems with rich chemical kinetics through NMR lineshape analysis.

## Introduction

**Figure 1:**
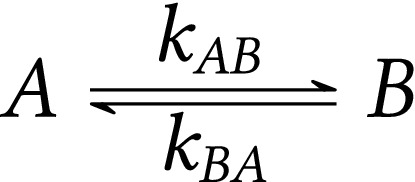
Spin kinetics of two-state exchange.

In NMR terminology, a process by which a particular nuclear spin changes its position among various chemical environments (i.e., states $A,B,\dots$) is called chemical exchange. We denote these transitions between states as spin kinetics. The chemical exchange is characterized by transition rate coefficients $k_{ij}$ (in units of s$^{-1}$), where $i,j=A,B,\dots$; $i \ne j$. These coefficients are often denoted as ‘rate constants’, although we apply the term ‘transition rate coefficients’ due to their possible dependence on concentration. This concentration dependence may occur when spin kinetics is compared with the corresponding chemical kinetics (characterized by concentration-independent reaction rate coefficients). An example of this is a bimolecular reaction discussed in detail in "Chemical exchange in host–guest complexes" Section. The simplest and most common case of a reversible process is a two-state chemical exchange between *A* and *B* spin states (i.e., between states with different chemical environments and thus different Larmor frequencies) described by two transition rate coefficients $k_{AB}$, $k_{BA}$ as illustrated in Fig. 1.

NMR lineshape fitting is a straightforward method to obtain transition rate coefficients and often requires only the acquisition of simple 1D NMR spectra^1–3^. For more complicated molecules (e.g., proteins) HSQC spectra can be analyzed, where either a 1D crosssection^4–6^ or the full 2D spectral^7–9^ lineshape is fitted. NMR lineshape fitting procedure is most suitable for the analysis of exchange processes with transition rate coefficients approximately in the range of 10–$10^5$ s$^{-1}$ for $^1$H NMR spectra^7,10^. The validity of this range based on the NMR instrument capability and the spectra lineshape fitting limitations (accuracy) are discussed in detail in Section S7.4 in Supplementary Information (SI).

Methods other than lineshape fitting exist for the determination of transition rate coefficients. However, those methods usually require elaborate NMR sequences and extended experimental time. They are also aimed at the analysis of different ranges of transition rate coefficients (both higher and lower). Reviews of these methods (e.g., ZZ-exchange, EXSY, $R_2$ relaxation dispersion) are available from Bain^11^, Kleckner^12^ or Furukawa^13^.

The present study consists of theoretical ("Two-state exchange", "Three-state exchange", "Exchange schemes containing a fast process", and "Chemical exchange in host–guest complexes" Sections) and experimental parts ("Analysis of an actual system exhibiting multi-state exchange" Section). In the theoretical part, analytical solutions for two- and three-state exchange spectral lineshapes in the absence of J-coupling (with possible generalization to *N* states) are discussed, including their applications to lineshape fitting, calculation of coalescence points and construction of reduced equivalent schemes in the case of fast exchange regime. The presence of multiple processes between two states or the possibility of steady-state mode in some systems is also considered. In the experimental part ("Analysis of an actual system exhibiting multi-state exchange" Section), an analysis is presented of an actual system involving a di-bromobenzylated oxoporphyrinogen host complexed with a (*R*)-camphorsulfonic acid guest by means of lineshape analysis of $^1\hbox {H}$ NMR spectra. These spectra were obtained by titration experiments during our previous work^14^, where binding and halochromic properties of the host were studied in the presence of organic acid guests. We propose a six-state spin kinetics scheme for the central NH resonances of the host molecule and determine the underlying chemical kinetics scheme. The parameters of both the chemical kinetics and spin kinetics schemes and the concentration dependence of the transition rate coefficients are then determined in their entirety by the lineshape fitting procedure in conjunction with inferences from the theoretical part.

Let us emphasize the main benefits of exact analytical solutions of NMR lineshapes over the possible numerical solutions. Analytical solutions enable complex analyses of coalescence conditions in symmetric and (general) asymmetric two-state exchange (Section S5 in SI). These results can be readily applied for a more accurate estimate of the transition rate coefficients at coalescence $k_\text {c}$ (e.g., Eq. (8)). Exact solutions provide interpretation of exchange lineshapes in slow or fast exchange limits (Section S4 in SI) and also, in this regard, a means for the construction of reduced equivalent schemes in the presence of a fast exchange process ("Exchange schemes containing a fast process" Section). Moreover, analytical solutions are facile to implement and perform faster in lineshape fitting code than numerical ones since they do not require the calculation of an inverse matrix for each point of the spectrum (Sections S1.1 and S3 in SI).

## Two-state exchange

### Analytical solution for the spectral lineshape

The NMR signal is proportional to the complex transverse magnetization (equivalent to $-1$ quantum coherence in quantum mechanical description^15^) obtained it the form $M_{xy}^{j}=M_x^{j}+iM_y^{j}$, where $j=A,B,\dots$ are spin states and *i* is the imaginary unit. Under chemical exchange of non-J-coupled spins, the evolution of transverse magnetization $M_{xy}^{j}$ can be modeled classically using Bloch-McConnel equations^16,17^. Its free evolution after a pulse is described as follows

1 $$\begin{aligned} \frac{\mathrm{d}}{\mathrm{d} t} {\mathbf {M}}_{xy}(t) =\left( i \mathbf {L}-\mathbf {R_2}+\mathbf {K}\right) \mathbf {M}_{xy}(t), \end{aligned}$$

where $\mathbf {M}_{xy}=(M_{xy}^{A},M_{xy}^{B})^\text {T}$, the diagonal matrix **L** describes evolution due to the external magnetic field, the diagonal matrix $\mathbf {R_2}$ accounts for spin-spin relaxation, and the kinetic matrix **K** characterizes flux of magnetization from one state to another due to the chemical exchange. Equation (1) can be solved numerically in the frequency domain, see Sections S1 and S3 in SI. However, this study aims to identify analytical solutions, which yield deeper insights into the general lineshape analysis.

In the two-state case, $\mathbf {L}=\text {diag}(\omega _A,\omega _B)$, $\mathbf {R_2}=\text {diag}(R_2^A,R_2^B)$ (‘diag($\bullet$)’ represents a diagonal matrix with elements indicated in brackets), where $R_2^A$, $R_2^B$ are transverse relaxation rates for *A* and *B* states, respectively, and the kinetic matrix is expressed as

2 $$\begin{aligned} \mathbf {K}= \begin{pmatrix} -k_{AB} &{} k_{BA} \\ k_{AB} &{} -k_{BA} \end{pmatrix} \,. \end{aligned}$$

Concentrations of spins *A* and *B*, denoted as [*A*] and [*B*], respectively, can be written in the form of relative populations $p_A$ and $p_B$ (with normalization $p_A + p_B = 1$)

3a $$\begin{aligned} p_A&=\frac{[A]}{[A]+[B]} \,, \end{aligned}$$

3b $$\begin{aligned} p_B&=\frac{[B]}{[A]+[B]} \,. \end{aligned}$$

Time dependence of populations is governed by two first-order kinetics differential equations with the following matrix form

4 $$\begin{aligned} \frac{\mathrm{d} \mathbf {p}}{\mathrm{d} t} = \mathbf {K} \mathbf {p}\,, \end{aligned}$$

where $\mathbf {p}=(p_A,p_B)^\text {T}$. We are interested in the equilibrium state, which sets the time derivative at the left-hand side of Eq. (4) to zero and converts this differential equation into an algebraic equation

5 $$\begin{aligned} \mathbf {K} \, \mathbf {p}=\mathbf {0} \, . \end{aligned}$$

Formally, Eqs. (4) and (5) hold for any number of states *N* with populations $\mathbf {p}=(p_A,p_B,\dots ,p_N)^\text {T}$ when the kinetic matrix **K** is correspondingly constructed. The solution for equilibrium (Eqs. (5)) can be readily obtained by a diagrammatic method introduced in King and Altman^18^ or Hill^19^. Apart from equilibrium, this solution also describes the steady-state, see discussion in the next section.

The equilibrium populations in the two-state exchange (Fig. 1) can be expressed as functions of the transition rate coefficients (from Eqs. (2) and (5))

6a $$\begin{aligned} p_A^\text {eq}&= \frac{k_{BA}}{k_{AB}+k_{BA}} \,, \end{aligned}$$

6b $$\begin{aligned} p_B^\text {eq}&= \frac{k_{AB}}{k_{AB}+k_{BA}} \, . \end{aligned}$$

Without loss of generality, let us assume that the initial transverse magnetization is real and positive, $M_{xy}^{j}(t=0)=M^j_0$, $M^j_0 \in \mathbb {R}$, $j=A,B$. This corresponds to the magnetization vectors (of nuclei of interest in the states *A* and *B*) being tilted parallel to the *x*-axis, as can be seen from the definition $M_{xy}^{j}=M_x^{j}+iM_y^{j}$ (the imaginary *y* component is set to zero).

A solution of the system of linear differential equations in Eq. (1) for symmetric two-state exchange where $k_{AB}=k_{BA}=k$ is a standard part of literature reports^11,15,17^. Also, an analytical solution in complex form for the asymmetric case has already been published by Gutowski and Saika^20^, Johnson^21^ and Římal^1^. The formula in complex form reads

7a $$\begin{aligned} S_\text {two-state exch.}(\omega )= M_0\frac{p_A \alpha _B + p_B \alpha _A + k_{AB} + k_{BA}}{\alpha _A \alpha _B + k_{AB} \alpha _B + k_{BA} \alpha _A}\,, \end{aligned}$$

where

7b $$\begin{aligned} \alpha _{j}= R_2^{j} + i (\omega -\omega _{j}) \end{aligned}$$

for $j=A,B$. $M_0=M_0^A+M_0^B$ denotes the total transverse magnetization. The sign of the imaginary part of $\alpha _j$ depends on the convention in Fourier transformation (FT)^22^. The expression $\alpha _j^{-1}$ describes the complex Lorentzian lineshape with Larmor frequency $\omega _j$ and transverse relaxation $R_2^j$. This is useful for calculating limit cases when the transition rate coefficients tend to zero or infinity, see Section S4 in SI. At equilibrium, the populations and transition rate coefficients in Eq. (7a) are connected by the algebraic Eqs. (6a,b).

For lineshape fitting, it is convenient to express the real (absorption) part of the spectrum from Eq. (7). The result can be found in the report by Takai et al.^3^ and, for convenience, we show it here for the symmetric and asymmetric cases in SI (Eqs. S8–S10 in Section S2, with corresponding *MATLAB* code in Section S3).

Using the analytical spectral lineshape, we also calculated the position of coalescence point $k_\text {c}$ in the case of symmetric two-state exchange (i.e., $k_{AB}=k_{BA}=k$), see Section S5.1 in SI. If the relaxation rate is neglected ($R_2 = 0$), then the approximative formula is the well-known $k_\text {c} \!\approx \! |\omega _B - \omega _A|/(2\sqrt{2})$^15^. We have derived corrections to this approximation in the form of a power series in $R_2/\Delta \omega _{AB}$ terms ($\Delta \omega _{AB}=|\omega _B - \omega _A|$). Equation (8) is the formula for $k_\text {c}$ containing a linear correction term,

8 $$\begin{aligned} k_\text {c} \!\approx \! \frac{\Delta \omega _{AB}}{2\sqrt{2}} \left[ 1-1.1379\frac{R_2}{\Delta \omega _{AB}} \right] \, . \end{aligned}$$

The correction term provides significant error reduction (see Fig. S1 for error analyses) and Eq. (8) can be readily applied in the experiment for more precise estimation of $k_\text {c}$. Furthermore, we have generalized the concept of coalescence and investigated the coalescence conditions for asymmetric two-state exchange, see Section S5.2 in SI. In contrast to symmetric two-state exchange, where the coalescence transition rate coefficient is a single value $k_\text {c}$, asymmetric exchange exhibits infinitely many pairs of transition rate coefficients {$k_{AB,\text {c}}$, $k_{BA,\text {c}}$} at which the spectrum has coalescence lineshapes (provided constant values of $\omega _A$, $\omega _B$, and $R_2$).

### Two-state exchange at equilibrium and at steady-state

It has already been mentioned above that Eq. (5) also allows a steady-state solution. To understand the difference between equilibrium and steady-state, let us define a *population flux* from state *j* to state *k* as

9 $$\begin{aligned} J_{jk}=p_j k_{jk}-p_k k_{kj} \,. \end{aligned}$$

It follows then from Eq. (9) that $J_{jk}=-J_{kj}$.

A condition for* equilibrium *is that population flux between each state *j* and *k* is equal to zero ($J_{jk}=0$). In contrast, in *steady-state* mode non-zero population fluxes (constant in time) are present, but the net flux ${\mathscr{J}}_k$ to any state *k* is zero (see Eq. (10)), so that the populations do not change over time,

10 $$\begin{aligned} {\mathscr{J}}_k= \!\!\!\!\!\! \sum _{\begin{array}{c} j=A,B,\dots \\ j\ne k \end{array}} \!\!\!\!\!\! J_{jk} = 0 \end{aligned}$$

for all $k=A,B,\dots$ . Note that $$\sum _{\begin{array}{c} k=A,B,\dots \end{array}} {\mathscr{J}}_k = 0$$ even outside equilibrium or steady-state because the spin in states $A,B,\dots$ is an isolated system.

The condition of zero population flux for equilibrium can be reformulated using populations and transition rate coefficients setting $J_{jk}=0$ in Eq. (9). Hence, at equilibrium (but not at steady-state)

11 $$\begin{aligned} \frac{p_k^\text {eq}}{p_j^\text {eq}} = \frac{k_{jk}}{k_{kj}} \,. \end{aligned}$$

As follows from these relationships, the equilibrium conditions reduce the number of independent transition rate coefficients compared to any out-of-equilibrium state. This must be considered during the analysis of actual experimental data.

A steady-state spin kinetics can be achieved only in schemes containing a closed cycle^19^. Closed cycle exists in the scheme if it is possible to return to the same state through a different process (i.e., through a different transition state). Since closed cycles are not contained in the simple two-state exchange, as presented in Fig. 1, a steady-state mode is not feasible. It also follows from Eqs. (2) and (5) that $J_{AB}=J_{BA}=0$, which is equivalent to the equilibrium condition in Eq. (11). However, even in two-state kinetics, a closed cycle can arise if the transition between states is accomplished by more than one process.

**Figure 2:**
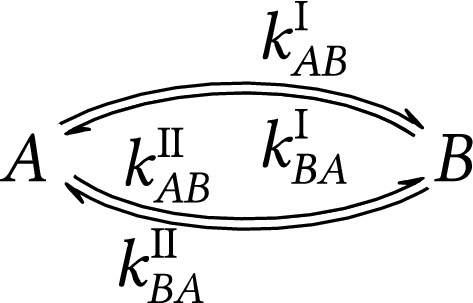
Spin kinetics of two-state exchange where two independent processes are present.

For an illustration of two-state kinetics with two reversible processes, see Fig. 2. In that case, there exist two independent reversible processes I ($k_{AB}^\text {I}$ and $k_{BA}^\text {I}$) and II ($k_{AB}^\text {II}$ and $k_{BA}^\text {II}$) both of which cause interconversion of *A* and *B* states. Since a closed cycle is present, in theory, there can be non-zero population flux in clockwise ($J_{AB}^\text {I}=J_{BA}^\text {II}>0$), or counterclockwise ($J_{AB}^\text {I}=J_{BA}^\text {II}<0$) directions. However, the NMR spectral lineshape for a two-process two-state exchange (Fig. 2) is in principle indistinguishable from one-process two-state exchange (Fig. 1), see SI Section S6.1. Consequently, steady-state and equilibrium modes in the two-process case also cannot be distinguished (both can be mapped on an equilibrium one-process lineshape).

An example of two-process two-state kinetics is presented by the conversion of a substrate to a product in reversible enzyme-catalyzed reactions according to the Michaelis-Menten scheme. After an initial pre-steady-state period (usually very brief) the chemical reaction achieves an approximate steady-state, where free and bound enzyme concentrations change slowly (this is a standard assumption in the analysis of enzyme kinetics^23^). Consequently, a nuclear spin located at the enzyme undergoes exchange between free and bound states in an approximate steady-state mode. This approximate steady-state eventually reaches equilibrium unless forced by an external action (e.g., by addition of substrate and removal of product). However, even in the absence of an enforcing factor, the enzyme spin populations achieve an approximate steady-state over a relatively long time period. For further details see Section S6.2 in SI.

Another example of two-process two-state spin kinetics is conformational variation in dimesityl systems, where both mesityl rings can flip about the connecting single bonds^24–26^. These ring flips interconvert *P* and *M* helical enantiomers. There are several different transition state geometries, distinguishing different types of processes. Each process converts the enantiomer although the spin state can remain unchanged. For more details see Section S6.3 in SI. There are also examples of systems having two states and undergoing more than two processes including correlated motions of trimesityl compounds^27^ or other propeller-like triaryl systems^28^.

## Three-state exchange

### Analytical solution for spectral lineshape

Using FT, spectral exchange lineshapes can be obtained analytically for systems having arbitrary number of spin states. Details of the solutions and numerical implementations in *MATLAB* can be found in SI (Sections S1 and S3). In this paper, we focus on detailed analyses of three-state chemical exchange. To the best of our knowledge, the general analytical solution presented here has not been reported. There have been several attempts during the early years of NMR spectroscopy to obtain exact solutions in the simplified cases presented by Gutowsky and Saika^20^ or Sack^29^. It should be noted that Kovrigin^30^ analyzed numerically several properties of three-state (and four-state) chemical exchange. There also exist NMR spectral simulation programs (e.g., *NmrLineGuru*^31^, *TITAN*^7^), which enable the numerical fitting of multi-state exchange lineshapes. As mentioned above, our aim is to identify an exact analytical solution that yields deeper insight into the NMR spectral manifestation of the three-state exchange.

**Figure 3:**
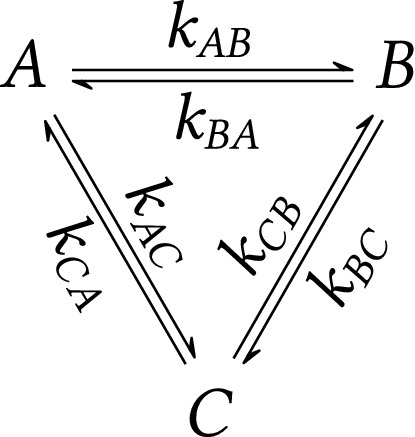
Spin kinetics of three-state exchange.

Transitions among three states are described by six transition rate coefficients according to Fig. 3 with the corresponding kinetic matrix

12 $$\begin{aligned} \mathbf {K}= \begin{pmatrix} -k_{AB}-k_{AC} &{} k_{BA} &{} k_{CA} \\ k_{AB} &{} -k_{BA}-k_{BC} &{} k_{CB} \\ k_{AC} &{} k_{BC} &{} -k_{CA}-k_{CB} \end{pmatrix}\,. \end{aligned}$$

The equilibrium/steady-state populations can be determined using the equation $\mathbf {K}\mathbf {p}=\mathbf {0}$ (Eq. (5)) with populations $\mathbf {p}=(p_A,p_B,p_C)^\text {T}$. Details of the solution (Eq. (15) in Table 1) will be discussed below. The spectral lineshape for three-state chemical exchange is as follows

13 $$\begin{aligned} S_\text {three-state exch.}(\omega )=M_0\frac{\mathscr{P}}{\mathscr {Q}} \,, \end{aligned}$$

where

$$\begin{aligned} M_0 =&\, M_0^A+M_0^B+M_0^C \,,&\\ \mathscr{P} =&\,p_A [ \alpha _B \alpha _C + \alpha _B (k_{CA}+k_{CB} + k_{AC}) + \alpha _C (k_{BA}+k_{BC} + k_{AB})]&\\&+ p_B [\alpha _A \alpha _C +\alpha _A (k_{CA}+k_{CB}+k_{BC}) + \alpha _C (k_{AB}+k_{AC} + k_{BA})]&\\&+ p_C [\alpha _A \alpha _B + \alpha _A (k_{BA}+k_{BC} + k_{CB}) + \alpha _B (k_{AB}+k_{AC} + k_{CA})]&\\&+ \pi _A + \pi _B + \pi _C \,, \\ \mathscr{Q} =&\, \alpha _A \alpha _B \alpha _C + \alpha _A \alpha _B (k_{CA}+k_{CB}) + \alpha _A \alpha _C (k_{BA}+k_{BC}) + \alpha _B \alpha _C (k_{AB}+k_{AC})&\\&+ \alpha _A \pi _A + \alpha _B \pi _B + \alpha _C \pi _C \,,&\\ \pi _A =&\, k_{BA}k_{CA}+k_{BC}k_{CA}+k_{BA}k_{CB}\,,&\\ \pi _B =&\,k_{AB}k_{CA}+k_{AB}k_{CB}+k_{AC}k_{CB}\,,&\\ \pi _C =&\, k_{AC}k_{BA}+k_{AB}k_{BC}+k_{AC}k_{BC}\,.&\end{aligned}$$

### Kinetics of three-state exchange

In contrast to simple two-state kinetics (Fig. 1), general three-state kinetics (Fig. 3) contains a cycle thus enabling the existence of a steady-state. The expressions for steady-state and equilibrium populations of the general three-state kinetics and two special cases are summarized in Table 1. For special cases, it can be seen that imposing symmetry (Table 1b) or complexity reduction (Table 1c) to the general three-state scheme leads only to solutions in equilibrium and a steady-state solution is absent (for more details, see text below).

In the case of general three-state kinetics (Table 1a), the populations for steady-state $p_j^\text {ss}$ are calculated from Eqs. (5) and (12), the solution is given in Eq. (15) in Table 1, and the coefficients $\pi _{j}$ are defined in Eq. (13).

At equilibrium, the solution (Eq. (15) in Table 1) further simplifies when it is combined with the equilibrium conditions (Eq. (11)). Since now the transition rate coefficients are not mutually independent,

14 $$\begin{aligned} 1 = \frac{p_C^\text {eq}}{p_B^\text {eq}}\frac{p_B^\text {eq}}{p_A^\text {eq}}\frac{p_A^\text {eq}}{p_C^\text {eq}} = \frac{k_{BC}}{k_{CB}}\frac{k_{AB}}{k_{BA}}\frac{k_{CA}}{k_{AC}} \,. \end{aligned}$$

**Table 1:**
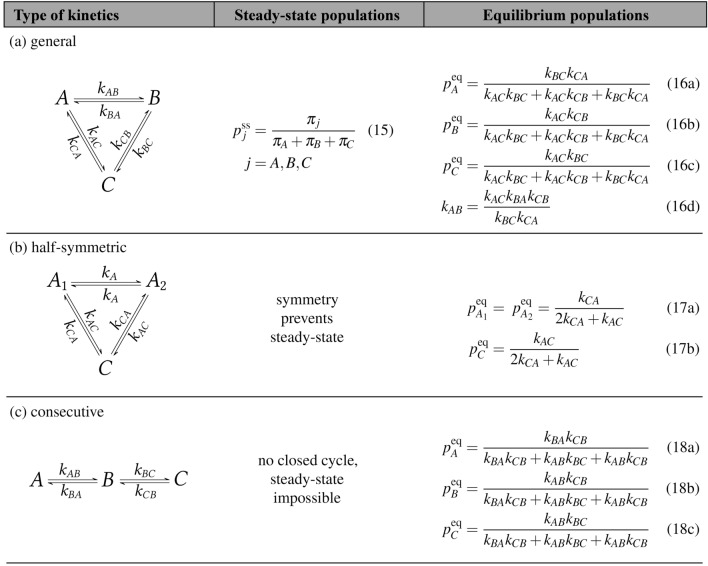
Three-state spin kinetics and its special cases.

If we choose, for example, $k_{BA}$ as the dependent rate coefficient, then the equilibrium populations can be obtained according to Eqs. (16a-c) in Table 1. The dependent transition rate coefficient $k_{BA}$ is calculated according to Eq. (16d). Without loss of generality, it is possible to select any other transition rate coefficient as dependent.

It follows from these relationships that the equilibrium condition reduces the number of independent transition rate coefficients, and this fact must be taken into consideration during analysis of any actual experimental data. Prior to use of the three-state lineshape formula in Eq. (13), the populations and transition rate coefficients for the steady-state should be related using Eq. (15) in Table 1 and for equilibrium using Eq. (16) in Table 1.

Details about lineshape fitting in different modes (i.e., equilibrium, steady-state and out-of-steady-state mode with time-dependent populations) of the spin kinetics and discussion about the interdependency of the fitted parameters during lineshape analysis can be found in SI, Sections S7.1 and S7.2. It is also worth mentioning that a steady-state in the general three-state exchange scheme cannot be distinguished from an equilibrium by analyzing a single NMR spectrum since the equilibrium lineshape (Eq. (13) and Eq. (16) in Table 1) can also be fitted to the steady-state lineshape (Eq. (13) and Eq. (15) in Table 1). For further details, see Section S7.3 in SI.

The first special case of three-state kinetics is denoted *half-symmetric* and is shown in Table 1b. In this case, the states *A* and *B* are denoted as $A_1$ and $A_2$, respectively, due to the symmetry in the corresponding chemical species. Hence, the states $A_1$ and $A_2$ are equally populated, and the system is described by three transition rate coefficients: $k_{AC}$, $k_{CA}$ and $k_A$. Populations given by Eqs. (17) in Table 1 already imply equilibrium because they obey the equilibrium conditions in Eq. (11). A steady-state with non-zero net fluxes is not possible for this kinetic scheme. The second special case is a *consecutive* kinetic scheme, which is shown in Table 1c. As this scheme lacks a closed cycle, only equilibrium is possible with populations in Eq. (18) in Table 1.

### Extension to a higher number of states

The derivation of NMR lineshape formulae is straightforward for arbitrary numbers of states and arbitrary spin kinetic schemes, as shown in Section S1.1 in SI. An example of *Mathematica* code for rapid derivation of four-state exchange lineshape is provided in Section S1.2 in SI (we do not show the actual formulae due to their excessive numbers of terms).

We have identified a four-state spin kinetics system in the *cis*-*trans* isomerization process in work on butterfly-shaped overcrowded alkene published by Kartha et al.^32^ It is important to note that the *cis*-*trans* isomerization corresponds to chemical kinetics involving three species (meso *cis* and two chiral *trans* isomers). The transition between *cis* and both *trans* forms is enabled by two independent reversible processes: ‘rim flip’ or ‘double bond flip’, both with different energy barriers. In the original paper, the data were fitted by symmetric two-state exchange lineshapes on two different temperature ranges. We have reproduced the full four-state temperature-dependent spectra using the lineshapes derived in Section S1.2 in SI. Our four-state model reconstructs the experimental data shown in Kartha et al.^32^ with excellent accuracy. We have also compared the two-state model fits used in the original paper to the actual four-state spectral lineshape and discuss the quality of this approximation. Moreover, we have also calculated the two limit cases (low- and high-temperature regimes), where the four-state lineshape can be exactly identified as two-state lineshapes. Detailed description and analysis can be found in SI, Section S8.

## Exchange schemes containing a fast process

**Figure 4:**
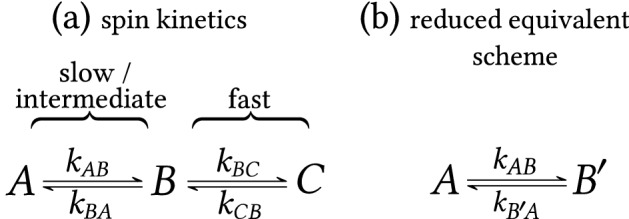
Consecutive three-state exchange containing a fast exchange process. (**a**) Spin kinetics scheme and (**b**) the corresponding reduced equivalent scheme.

Let us consider a consecutive three-state spin kinetics scheme with slow or intermediate exchange between states *A* and *B*, and fast exchange between *B* and *C*, see Fig. 4a.

The two fast-exchanging peaks merge, so that only two peaks can be observed. This situation can be modeled as limit of $k_{BC}, k_{CB} \rightarrow \infty$ in Eq. (13), the ratio $k_{BC} / k_{CB} = p_C / p_B$ is kept constant. The resulting equation is given as

19 $$\begin{aligned} S_{\text{three-state exch.limit}}(\omega )=M_0\frac{\mathscr{P}}{\mathscr{Q}} \,, \end{aligned}$$

where

$$\begin{aligned} M_0&= M_0^A+M_0^B+M_0^C \,, \\ \mathscr {P}&= p_A \frac{p_B \alpha _B+p_C \alpha _C}{p_B+p_C} + (p_B+p_C)\alpha _A + \frac{k_{BA} p_B }{p_B+p_C} + k_{AB} \,, \\ \mathscr {Q}&= \alpha _A \frac{p_B \alpha _B+p_C \alpha _C}{p_B+p_C} + \frac{k_{BA} p_B}{p_B+p_C} \alpha _A + k_{AB} \frac{p_B \alpha _B + p_C \alpha _C}{p_B + p_C} \,. \end{aligned}$$

In comparison with Eq. (7), it becomes obvious that three-state exchange containing a fast process can be modeled as a two-state exchange between states *A* and $B'$ with transition rate coefficients $k_{AB}$ and $k_{B'\!A}$, where $k_{B'\!A}$ has the following relationship to the transition rate coefficient $k_{BA}$ with physical meaning

20 $$\begin{aligned} k_{B'\!A}=k_{BA} \frac{p_B}{p_B+p_C} \,. \end{aligned}$$

The resonant frequency of the state $B'$ is the population weighted average of the frequencies $\omega _B$ and $\omega _C$, in particular $\omega _{B'}=(p_B \omega _B + p_C \omega _C)/(p_B+p_C)$ (by analogy with fast asymmetric two-state exchange). Similarly, it holds that $R_2^{B'}=(p_B R_2^B + p_C R_2^C)/(p_B+p_C)$ for the relaxation rate. We can view the $B'$ state as being merged *B* and *C* since $M_{B'}=M_B+M_C$ (and consequently $p_{B'}=p_B+p_C$). The full kinetic scheme is simplified to a *reduced equivalent scheme* as seen in Fig. 4b. To maintain the transition rate from $B'$ to *A* (one-way population flux, i.e., $k_{B'\!A} p_{B'}$) the same as the transition rate from *B* to *A* (i.e., $k_{\rm BA} p_{\rm B}$) in the original scheme, the equality $k_{B'\!A} p_{B'} = k_{BA} p_B$ must hold. Because $p_{B'}$ is larger than $p_B$, the modified coefficient $k_{B'\!A}$ should be smaller than the original coefficient $k_{BA}$. Note that only the transition rate coefficient *out* of the replacement state $B'$ is modified (as seen in Fig. 4a vs. b). Furthermore, modified transition rate coefficients can also be obtained directly from the kinetics differential equations in Eq. (4), which is illustrated in Section S10 in SI.

In the work of Feng^31^, a simulated three-state exchange (the same as in Fig. 4a) was successfully fitted with a two-state formula. Our approach here using the reduced equivalent scheme can be used to explain these results^31^ and provides an in-depth understanding, see details in SI, Section S9. Another example of a reduced equivalent scheme for three-state exchange with appended fast-exchanging state is given in SI, Section S10.

## Chemical exchange in host–guest complexes

Chemical exchange, as described by the previous schemes and governed by transition rate coefficients $k_{ij}$, comprises transitions between states that can be observed by using 1D NMR measurements. In this paper, we apply terminology from chemical kinetics^33^, where the term ‘rate’ denotes variations of the concentrations of reactants or products with time (the rate is in units of M.s$^{-1}$). In the following discussion, we differentiate *reaction rate* (rate of change between chemical species) from *transition rate* (rate of change between spin states). Finally, reaction and transition rates are directly related, which enables concentration dependence of transition rate coefficients to be determined^17^.

**Figure 5:**
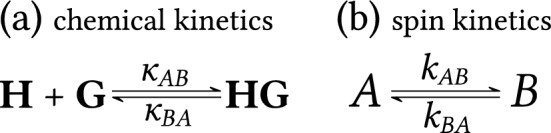
Two-state exchange with 1:1 host–guest binding. Schemes for (**a**) chemical kinetics and (**b**) corresponding spin kinetics of a nucleus located at the host molecule.

Let us consider the 1:1 host(**H**)–guest(**G**) binding in Fig. 5a with an equilibrium constant

21 $$\begin{aligned} K_{{{\textbf {HG}}}} =\frac{[{\textbf {HG}}]}{[{\textbf {H}}] [{\textbf {G}}]} =\frac{\kappa _{AB}}{\kappa _{BA}} \,. \end{aligned}$$

Guldberg-Waage’s law of mass action assumes that the forward and backward rates of a chemical reaction are proportional to the concentrations of reacting molecules,

22a $$\begin{aligned} \text {forward reaction rate}&=\kappa _{AB} [{\textbf {G}}] [{\textbf {H}}] \,, \end{aligned}$$

22b $$\begin{aligned} \text {backward reaction rate}&=\kappa _{BA} [{\textbf {HG}}] \,. \end{aligned}$$

Here we distinguish between *reaction rate coefficients* $\kappa _\bullet$, which describe the kinetics of host–guest binding, and *transition rate coefficients* $k_\bullet$ which describe transitions of a particular spin between corresponding states. Although reaction rate coefficients $\kappa _\bullet$ are usually denoted in the literature as ‘$k_\bullet$’, here we have applied the Greek letter ‘$\kappa$’ to avoid confusion with transition rate coefficients. Reaction rate coefficients are independent of concentration and their temperature dependence is governed by the Eyring equation, in accordance with transition state theory,

23 $$\begin{aligned} \kappa _{ij}=\frac{\eta k_\text {B} T}{h} \exp \left(- \frac{\Delta G_\text {ij}^\ddag }{R T} \right) \,, \end{aligned}$$

where $\Delta G_\text {ij}^\ddag$ is the molar Gibbs free energy of activation between states *i* and *j* (i.e., height of the barrier between states *i* and *j*), $\eta$ is the transition probability, *h* is the Planck constant and *R* is the molar gas constant.

The free host **H** can be assigned to state *A* and the complex **HG** to state *B* ($[A]=[{\textbf {H}}]$ and $[B]=[{\textbf {HG}}]$). The variation of concentration with time of the spin state *B* associated with the **HG** complex, i.e., the transition rate, is given by Fig. 5b and Eq. (4) (multiplied by $[{\textbf {H}}]_\text {t}$ to transform populations to concentrations, subscript ‘t’ denotes total concentration), yielding $\text {d}[B]/\text {d}t = k_{AB} [A] - k_{BA} [B]$. In the latter equation, the two right-hand side terms are identified as forward and backward transition rates, that is

24a $$\begin{aligned} \text {forward transition rate}&=k_{AB} [A] \,, \end{aligned}$$

24b $$\begin{aligned} \text {backward transition rate}&=k_{BA} [B]\, . \end{aligned}$$

A comparison of Eq. (22) with Eq. (24), using the assignment $[A]=[{\textbf {H}}]$ and $[B]=[{\textbf {HG}}]$, implies that $k_{AB}$ depends on free guest concentration unlike $k_{BA}$,

25a $$\begin{aligned} k_{AB}&=\kappa _{AB} [{\textbf {G}}] \,, \end{aligned}$$

25b $$\begin{aligned} k_{BA}&=\kappa _{BA} \, . \end{aligned}$$

The free guest concentration $[{\textbf {G}}]$ and consequently the value of $k_{AB}$ increases with $[{\textbf {G}}]_\text {t}$ and decreases with $[{\textbf {H}}]_\text {t}$. For bimolecular elementary reactions the transition rate coefficients usually depend linearly on concentration, while for unimolecular reaction (i.e., decay of the complex) the transition rate coefficients are independent of concentration.

The above formulas (Eq. (25)) imply that simple 1:1 host–guest binding does not allow (in general) for symmetric two-state exchange because in titration experiments the $p_B/p_A$ ratio always increases upon addition of guest molecules. Only two of the parameters $\kappa _{AB}$, $\kappa _{BA}$ and $K_{{{\textbf {HG}}}}$ are independent. The dependence of $[{\textbf {H}}]$, $[{\textbf {HG}}]$ and $[{\textbf {G}}]$ on the total host and guest concentrations ($[{\textbf {H}}]_\text {t}$ and $[{\textbf {G}}]_\text {t}$) can be expressed in an analytical form for 1:1 host–guest binding, see Eqs. (S41a-c) in SI, Section S11.1. Illustration of a simulated titration of a host with a guest is given in SI, Section S11.2. In the SI, Sections S11.3 and S11.4, we also give an example of a three-state exchange in the competitive host–ligand binding model (two types of ligand), including the lineshapes during a simulated titration experiment.

## Analysis of an actual system exhibiting multi-state exchange

Analytical solutions^1–3,34^ for spectral lineshape have been used for fitting of the two-state exchange, but only numerical methods^4–6,30,31,35^ have been applied in the case of systems containing more states than two. This section illustrates the use and benefits of the analytical treatment of the multi-state chemical exchange scheme containing fast exchange processes (see "Exchange schemes containing a fast process" Section), which reduces the number of fitting parameters.

**Figure 6:**
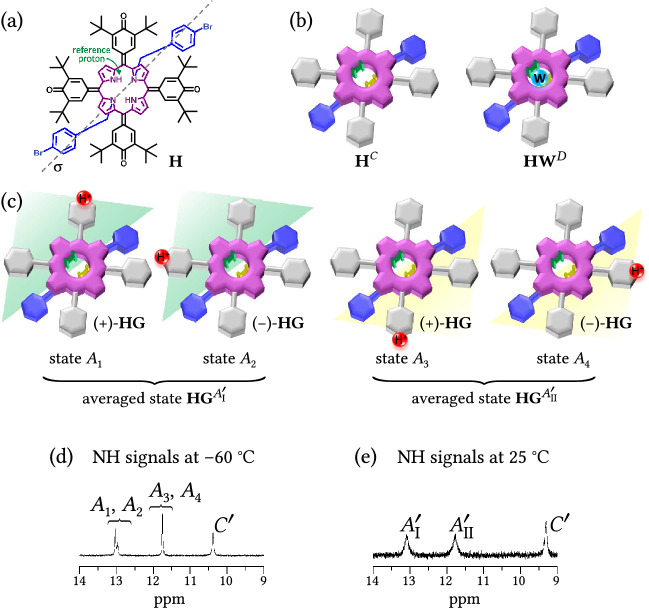
Spin states of central NH protons of di-bromobenzylated oxoporphyrinogen host molecule (**H**). (**a**) Structure of **H**. Bromobenzyl groups are situated behind the molecule. The spin states are described with respect to reference proton denoted by green arrow. (**b**) Schematic representation of free host $\mathrm{{\textbf {H}}}^C$ and host–water complex $\mathrm{{\textbf {HW}}}^D$ inducing the spin states denoted in superscript. (**c**) Protonated host–guest complex in two distinct tautomeric forms ($+$)-**HG** and (−)-**HG** differing in the site of protonation. NH spins of ($+$)-**HG** tautomer can be present in two spin states $\mathrm{{\textbf {HG}}}^{A_1}$ or $\mathrm{{\textbf {HG}}}^{A_3}$ (similarly NH spins of (−)-**HG** can be in states $\mathrm{{\textbf {HG}}}^{A_2}$ or $\mathrm{{\textbf {HG}}}^{A_4}$) depending on the carbonyl protonation proximity to the green-labeled reference proton. Different sites of protonation within the green and yellow zones form the averaged states $A_\text {I}'$ and $A_\text {II}'$, respectively. Guest anion stabilizing the host–guest complex^14^ is not shown since it does not affect the structure of spin states (see details in the text). The superscripts (e.g., **H**$^C$, **HG**$^{A_1}$) denote the spin state with respect to the green-labeled reference NH proton. (**d**,**e**) ^1^H NMR spectra of NH resonances of host **H** ($8.4\times 10^{-4}$ M$^{-1}$, CDCl$_{3}$) with 0.59 equiv. of guest **G** at (**d**) $-60$ $^\circ$C and (**e**) 25 $^\circ$C. In (**d**), the presence of four states $A_1$, ..., $A_4$ of protonated **H** can be recognized (intensity ratio of the peaks at 13.03 and 12.96 ppm is 74:26). In (**e**), only two averaged states $A_\text {I}'$ and $A_\text {II}'$ can be observed.

**Figure 7:**
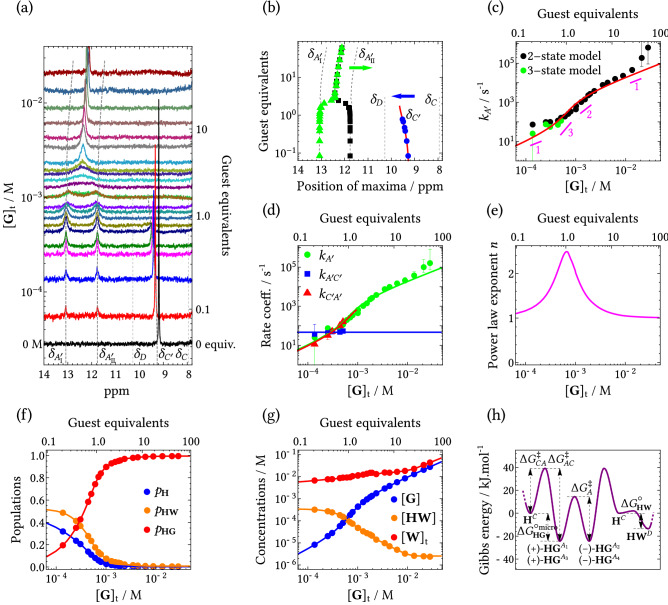
Experimental results for the host–guest system of di-bromobenzylated oxoporphyrinogen with (*R*)-camphorsulfonic acid. (**a**) NH portion of ^1^H NMR spectra of host **H** (initial concentration $6\times 10^{-4}\text { M}$, CDCl$_{3}$) during the titration with guest **G** (*y*-scaling of spectra is adjusted for clarity). Concentration of guest **G** corresponds to the value where the spectrum meets the *y*-axis. (**b**) Apparent positions of peak maxima during the titration, green and blue arrows denote the shift of maxima due to solvent polarity increase and fast exchange between states *C* (free **H**) and *D* (host–water complex **HW**), respectively. Red line is fit of the frequency $\omega _{C'}=p_C \omega _C+p_D \omega _D$ rescaled to ppm. (**c**) Concentration dependence of the transition rate coefficient $k_{A'}$ obtained using two-state lineshape fitting on states $A_\text {I}'$ and $A_\text {II}'$ (black circles) and using three-state lineshape fitting on states $A_\text {I}'$, $A_\text {II}'$ and $C'$ (green circles). The red line is the best fit of the $k_{A'}$ concentration dependence within three-state model (two-state model is used above 1 equiv. due to the disappearance of the *C* state from NMR spectra) using Eqs. (26a) and (S45a,b). Magenta lines denote the slope of the red fitting curve. (**d**) Other transition rate coefficients describing the half-symmetric three-state exchange. The parameter $k_{A'\!C'}$ was fitted and $k_{C'\!A'}$ was calculated ($k_{C'\!A'}=k_{A'\!C'} p_{C'}/ p_{A_\text {I}'}$). (**e**) Concentration dependence of the power law exponent. (**f**) Concentration dependence of populations of host-related species. (**g**) Concentration dependence of free guest, host–water complex (both determined from the model in SI, Section S11.3) and total concentration of water (as determined from water peak integration, red solid line is interpolation). (**h**) Gibbs energy profile of all chemical species as calculated from the Eyring equation, Eq. (23), from the reaction rate coefficients at $T=298\text { K}$ and setting the transition probability $\eta =1$. Barriers $\Delta G_{CD}^\ddagger$ and $\Delta G_{DC}^\ddagger$ were not determined, since the corresponding exchange process was too fast. The Larmor frequencies of states $A_\text {I}'$ and $A_\text {II}'$ are not unequivocally assigned with respect to the structures in Fig. 6c. Error bars in (**c**) and (**d**) denote maximum errors, see discussion in Section S12 in SI.

The di-bromobenzylated oxoporphyrinogen host (**H**), i.e., $\hbox {N}_{21}$,$\hbox {N}_{23}$-*bis*(4-bromobenzyl)-5,10,15,20-*tetrakis* (3,5-di-*t*-butyl-4-oxocyclohexadien-2,5-ylidene)porphyrinogen, Fig. 6a, was synthesized by previously reported method^36,37^. The host **H** is protonated by (*R*)-camphorsulphonic acid guest (**G**) at one of its hemiquinonoid sites while forming a host–guest complex (**HG**) to stabilize the protonated cation. Full NMR spectra and details of the corresponding titration experiment are given in our previous paper^14^. The central NH protons of the host are subject to chemical exchange between six environments, which is experimentally manifested in its NMR spectrum as three resonances (some of the resonances are already merged due to fast exchange, see below) in the titration spectra at low $[{\textbf {G}}]_\text {t}$, see Fig. 7a. At low $[{\textbf {G}}]_\text {t}$ the three observable resonances are well separated and the exchange between them is in a slow regime. The peak at 9.3 ppm, denoted as state $C'$, vanishes at 1 guest equivalent (equivalents defined as $[{\textbf {G}}]_\text {t}/[{\textbf {H}}]_\text {t}$). The state $C'$ actually comprises substates *C* and *D* averaged due to fast chemical exchange, see Fig. 6b. These substates correspond to free host and its complex with water, respectively (details are discussed below). The two resonances at 13.1 and 11.8 ppm correspond to two equally populated states denoted as $A_\text {I}'$ and $A_\text {II}'$, respectively. The state $A_\text {I}'$ comprises substates $A_1$ and $A_2$, the state $A_\text {II}'$ comprises substates $A_3$ and $A_4$, each two substates are averaged due to the fast exchange. States $A_1$, ..., $A_4$ of the green-labeled reference NH spin in Fig. 6c correspond to protonated host with protonation at four different C=O sites. States $A_1$ and $A_3$ correspond to (${+}$)-**HG** tautomer and states $A_2$ and $A_4$ to (−)-**HG** tautomer. The labels ($+$) and (−) have been assigned arbitrarily (without the influence of guest anion, the protonated species (${+}$)-**H**^+^ and (−)-**H**^+^ are mutual mirror images, i.e., enantiomers).

Binding of the guest counteranion at the central NH site influences the Larmor frequency of the states $A_1$, ..., $A_4$ through so-called ‘chiral field’^34^ caused by fast movement of the guest anion near the NH site (the charged species interact strongly together due to hydrophobicity of the CDCl$_3$ solvent). All states $A_1$, ..., $A_4$ are directly observed at low temperature as shown in Fig. 6d. Since the chiral field is different in (${+}$)-**HG** and (−)-**HG** (due to the absence of mirror symmetry of the guest), Larmor frequency of state $A_1$ differs from that of $A_2$, although Larmor frequencies of $A_3$ and $A_4$ are coincidentally similar. At room temperature, only averaged states $A_\text {I}'$ and $A_\text {II}'$ are detected, see Fig. 6e. Considering the mirror symmetry plane $\sigma$ of the host (shown in Fig. 6a), the reference proton is in state $A_\text {I}'$ when the protonation is at the same side of the molecule and in state $A_\text {II}'$ when the protonation is on the other side. We were not able to unequivocally assign states $A_1$, ..., $A_4$ (corresponding to structures in Fig. 6c) to particular resonances in Fig. 6d. However, this information is not essential for analyzing the system’s kinetics. Therefore, we assumed that Larmor frequency of the reference NH spin (marked in green in Fig. 6c) is likely similar when the protonation is in its vicinity (i.e., states $A_1$ and $A_2$) contrary to protonation across the symmetry plane (i.e., states $A_3$ and $A_4$), which results in assignment shown in Fig. 6d. Enantiomeric excess of the chiral guest molecule does not influence the spin states (titration with (*rac*)-CSA produced the same spectral behavior of the NH resonances^14^). Due to the symmetry, both states $A_\text {I}'$ and $A_\text {II}'$ have the same populations. At higher guest concentrations, the resonances in states $A_\text {I}'$ and $A_\text {II}'$ start to coalesce and enter intermediate then fast exchange regimes.

The observed spectral behavior can be described (in terms of reduced equivalent scheme) as the half-symmetric three-state exchange with states $A_\text {I}'$, $A_\text {II}'$ and $C'$ (Table 1b), which is characterized by two independent transition rate coefficients $k_{A'}$ and $k_{A'\!C'}$ ($k_{C'\!A'}=k_{A'\!C'} p_{C'}/ p_{A_\text {I}'}$ is not independent as follows from Eqs. (17a,b) in Table 1). The corresponding populations are $p_{A_\text {I}'} = p_{A_1}+p_{A_2}$, $p_{A_\text {II}'} = p_{A_3}+p_{A_4}$ and $p_{C'} = p_C+p_D$. Resonance positions $\delta _{A_\text {I}'}$ and $\delta _{A_\text {II}'}$ shift upfield (to lower ppm values) at higher acid concentrations due to the increase in polarity of the medium upon addition of acid, see green arrow in Fig. 7b. The resonance position $\delta _{C'}$ shifts downfield (to larger ppm values) during the titration (while $\delta _{A_\text {I}'}$ and $\delta _{A_\text {II}'}$ still remain constant), see blue arrow in Fig. 7b. This is a direct evidence that state $C'$ consists of two substates *C* and *D* because the three-state solution in Eq. (13) does not allow shifts of peak maxima in the slow exchange regime. In the current model, the peak due to state $C'$ can shift when populations $p_C$ and $p_D$ change ($\delta _{C'} = p_C \delta _C + p_D \delta _D$). It is already known that the host molecule can bind water with a binding constant $K_{{{\textbf {HW}}}}=240 \pm 35$ M$^{-1}$^38^. During experiments it is not reasonably possible to prepare $\hbox {CDCl}_{3}$ solutions of **H** containing no residual water. Moreover, small quantities of water are added together with the hydrophilic guest during the titration process. Hence, the spin state *D* most likely corresponds to a host–water complex (**HW**). Competitive binding of guest and water is assumed^38^, i.e., complexation with one ligand precludes binding of the other (see Sections S11.3 and S11.4). Because of the high mobility of water (small molecule) and low value of $K_{{{\textbf {HW}}}}$ we assume infinitely fast exchange between states *C* and *D*. To summarize, the chemical kinetics description of this system includes competitive host–guest and host–water binding. The observed spin exchange kinetics (for host NH signals) can be described using the half-symmetric three-state model. Fast exchange effectively reduces the number of states to three, enabling the use of the reduced equivalent scheme.

**Figure 8:**
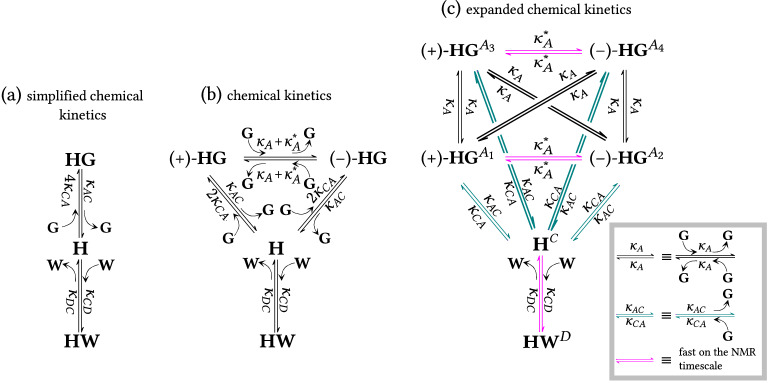
Chemical kinetics schemes for the multi-state system of di-bromobenzylated oxoporphyrinogen (host **H**) in the presence of two ligands, (*R*)-camphorsulfonic acid (ligand **G**) and water (ligand **W**). (**a**) Simplified chemical kinetics scheme corresponding to 1:1 **H**:**G** binding with competitive 1:1 **H**:**W** binding (see Section S11.3 in SI for details). (**b**) Chemical kinetics scheme describing interconversion of all distinguishable chemical species. (**c**) Expanded chemical kinetics scheme, equal to the full spin kinetics scheme. All relevant molecular processes and their reaction rate coefficients are shown. Processes denoted by magenta arrows are fast on the NMR timescale.

Figure 8 shows three different levels of description of chemical kinetics in this system, and the underlying equations are given in SI, Section S11.5. Figure 8a shows the simplified chemical kinetics of the processes involved assuming the presence of host molecule as three distinct chemical species, **H**, **HG** and **HW**. The simplified chemical kinetics scheme is sufficient to describe the competitive host–ligand binding characterized by equilibrium constants $K_{{{\textbf {HG}}}}$ and $K_{{{\textbf {HW}}}}$, the corresponding equations are shown in SI, Section S11.3. The constant $K_{{{\textbf {HG}}}}$ accounts for the protonation of any of the four sites. The low-temperature spectrum in Fig. 6d suggests that the sites are not equivalent since states $A_1$ and $A_2$ are not equally populated with intensity ratio 74:26. The populations cannot be determined at room temperature. However, we can treat the sites as equivalent (with equal populations) at room temperature with reasonable accuracy since the populations tend to equalize with increasing temperature. Hence, the reaction rate coefficient $4 \kappa _{CA}$ in Fig. 8a contains an integer prefactor to account for the four equivalent protonation sites. Protonation of one particular site is characterized by $K_{{{\textbf {HG}}}}^\text {micro} = K_{{{\textbf {HG}}}}/4$. The microscopic equilibrium constant $K_{{{\textbf {HG}}}}^\text {micro}$ is also equal to the ratio of reaction rate coefficients for the molecular processes of protonation and deprotonation, i.e., $K_{{{\textbf {HG}}}}^\text{micro}=\kappa _{CA}/\kappa _{AC}$. In fact, the protonation forms two different tautomeric species (${+}$)-**HG** and (−)-**HG** as discussed above, see Fig. 6c. This situation is captured by the chemical kinetics scheme in Fig. 8b. Both tautomers can be formed in two different ways, hence the integer prefactor in $2 \kappa _{CA}$. Other processes denoted as ‘guest-mediated prototropic tautomerization’, characterized by $\kappa _{A}$ and $\kappa _{A}^*$, are present in this scheme. These interconvert spin states $A_1$, ..., $A_4$ and hence also (${+}$)-**HG** and (−)-**HG** chemical species, and occur when an incoming acid guest protonates the host while the initial protonation is removed. When the protonation is interchanged at the same side of the molecule with respect to the mirror symmetry plane $\sigma$ (see Fig. 6a), the process is described by $\kappa _{A}^*$ ($A_1 \leftrightarrow A_2$ or $A_3 \leftrightarrow A_4$ in spin kinetics) otherwise by $\kappa _{A}$. This interconversion between the two tautomeric forms can occur in two ways implying overall reaction rate coefficient $\kappa _{A}+\kappa _{A}^*$ (Fig. 8b). It is a second order reaction since the guest molecule has to collide with the host–guest complex. Details of these tautomerization processes, including structure of transition states, are given in SI Section S11.6.

The chemical kinetics scheme can be further expanded to remove degeneracy from all microstates, see in Fig. 8c. Here, all chemically distinct species in all spin states (as listed in Fig. 6) are shown as separate entities, so that no prefactors or sums of reaction rate coefficients are present for the reaction rate coefficients. Corresponding spin states are denoted in superscripts at the chemical species, e.g., **H**$^C$, **HG**$^{A_1}$. The processes characterized by $\kappa _A^*$ have low energy barriers and therefore are fast at room temperature (fast processes are denoted by magenta arrows in Fig. 8c). This makes $\kappa _A^*$ indeterminable by lineshape analysis. The expanded chemical kinetics scheme is equal to the full spin kinetics scheme and forms a basis for the connection between chemical kinetics and observed spin kinetics as explained in the next paragraph.

**Figure 9:**
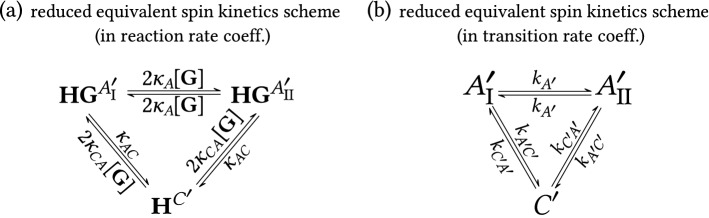
Reduced equivalent spin kinetics schemes for the multi-state system of di-bromobenzylated oxoporphyrinogen (host **H**) in the presence of two ligands, (*R*)-camphorsulfonic acid (ligand **G**) and water (ligand **W**). Schemes refer to the central NH protons of the host molecule. (**a**) Spin kinetics in terms of reaction rate coefficients as obtained from contraction of the scheme in Fig. 8c. (**b**) Corresponding spin kinetics in terms of transition rate coefficients. It has the form of half-symmetric three-state exchange. Comparison with (**a**) gives the relationship between transition and reaction rate coefficients in Eq. (26). This scheme represents experimentally observed spin kinetics. It was used for lineshape fitting, see SI Section S12 for details.

Due to fast exchange (large $\kappa _A^*$, $\kappa _{CD}$ and $\kappa _{DC}$), the kinetics scheme in Fig. 8c must be contracted from six to three states, which results in the spin kinetics scheme in Fig. 9a. Then this scheme expressed in reaction rate coefficients can be directly compared to the corresponding scheme expressed in transition rate coefficients in Fig. 9b, and subsequently, the relations between reaction and transition rate coefficients can be established. In addition, the concentration dependence of the transition rate coefficients is obtained. Details of this procedure are described in SI, Section S11.5. The resulting equations are

26a $$\begin{aligned} k_{A'}&=2\kappa _A [{\textbf {G}}] \,, \end{aligned}$$

26b $$\begin{aligned} k_{A'\!C'}&=\kappa _{AC} \,, \end{aligned}$$

26c $$\begin{aligned} k_{C'\!A'}&= 2\kappa _{CA}[{\textbf {G}}] \, . \end{aligned}$$

The transition rate coefficient $k_{A'\!C'}$, which corresponds to decay of **HG** complex, is independent of concentration. On the other hand, formation of a complex is a bimolecular reaction, therefore $k_{C'\!A'}$ is proportional to $[{\textbf {G}}]$. Also, the guest-mediated prototropic tautomerization is a bimolecular reaction (based on the suggested reaction scheme in SI, Section S11.6), implying $k_{A'}$ is proportional to $[{\textbf {G}}]$.

The use of three-state reduced equivalent spin kinetics scheme enables us to describe the observed spectra with only two transition rate coefficients $k_{A'}$ and $k_{A'\!C'}$. At guest concentrations higher than 1 equiv. the two-state model with transition rate coefficient $k_{A'}$ is sufficient because the state $C'$ is unpopulated. During the fitting procedure, the parameter $\delta _{C'}$, which describes the averaged frequency of the state $C'$, is fixed exactly at the corresponding peak position in the spectra, frequencies of the states $A_\text {I}'$ and $A_\text {II}'$ are also fixed, for details see Section S12 in SI. To show the actual usefulness of the three-state model, we have also fitted the states $A_\text {I}'$ and $A_\text {II}'$ with the two-state model over the concentration range studied (fitting parameter $k_{A'}$). From Fig. 7c it is obvious that the three-state model systematically shifts $k_{A'}$ to lower values than obtained using the two-state model (presence of the third state $C'$ causes broadening of the peaks due to states $A_\text {I}'$ and $A_\text {II}'$). An example of actual fitted spectra is shown in Fig. S24a,c in SI.

After the values of $k_{A'}$ and $k_{A'\!C'}$ were obtained from the raw spectra (green and blue points in Fig. 7d), fitting of the concentration dependence of $k_{A'}$ (using Eq. (26a)) was conducted simultaneously with other changes in spectra. This is discussed together with other technical details of the fitting procedure in SI, Section S12. The resulting fitted curve for $k_{A'}$ (red line in Fig. 7c) describes the experimental values from the three-state exchange model with good accuracy and confirms the assumption of proportionality to $[{\textbf {G}}]$ for the guest-mediated prototropic tautomerization, expressed in Eq. (26a). The value of equilibrium constant $K_{{{\textbf {HG}}}}=(7.4\pm 3.0)\times 10^4 \text { M}^{-1}$ was also obtained. This value is comparable with the result of our previous analysis^14^ $K_{{{\textbf {HG}}}} = (8.0 \pm 5.0) \times 10^4$ M$^{-1}$, which did not take into account the competitive binding of water and the concentration dependence of $k_{A'}$. The concentration dependence of $k_{C'\!A'}$ was determined using the formula $k_{C'\!A'}=2 \kappa _{CA} [{\textbf {G}}] = K_{{{\textbf {HG}}}} k_{AC} [{\textbf {G}}]/2$, see Fig. 7d. The value of $k_{C'\!A'}$ is calculated directly from fitted values of $k_{A'\!C'}$ up to 1 equiv. (red points in Fig. 7d). For higher guest concentrations, the population of state $C'$ is negligible, and values of $k_{A'\!C'}$ could not be determined by three-state lineshape fitting. The fitting procedure also provides concentrations of all species present in the sample, see Fig. 7f,g. Values of equilibrium constants and reaction rate coefficients are listed in Table 2 and an overview of all parameters used during the fitting procedure is given in Table S6 in SI.

The concentration dependence of $k_{A'}$ can also be expressed in the form of a power law as $k_{A'} \propto [{\textbf {G}}]_\text {t}^n$ (for constant $[{\textbf {H}}]_\text {t}$). The exponent *n* can easily be extracted from the log-log plot in Fig. 7c as the gradient (first derivative) of the red curve. The concentration dependence of the power law exponent is shown in Fig. 7e. It can be seen that for low and high guest concentrations $n=1$. However, between these limit cases, the power law exponent reaches values over $n=3$. It is interesting to mention that the $k_{A'}$ dependence on free guest concentration $[{\textbf {G}}]$ has a simple linear relationship (see Eq. (26a)) while its dependence on total guest concentration $[{\textbf {G}}]_\text {t}$ has a nonlinear form with the largest deviation from linearity around 1 equiv. of total guest concentration (Fig. 7e).

The chemical kinetics, according to Fig. 8b, can also be viewed in terms of the Gibbs energy landscape as shown in Fig. 7h. The energy barriers are calculated from the corresponding reaction rate coefficients using the Eyring equation, Eq. (23), at $T=298$ K with the transition probability $\eta =1$. Standard reaction Gibbs energies for both **HG** and **HW** complexes were calculated from the equilibrium constants $K_{{{\textbf {HG}}}}^\text {micro}$ and $K_{{{\textbf {HW}}}}$, respectively. All parameters of the Gibbs energy profile are listed in Table 2. The barrier between states *C* and *D* was not determined since it is very low (i.e., fast exchange regime).

**Table 2:** Parameters of Gibbs energy profile at $T=298$ K.

Equilibrium constants/reaction rate coefficients		Gibbs energy parameters (kJ mol$^{-1}$)		Mutual relationship
$K_{{{\textbf {HG}}}}$	$(7.4\pm 3.0)\times 10^4 \text { M}^{-1}$	$\Delta G_{{\textbf {HG}}}^\circ$	$-27.8\pm 1.0$	$\Delta G_{{\textbf {HG}}}^{\circ } = -RT\ln K_{{{\textbf {HG}}}}$
$K_{{{\textbf {HG}}}}^\text {micro}$	$(1.9\pm 0.8)\times 10^4 \text { M}^{-1}$	$\Delta G_{{\textbf {HG}}}^{\circ \text {micro}}$	$-24.3\pm 1.0$	$\Delta G_{{\textbf {HG}}}^{\circ {micro}} = -RT\ln K_{{{\textbf {HG}}}}^\text {micro}$
$K_{{{\textbf {HW}}}}$	$240\pm 35$ M$^{-1}$	$\Delta G_{{\textbf {HW}}}^\circ$	$-13.6\pm 0.4$	$\Delta G_{{\textbf {HW}}}^{\circ } = -RT\ln K_{{{\textbf {HW}}}}$
$\kappa _{AC}$	$47\pm 6$ s$^{-1}$	$\Delta G_{AC}^\ddagger$	$63.4\pm 0.3$	Eyring equation$^a$
$\kappa _{CA}$	($9\pm 4)\times 10^5$ M$^{-1}$s$^{-1}$	$\Delta G_{CA}^\ddagger$	$39.0\pm 1.1$	Eyring equation$^a$
$\kappa _{A}$	$(10\pm 1)\times 10^5$ M$^{-1}$s$^{-1}$	$\Delta G_{A}^\ddagger$	$38.7\pm 0.2$	Eyring equation$^a$

$^a$In the Eyring equation, the assumption for transition probability $\eta =1$ was used.

## Conclusion

In the theoretical part of this work, we have summarized the method of analytical calculation of spectral exchange lineshapes for general *N*-state spin kinetics (in the absence of J-coupling). We have reviewed the analytical solution for the two-state case and calculated the solution for the three-state case. Using the analytical solution in the case of symmetric two-state exchange, corrections to the well-known formula for the coalescence point are given, and the concept of coalescence has also been generalized to the asymmetric case. Several special cases of two-state, three-state and four-state spin kinetics and their relevance to host–guest binding or isomerization processes, including examples from literature, have been investigated in detail. These examples illustrate the importance of differentiating between ‘reaction rate coefficients’, which describe chemical kinetics, and ‘transition rate coefficients’, which describe spin kinetics. We have emphasized the possibility of multiple processes occurring between two states and illustrate this using several literature examples, and discussed the possible presence of a steady-state mode (constant populations but non-zero population flux) in some kinetic schemes. An analysis of the Michaelis-Menten mechanism of enzyme-catalyzed reactions has been provided as an example of a system where a steady-state can be achieved. An interesting result of our theoretical analysis, which employs the analytical spectral lineshapes, is the introduction of the concept of ‘reduced equivalent schemes’ for spin kinetics containing a fast-exchanging state. These schemes contain fewer states with modified transition rate coefficients, which still contain a physical meaning. A procedure for their construction has been provided together with several literature illustrations.

In the experimental part, a system consisting of di-bromobenzylated oxoporphyrinogen host complexed with (*R*)-camphorsulfonic acid guest in the (unavoidable) presence of water has been analyzed quantitatively using NMR lineshape fitting. A competitive host–ligand binding model with multi-state exchange was applied to describe the chemical kinetics and spin kinetics of the central NH reference proton. The model accounts for the concentration dependence of transition rate coefficients. Overall, the methods presented in this work can be used to describe molecular kinetics in a wide range of interesting systems with a variety of intra- or intermolecular processes. Analytical solutions for NMR exchange lineshapes allow construction of reduced equivalent schemes for the spin kinetics and fitting of the experimentally observed spectra. Thus, the possible applications of this work range from simple chemical systems, such as those involving host–guest binding, to increasingly elaborate systems, such as those involving enzymatic reactions or nontrivial conformational dynamics of proteins or other complex molecules.

## Supplementary information

contains additional details concerning multi-state NMR exchange: derivation of analytical solutions for spectral lineshapes and codes for computer implementation; analysis of two-state exchange with two processes; analysis of steady-state modes; analysis of coalescence in two-state exchange; description of models for the di-bromobenzylated oxoporphyrinogen host–guest system and in-depth discussion of the lineshape fitting procedure. In addition, one *Excel* spreadsheet and two *Mathematica* notebooks (also in pdf versions), illustrating data fitting, are included. The material is available free of charge at https://doi.org/10.1038/s41598-022-20136-4.

## Supplementary Information

Supplementary Information 1.

Supplementary Information 2.

Supplementary Information 3.

Supplementary Information 4.

Supplementary Information 5.

Supplementary Information 6.

## Data Availability

The majority of data generated or analyzed during this study are included in this published article and its supplementary information files: $1\times$ pdf file, $1\times$ *Excel* spreadsheet, and $2\times$ *Mathematica* notebooks (and their pdf versions). Raw NMR spectra used during the current study are available from the corresponding author on reasonable request.
