# Supplementary material for: NMR lineshape analysis using analytical solutions of multi-state chemical exchange with applications to kinetics of host–guest systems
Source: Sci Rep. 2022 Oct 17;12:17369. doi: 10.1038/s41598-022-20136-4 (PMC9576801; doi:10.1038/s41598-022-20136-4)
Supplement: Supplementary file 1 — Supplementary Information 1. [file 41598_2022_20136_MOESM1_ESM.pdf]

# SUPPLEMENTARY INFORMATION
# NMR lineshape analysis using analytical solutions of multi-state chemical exchange with applications to kinetics of host–guest systems

**Václav Březina[1,2], Lenka Hanyková[2], Nadiia Velychkivska[1,3], Jonathan P. Hill[1], and Jan Labuta[1,*]**

[1]International Center for Materials Nanoarchitectonics (WPI-MANA), National Institute for Materials Science (NIMS), 1-1 Namiki, Tsukuba, Ibaraki 305-0044 (Japan)
[2]Faculty of Mathematics and Physics, Charles University, V Holešovičkách 2, 180 00 Prague 8 (Czech Republic)
[3]Institute of Macromolecular Chemistry, Academy of Sciences of the Czech Republic, Heyrovsky Sq. 2, 162 06 Prague 6 (Czech Republic)
[*]Labuta.Jan@nims.go.jp

## Contents

S1 Derivation of a multi-state chemical exchange lineshape . . . 2
S1.1 General mathematical derivation . . . 2
S1.2 Rapid derivation of four-state lineshape in *Mathematica* . . . 3
S2 Real (absorption) part of analytical solutions . . . 4
S3 *MATLAB* codes for exchange lineshapes . . . 5
S4 Spectral lineshape in limit cases . . . 8
S5 Coalescence . . . 9
S5.1 Expressions for coalescence in symmetric two-state exchange . . . 9
S5.2 Coalescence in asymmetric two-state exchange . . . 10
S6 Two-state exchange with two processes . . . 12
S6.1 Basic relations . . . 12
S6.2 Catalyzed chemical reaction . . . 14
S6.3 Correlated rotation of dimesityl rings . . . 16
S7 Notes on lineshape fitting procedure . . . 17
S7.1 Equilibrium, steady-state and time-dependent population modes . . . 17
S7.2 Interdependency of parameters for general *N*-state exchange . . . 17
S7.3 Steady-state lineshape in three-state exchange . . . 19
S7.4 Limitations of lineshape analysis . . . 20
S8 Four-state exchange in the system of Kartha et al.[S1] . . . 21
S9 Explanation of fitting results in work by Feng et al.[S2] . . . 23
S10 Three-state exchange with appended fast exchanging state . . . 25
S11 Host–guest binding models . . . 27
S11.1 1:1 host–guest binding . . . 27
S11.2 Two-state chemical exchange and 1:1 host–guest binding . . . 27
S11.3 Competitive host–ligand binding . . . 28
S11.4 Three-state chemical exchange and competitive host–ligand binding . . . 29
S11.5 Kinetics of the di-bromobenzylated oxoporphyrinogen system . . . 30
S11.6 Prototropic tautomerization processes . . . 34
S12 Fitting of experimental data . . . 37
References . . . 39

## S1 Derivation of a multi-state chemical exchange lineshape

### S1.1 General mathematical derivation

In literature reports,[S3–S6] the differential Bloch-McConnell equations

$$\frac{\mathrm{d}}{\mathrm{d}t}\mathbf{M}_{xy}(t) = (i\mathbf{L} - \mathbf{R_2} + \mathbf{K})\mathbf{M}_{xy}(t) \tag{S1}$$

are usually solved using matrix exponential involving matrix diagonalization. Subsequently, the time-dependent magnetization vector $\mathbf{M}_{xy}(t)$ is converted to the frequency domain using FT. However, this method already becomes quite inconvenient for the case of an asymmetric two-state exchange. Therefore, other studies[S2, S7, S8] use Eq. (S1) in the frequency domain for numerical simulations of exchange spectra. FT converts the differential Bloch-McConnell equations into algebraic equations, which can be then solved even analytically using standard methods.

The following derivation holds for an arbitrary number of states and arbitrary kinetic schemes. First, FT is applied to Eq. (S1). Note that the signal from the spectrometer is not actually infinite, but it is equal to zero for $t < 0$. Therefore, the functions $f^j(t) = \theta(t)M^j_{xy}(t)$ should be transformed instead of $M^j_{xy}(t)$, $j = A, B, \ldots$ . The function $\theta(t)$ is the Heaviside step function, equal to zero for $t < 0$ and unity otherwise. The left-hand side of Eq. (S1) multiplied by the Heaviside function can be rewritten using the Dirac delta function,

$$\theta(t)\frac{\mathrm{d}M^j_{xy}(t)}{\mathrm{d}t} = \frac{\mathrm{d}}{\mathrm{d}t}\left[\theta(t)M^j_{xy}(t)\right] - M^j_{xy}(t)\frac{\mathrm{d}\theta(t)}{\mathrm{d}t} = \frac{\mathrm{d}f(t)}{\mathrm{d}t} - M^j_{xy}(t)\delta(t)\,. \tag{S2}$$

FT of the first term on the right-hand-side reads

$$\widehat{\frac{\mathrm{d}f(t)}{\mathrm{d}t}} = i\omega\hat{f}(\omega) \tag{S3}$$

and FT of the second term is

$$\int_{-\infty}^{+\infty} M^j_{xy}(t)\delta(t)e^{-i\omega t}dt = M^j_{xy}(t=0)\,. \tag{S4}$$

Without loss of generality, let us assume that the initial transverse magnetization is real and positive, $M^j_{xy}(t=0) = M^j_0$. Using Eqs. (S2), (S3) and (S4), FT of Eq. (S1) is as follows

$$[i(\omega\mathbf{1} - \mathbf{L}) + \mathbf{R} - \mathbf{K}]\begin{pmatrix}\hat{f}_A(\omega)\\ \hat{f}_B(\omega)\\ \vdots\end{pmatrix} = \begin{pmatrix}M^A_0\\ M^B_0\\ \vdots\end{pmatrix}, \tag{S5}$$

where $\mathbf{1}$ is the unit matrix, $\mathbf{L} = \mathrm{diag}(\omega_A, \omega_B, \ldots)$, $\mathbf{R} = \mathrm{diag}(R^A_2, R^B_2, \ldots)$ and $\mathbf{K}$ is the appropriate kinetic matrix ('diag(•)' represents a diagonal matrix with elements indicated in brackets). Using the definition $\alpha_j = R_j + i(\omega - \omega_j)$ we can rewrite Eq. (S5) as

$$[\mathbf{A} - \mathbf{K}]\begin{pmatrix}\hat{f}_A(\omega)\\ \hat{f}_B(\omega)\\ \vdots\end{pmatrix} = M_0\begin{pmatrix}p_A\\ p_B\\ \vdots\end{pmatrix}, \tag{S6}$$

where $\mathbf{A} = \mathrm{diag}(\alpha_A, \alpha_B, \ldots)$ and $M^j_0 = M_0 p_j$.

Solution of this set of linear equations for $\hat{f}_\bullet(\omega)$ is straightforward. Resulting complex NMR signal is detected as the sum of all components,

$$S(\omega) = \hat{f}_A(\omega) + \hat{f}_B(\omega) + \ldots\,. \tag{S7}$$

An example of the implementation of this analytical procedure to obtain four- and five-state exchange spectral lineshape is shown in the next section. The derivation of the analytical solution provides a scheme for the numerical simulation of the spectrum.[S2, S7] By multiplying Eq. (S6) by $[\mathbf{A} - \mathbf{K}]^{-1}$ from left we can calculate the function values of $\hat{f}_j(\omega)$ for a fixed $\omega$. Hence, for $N$ states, inversion of an $N \times N$ matrix is required for each frequency value. Implementation of the numerical solution in *MATLAB* code is shown in Sec. S3.

A steady-state approximation (in the time domain) for continuous-wave NMR spectroscopy[S9–S11] provides an equation fully analogous to Eq. (S6) without a requirement to apply FT. However, the angular frequency $\omega$ in those derivations stands for the frequency of applied continuous radiofrequency field, not for the frequency of the detected signal (as in pulse NMR approach).

### S1.2 Rapid derivation of four-state lineshape in *Mathematica*

Here we show a "copy-paste" code in the *Mathematica* software to rapidly derive the four- and five-state exchange spectral lineshapes. However, it can be readily extended to an arbitrary number of states. We do not show the actual formulae generated by the code due to the excessive number of terms contained.

```
 (* four-state exchange lineshape *)
Kmatrix = {{-kAB - kAC - kAD, kBA, kCA, kDA}, {kAB, -kBA - kBC - kBD, kCB, kDB},
{kAC, kBC, -kCA - kCB - kCD, kDC}, {kAD, kBD, kCD, -kDA - kDB - kDC}};
Amatrix = DiagonalMatrix[{αA, αB, αC, αD}];
(* three following command lines: keeping together the terms on diagonal (βj) of the matrix B=A-K
reduces the size of the final symbolic expression *)
Bmatrix = Amatrix - Kmatrix;
betaRule = MapThread[Rule[#1, #2] &,
{{βA, βB, βC, βD}, Diagonal[Bmatrix]}]; (* later restores the diagonal values of B *)
Bmatrix = Bmatrix - DiagonalMatrix[Diagonal[Bmatrix]] +
  DiagonalMatrix[{βA, βB, βC, βD}]; (* replaces diagonal values with βj *)
{fA, fB, fC, fD} = M0 Inverse[Bmatrix].{pA, pB, pC, pD};
fourStateLineshapeComplex = (Total[{fA, fB, fC, fD}] // Together) /. betaRule;
alphaRule = {αA -> RA + I (ω - ωA), αB -> RB + I (ω - ωB),
αC -> RC + I (ω - ωC), αD -> RD + I (ω - ωD)};
fourStateLineshapeComplex = fourStateLineshapeComplex /. alphaRule; (* substitute for αj *)
(* in order to obtain the absorption part of the spectrum, numerical values of the parameters
    must be inserted and then the real part taken, see the example in the following lines *)
(* absorptionSpectrum = fourStateLineshapeComplex /. {ω -> Range[4000],
    ωA -> 1000., ωB -> 1500., ωC -> 2500., ωD -> 3000.,
    M0 -> 1., pA -> 1/4, pB -> 1/4, pC -> 1/4, pD -> 1/4,
    kAB -> 30., kBA -> 30., kAC -> 30., kCA -> 30., kAD -> 30., kDA -> 30.,
    kBC -> 30., kCB -> 30., kBD -> 30., kDB -> 30., kCD -> 30., kDC -> 30.,
    RA -> 30., RB -> 30., RC -> 30., RD -> 30.};
absorptionSpectrum = Re[absorptionSpectrum]; *)


 (* five-state exchange lineshape *)
Kmatrix = {{-kAB - kAC - kAD - kAE, kBA, kCA, kDA, kEA}, {kAB, -kBA - kBC - kBD - kBE, kCB, kDB, kEB},
{kAC, kBC, -kCA - kCB - kCD - kCE, kDC, kEC}, {kAD, kBD, kCD, -kDA - kDB - kDC - kDE, kED},
{kAE, kBE, kCE, kDE, -kEA - kEB - kEC - kED}};
Amatrix = DiagonalMatrix[{αA, αB, αC, αD, αE}];
(* three following command lines: keeping together the terms on diagonal (βj) of the matrix B=A-K
reduces the size of the final symbolic expression *)
Bmatrix = Amatrix - Kmatrix;
betaRule = MapThread[Rule[#1, #2] &,
{{βA, βB, βC, βD, βE}, Diagonal[Bmatrix]}]; (* later restores the diagonal values of B *)
Bmatrix = Bmatrix - DiagonalMatrix[Diagonal[Bmatrix]] +
  DiagonalMatrix[{βA, βB, βC, βD, βE}]; (* replaces diagonal values with βj *)
{fA, fB, fC, fD, fE} = M0 Inverse[Bmatrix].{pA, pB, pC, pD, pE};
fiveStateLineshapeComplex = (Total[{fA, fB, fC, fD, fE}] // Together) /. betaRule;
alphaRule = {αA -> RA + I (ω - ωA), αB -> RB + I (ω - ωB),
αC -> RC + I (ω - ωC), αD -> RD + I (ω - ωD), αE -> RE + I (ω - ωE)};
fiveStateLineshapeComplex = fiveStateLineshapeComplex /. alphaRule; (* substitute for αj *)
(* in order to obtain the absorption part of the spectrum, numerical values of the parameters
    must be inserted and then the real part taken, see the example in the following lines *)
(* absorptionSpectrum = fiveStateLineshapeComplex /. {ω -> Range[4000],
    ωA -> 1000., ωB -> 1500., ωC -> 2500., ωD -> 3000., ωE -> 3300.,
    M0 -> 1., pA -> 1/4, pB -> 1/4, pC -> 1/4, pD -> 1/8, pE -> 1/8,
    kAB -> 30., kBA -> 30., kAC -> 30., kCA -> 30., kAD -> 30., kDA -> 30.,
    kAE -> 30., kEA -> 30., kBC -> 30., kCB -> 30., kBD -> 30., kDB -> 30.,
    kBE -> 30., kEB -> 30., kCD -> 30., kDC -> 30., kCE -> 30., kEC -> 30., kDE -> 30., kED -> 30.,
    RA -> 30., RB -> 30., RC -> 30., RD -> 30., RE -> 30.};
absorptionSpectrum = Re[absorptionSpectrum]; *)
```

## S2 Real (absorption) part of analytical solutions

The real part (corresponding to the absorption spectrum) of the complex form of spectrum is required for spectral lineshape fitting. This section lists the analytical forms of fitting formulae for two- and three-state chemical exchange.

Experimental data are usually given in chemical shift $\delta$ in the units of ppm, defined as $\delta = ((\omega - \omega_{\mathrm{ref}})/\omega_{\mathrm{ref}}) \times 10^6$. Here $\omega_{\mathrm{ref}}$ is Larmor frequency of a standard, or alternatively $\omega_{\mathrm{ref}} = 2\pi\nu_0$, where $\nu_0$ is the spectrometer frequency. In order to convert the difference of angular frequency in rad.s$^{-1}$ to ppm, the definition of $\delta$ implies $\Delta\omega\,[\mathrm{rad.s}^{-1}] = \Delta\delta\,[\mathrm{ppm}] \times 2\pi \times \nu_0[\mathrm{MHz}]$.

A solution of both two- and three-state exchange, Eqs. (7) and (13), can be written formally as

$$S(\omega) = M_0 \frac{\mathcal{A} + i\mathcal{B}}{\mathcal{C} + i\mathcal{D}} = M_0 \left( \frac{\mathcal{AC} + \mathcal{BD}}{\mathcal{C}^2 + \mathcal{D}^2} + i \frac{\mathcal{BC} - \mathcal{AD}}{\mathcal{C}^2 + \mathcal{D}^2} \right). \tag{S8}$$

The coefficients from Eq. (S8) are summarized in the following list for both two- and three-state exchange.

**asymmetric two-state exchange**
$(k_{AB} \neq k_{BA},\, M_0^A \neq M_0^B,\, M_0 = M_0^A + M_0^B)$

$$\begin{aligned}
\mathcal{A} &= k_{AB} + k_{BA} + p_A R_2^B + p_B R_2^A \\
\mathcal{B} &= p_A(\omega - \omega_B) + p_B(\omega - \omega_A) \\
\mathcal{C} &= k_{AB} R_2^B + k_{BA} R_2^A + R_2^A R_2^B - (\omega - \omega_A)(\omega - \omega_B) \\
\mathcal{D} &= (k_{AB} + R_2^A)(\omega - \omega_B) + (k_{BA} + R_2^B)(\omega - \omega_A)
\end{aligned} \tag{S9}$$

**symmetric two-state exchange**
$(k_{AB} = k_{BA} = k,\, M_0^A = M_0^B = M_0/2)$

$$\begin{aligned}
\mathcal{A} &= (4k + R_2^A + R_2^B)/2 \\
\mathcal{B} &= (2\omega - \omega_A - \omega_B)/2 \\
\mathcal{C} &= k(R_2^A + R_2^B) + R_2^A R_2^B - (\omega - \omega_A)(\omega - \omega_B) \\
\mathcal{D} &= (k + R_2^A)(\omega - \omega_B) + (k + R_2^B)(\omega - \omega_A)
\end{aligned} \tag{S10}$$

**three-state exchange**
$(M_0 = M_0^A + M_0^B + M_0^C)$

$$\begin{aligned}
\mathcal{A} = {} & p_A[R_2^B R_2^C + R_2^B(k_{CA} + k_{CB} + k_{AC}) + R_2^C(k_{BA} + k_{BC} + k_{AB}) - (\omega - \omega_B)(\omega - \omega_C)] \\
& + p_B[R_2^A R_2^C + R_2^A(k_{CA} + k_{CB} + k_{BC}) + R_2^C(k_{AB} + k_{AC} + k_{BA}) - (\omega - \omega_A)(\omega - \omega_C)] \\
& + p_C[R_2^A R_2^B + R_2^A(k_{BA} + k_{BC} + k_{CB}) + R_2^B(k_{AB} + k_{AC} + k_{CA}) - (\omega - \omega_A)(\omega - \omega_B)] \\
& + \pi_A + \pi_B + \pi_C \\
\mathcal{B} = {} & p_A[(R_2^C + k_{CA} + k_{CB} + k_{AC})(\omega - \omega_B) + (R_2^B + k_{BA} + k_{BC} + k_{AB})(\omega - \omega_C)] \\
& + p_B[(R_2^C + k_{CA} + k_{CB} + k_{BC})(\omega - \omega_A) + (R_2^A + k_{AB} + k_{AC} + k_{BA})(\omega - \omega_C)] \\
& + p_C[(R_2^B + k_{BA} + k_{BC} + k_{CB})(\omega - \omega_A) + (R_2^A + k_{AB} + k_{AC} + k_{CA})(\omega - \omega_B)] \\
\mathcal{C} = {} & R_2^A R_2^B R_2^C + (k_{CA} + k_{CB}) R_2^A R_2^B + (k_{BA} + k_{BC}) R_2^A R_2^C + (k_{AB} + k_{AC}) R_2^B R_2^C \\
& - (R_2^C + k_{CA} + k_{CB})(\omega - \omega_A)(\omega - \omega_B) \\
& - (R_2^B + k_{BA} + k_{BC})(\omega - \omega_A)(\omega - \omega_C) \\
& - (R_2^A + k_{AB} + k_{AC})(\omega - \omega_B)(\omega - \omega_C) \\
& + R_2^A \pi_A + R_2^B \pi_B + R_2^C \pi_C \\
\mathcal{D} = {} & (R_2^B R_2^C + (k_{CA} + k_{CB}) R_2^B + (k_{BA} + k_{BC}) R_2^C + \pi_A)(\omega - \omega_A) \\
& + (R_2^A R_2^C + (k_{CA} + k_{CB}) R_2^A + (k_{AB} + k_{AC}) R_2^C + \pi_B)(\omega - \omega_B) \\
& + (R_2^A R_2^B + (k_{BA} + k_{BC}) R_2^A + (k_{AB} + k_{AC}) R_2^B + \pi_C)(\omega - \omega_C) \\
& - (\omega - \omega_A)(\omega - \omega_B)(\omega - \omega_C)
\end{aligned} \tag{S11}$$

## S3 *MATLAB* codes for exchange lineshapes

*MATLAB* codes generating the two- and three-state lineshapes discussed in this work are given below. Three methods for the calculation of the absorption lineshape are listed here: (i) calculation of complex lineshape (Eq. (7) and (13)), where only the real (absorption) part is then calculated numerically, (ii) direct calculation of the real part as in Sec. S2, and (iii) numerical solution of Eq. (S6). The numerical solution requires the calculation of an inverse matrix '`Binv`' for every given value of $\omega$. Here, we present a two-state exchange illustration using the well-known formula for 2×2 matrix inversion. The most straightforward implementation includes a '`for`' loop over all $\omega$ values. However, this implementation is too slow, therefore, we have supplied also a vectorized alternative. Another way to overcome the '`for`' loop is by conducting the numerical calculation in the time domain with consequent numerical FT.[S10]

Before calling the functions, conversion from ppm to rad.s$^{-1}$ is necessary (CODE 1). The 1D array '`delta`' contains values for the $x$-axis, e.g., `delta=0:0.01:3;`. Be aware that the conversion in CODE 1 is true only for differences of angular frequencies (e.g., for $\omega - \omega_j = 2\pi\nu_0(\delta - \delta_j)$, $j = A, B, C$). Quantities $\gamma_j$ (`gammaA`, ...) stand for the full width at half maximum (FWHM; in ppm) of non-exchanging resonances (i.e., when transition rate coefficients tend to 0), for details see discussion at the end of Sec. S5.1. The letter '`i`' here denotes the complex unit as interpreted in *MATLAB*.

Note that populations are calculated internally inside these functions (CODE 2–8) according to Eq. (6) or (15). Thus, only equilibrium kinetics in the two-state exchange and equilibrium or steady-state kinetics in the three-state exchange can be modeled.

```
% CODE 1
% conversion from ppm to rad/s
nu0 = 500.13; % 500.13 MHz spectrometer
omega = 2*pi*nu0*delta; % correct for omega-omega_j
omegaA = 2*pi*nu0*deltaA;
omegaB = 2*pi*nu0*deltaB;
% omegaC = 2*pi*nu0*deltaC; % three-state exchange
RA = 2*pi*nu0*gammaA;
RB = 2*pi*nu0*gammaB;
% RC = pi*nu0*sigmaC; % three-state exchange
```

```
% CODE 2
% lineshape for two-state exchange, complex analytical solution
function S = two_states_complex(omega,omegaA,omegaB,M0,kAB,kBA,RA,RB)
pA = kBA/(kAB+kBA);
pB = 1 - pA;
alphaA = RA + i*(omega - omegaA);
alphaB = RB + i*(omega - omegaB);

S = M0*(pA*alphaB + pB*alphaA + kAB + kBA)./ ...
  (alphaA.*alphaB + kAB*alphaB + kBA*alphaA);
S = real(S); % extract absorption part only
end
```

```
% CODE 3
% lineshape for two-state exchange, real part of analytical solution
function S=two_states_real(omega,omegaA,omegaB,M0,kAB,kBA,RA,RB)
pA = kBA/(kAB+kBA);
pB = 1 - pA;
A = kAB + kBA + pA*RB + pB*RA;
B = pA*(omega - omegaB) + pB*(omega - omegaA);
C = kAB*RB + kBA*RA + RA*RB - (omega - omegaA).*(omega - omegaB);
D = (kAB + RA)*(omega - omegaB) + (kBA + RB)*(omega - omegaA);

S = M0*(A.*C + B.*D)./(C.^2 + D.^2);
end
```

```
% CODE 4
% lineshape for symmetric two-state exchange, real part of anal. solution
function S = two_states_sym_real(omega,omegaA,omegaB,M0,k,RA,RB)
A = (4*k + RA + RB)/2;
B = (2*omega - omegaA - omegaB)/2;
C = k*(RA + RB) + RA*RB - (omega - omegaA).*(omega - omegaB);
D = (k + RA)*(omega - omegaB) + (k + RB)*(omega - omegaA);

S = M0*(A.*C + B.*D)./(C.^2 + D.^2);
end


% CODE 5
% lineshape for three-state exchange, complex analytical solution
function S = three_states_complex(omega,omegaA,omegaB,omegaC, ...
    M0,kAB,kBA,kAC,kCA,kBC,kCB,RA,RB,RC)

piA = kBA*kCA + kBC*kCA + kBA*kCB;
piB = kAB*kCA + kAB*kCB + kAC*kCB;
piC = kAC*kBA + kAB*kBC + kAC*kBC;
pA = piA/(piA + piB + piC);
pB = piB/(piA + piB + piC);
pC = piC/(piA + piB + piC);
alphaA = RA + i*(omega - omegaA);
alphaB = RB + i*(omega - omegaB);
alphaC = RC + i*(omega - omegaC);

numerator = ...
    pA*(alphaB.*alphaC + alphaB*(kCA + kCB + kAC) + alphaC*(kBA + kBC + kAB)) ...
  + pB*(alphaA.*alphaC + alphaA*(kCA + kCB + kBC) + alphaC*(kAB + kAC + kBA)) ...
  + pC*(alphaA.*alphaB + alphaA*(kBA + kBC + kCB) + alphaB*(kAB + kAC + kCA)) ...
  + piA + piB + piC;
denominator = alphaA.*alphaB.*alphaC ...
  + alphaA.*alphaB.*(kCA + kCB) + alphaA.*alphaC.*(kBA + kBC) + alphaB.*alphaC.*(kAB + kAC) ...
  + alphaA*piA + alphaB*piB + alphaC.*piC;

S = M0*numerator./denominator;
S = real(S); % extract absorption part only
end


% CODE 6
% lineshape for three-state exchange, real part of analytical solution
function S = three_states_real(omega,omegaA,omegaB,omegaC, ...
    M0,kAB,kBA,kAC,kCA,kBC,kCB,RA,RB,RC)
piA = kBA*kCA + kBC*kCA + kBA*kCB;
piB = kAB*kCA + kAB*kCB + kAC*kCB;
piC = kAC*kBA + kAB*kBC + kAC*kBC;
pA = piA/(piA + piB + piC);
pB = piB/(piA + piB + piC);
pC = piC/(piA + piB + piC);

A = pA*(RB*RC + RB*(kAC + kCA + kCB) + RC*(kBA + kBC + kAB) ...
  - (omega - omegaB).*(omega - omegaC)) ...
  + pB*(RA*RC + RA*(kCA + kCB + kBC) + RC*(kAB + kAC + kBA) ...
  - (omega - omegaA).*(omega - omegaC)) ...
  + pC*(RA*RB + RA*(kBA + kBC + kCB) + RB*(kAB + kAC + kCA) ...
  - (omega - omegaA).*(omega - omegaB)) ...
  + piA + piB + piC;
B = pA*((RC + kCA + kCB + kAC)*(omega - omegaB) ...
```

```
  + (RB + kBA + kBC + kAB)*(omega - omegaC)) ...
  + pB*((RC + kCA + kCB + kBC)*(omega - omegaA) ...
  + (RA + kAB + kAC + kBA)*(omega - omegaC)) ...
  + pC*((RB + kBA + kBC + kCB)*(omega - omegaA) ...
  + (RA + kAB + kAC + kCA)*(omega - omegaB));
C = RA*RB*RC + (kCA + kCB)*RA*RB + (kBA + kBC)*RA*RC + (kAB + kAC)*RB*RC ...
  - (RC + kCA + kCB)*(omega - omegaA).*(omega - omegaB) ...
  - (RB + kBA + kBC)*(omega - omegaA).*(omega - omegaC) ...
  - (RA + kAB + kAC)*(omega - omegaB).*(omega - omegaC) ...
  + RA*piA + RB*piB + RC*piC;
D = (RB*RC + (kCA + kCB)*RB + (kBA + kBC)*RC + piA)*(omega - omegaA) ...
  + (RA*RC + (kCA + kCB)*RA + (kAB + kAC)*RC + piB)*(omega - omegaB) ...
  + (RA*RB + (kBA + kBC)*RA + (kAB + kAC)*RB + piC)*(omega - omegaC) ...
  - (omega - omegaA).*(omega - omegaB).*(omega - omegaC);

S = M0*(A.*C + B.*D)./(C.^2 + D.^2);
end


% CODE 7
% lineshape for two-state exchange, numerical solution with for loop
function S = two_states_numeric_loop(omega,omegaA,omegaB,M0,kAB,kBA,RA,RB)
pA = kBA/(kAB + kBA);
pB = 1 - pA;
L = diag([omegaA,omegaB]);
R = diag([RA,RB]);
K = [-kAB,kBA;kAB,-kBA];

S = zeros(size(omega)); % preallocation
for idx = 1:length(omega)
    B = i*(omega(idx)*eye(2) - L) + R - K;
    Binv = 1/det(B)*[B(2,2),-B(1,2);-B(2,1),B(1,1)]; % symbolic matrix inversion
    % Binv = inv(B); % alternative calculation: numerical matrix inversion
    S(idx) = sum(M0*Binv*[pA;pB]);
end
S = real(S);
end


% CODE 8
% lineshape for two-state exchange, vectorized numerical solution
function S = two_states_numeric_vectorized(omega,omegaA,omegaB, ...
    M0,kAB,kBA,RA,RB)
pA = kBA/(kAB+kBA);
pB = 1 - pA;
L = diag([omegaA,omegaB]);
R = diag([RA,RB]);
K = [-kAB,kBA;kAB,-kBA];

W = zeros(2,2,length(omega)); % preallocation
W(1,1,:) = omega;
W(2,2,:) = omega;
B = i*(W - L) + R - K;
Binv = 1./(B(1,1,:).*B(2,2,:)-B(1,2,:).*B(2,1,:)).* ...
       [B(2,2,:),-B(1,2,:);-B(2,1,:),B(1,1,:)]; % symbolic matrix inversion
% Binv = inv(B); % alternative calculation: numerical matrix inversion

S = M0*sum(Binv.*[pA,pB],[1,2]);
S = squeeze(S); % drop empty dimensions
S = real(S);
end
```

The code for numerical calculation (CODE 7, 8) can be extended to larger numbers of states by adding more parameters to the function arguments, adjusting the kinetic matrix '`K`' and replacing the formula for inverse matrix '`Binv`'. The numerical solution can be significantly sped up by avoiding '`for`' loop using vectorization of the code. However, the vectorized code is not readily convertible to other programming languages and is less comprehensible.

In *MATLAB*, the direct calculation of the real part (CODE 3, 4, 6) is the fastest procedure. The complex calculations (CODE 2, 5) are slightly slower. The vectorized numerical implementation (CODE 8) is slower than previous methods. The numerical solution without vectorization (CODE 7) is the slowest way of computing the absorption spectrum.

## S4 Spectral lineshape in limit cases

A single Lorentzian peak in complex form is described by $S(\omega) = M_0/\alpha_j$, where $\alpha_j = R_2^j + i(\omega - \omega_j)$. Using this formula, we can easily show how the two-state expression (Eq. (7)) behaves in limit cases. The infinitely slow limit gives

$$\lim_{k_{AB},k_{BA}\to 0} S_{\text{two-state exch.}}(\omega) = M_0\left(\frac{p_A}{\alpha_A} + \frac{p_B}{\alpha_B}\right), \tag{S12}$$

which is, as expected, sum of two Lorentzian peaks.

The limit to infinity should be done in equilibrium regime, i.e., while keeping the ratio of the transition rate coefficients constant and equal to the ratio of populations, which yields

$$\lim_{k_{AB},k_{BA}\to\infty} S_{\text{two-state exch.}}(\omega)\left[\frac{k_{AB}}{k_{BA}} = \frac{p_B}{p_A}\right] = \frac{M_0}{p_A\alpha_A + p_B\alpha_B}. \tag{S13}$$

This expression is a single Lorentzian peak located at averaged frequency $p_A\omega_A + p_B\omega_B$ with averaged relaxation rate $p_A R_2^A + p_B R_2^B$.

Limits for larger numbers of states can be made analogously. For example, in the half-symmetric three-state exchange (see Sec. "Kinetics of three-state exchange"), the fast exchanging state $C$ ($k_{AC}$ and $k_{CA}$ tend to $\infty$, while their ratio is kept constant) causes vanishing of $k_A$ from the final expression, although it has a finite value (states $A_1$ and $A_2$ are in fast mutual exchange through the state $C$). Thus, the result is a single fully averaged Lorentzian peak,

$$\lim_{k_{AC},k_{CA}\to\infty} S_{\text{half-sym.three-state exch.}}(\omega)\left[\frac{k_{AC}}{k_{CA}} = \frac{p_C}{p_{A_{1,2}}}\right] = \frac{M_0}{p_{A_1}\alpha_{A_1} + p_{A_2}\alpha_{A_2} + p_C\alpha_C}. \tag{S14}$$

## S5 Coalescence

This section discusses coalescence conditions only for the two-state exchange since higher-state cases are too complicated for exploration and do not yield simple or practically recognizable coalescence patterns.

At the coalescence point, the lineshape has a distinct character (the two maxima fuse to one), which can be easily identified in the experiments without using any fitting procedure. Analytical expressions given in this section connect the value of coalescence transition rate coefficient(s) ($k_c$ in symmetric, or $k_{AB,c}$ and $k_{BA,c}$ in asymmetric exchange) with chemical shift difference of two resonances at no exchange ($\Delta\omega_{AB}$) and transversal relaxation rate at no exchange ($R_2$). This allows for rapid[S12,S13] and more accurate estimation of the transition rate coefficients at the coalescence point.

### S5.1 Expressions for coalescence in symmetric two-state exchange

We consider a completely symmetric two-state exchange, where $k_{AB} = k_{BA} = k$ and $R_2^A = R_2^B = R_2$. In the absorption lineshape, local minimum at $\omega = (\omega_A + \omega_B)/2$ (between the two peaks) vanishes with increasing $k$ value, and subsequently one new peak emerges, also at $\omega = (\omega_A + \omega_B)/2$. At this angular frequency, the lineshape changes from convex to concave and the second derivative of the real absorption part of the spectrum given by Eqs. (S8) and (S10) is zero. Therefore, the sufficient condition for coalescence of symmetric two-state exchange is[S3]

$$\frac{\mathrm{d}^2\mathrm{Re}(S_{\text{two-state, sym.}}(\omega))}{\mathrm{d}\omega^2}\Big|_{\omega=\frac{\omega_A+\omega_B}{2}} = 0\,. \tag{S15}$$

This yields a cubic equation for the coalescence transition rate coefficient $k_c$ in the form

$$4(2k_c + R_2)^3 - (4k_c + 3R_2)\Delta\omega_{AB}^2 = 0\,, \tag{S16}$$

with following exact analytical solution (introduced by Viète)

$$\begin{aligned} k_c &= \frac{\Delta\omega_{AB}}{2\sqrt{2}}\left[\frac{2}{\sqrt{3}}\cos\left(\frac{1}{3}\arccos\left(\frac{3\sqrt{3}}{2\sqrt{2}}\frac{R_2}{\Delta\omega_{AB}}\right)\right) - \sqrt{2}\frac{R_2}{\Delta\omega_{AB}}\right];\ 0 \le \frac{R_2}{\Delta\omega_{AB}} \le \frac{2\sqrt{2}}{3\sqrt{3}}\,,\\ k_c &= \frac{\Delta\omega_{AB}}{2\sqrt{2}}\left[\frac{2}{\sqrt{3}}\cosh\left(\frac{1}{3}\mathrm{arccosh}\left(\frac{3\sqrt{3}}{2\sqrt{2}}\frac{R_2}{\Delta\omega_{AB}}\right)\right) - \sqrt{2}\frac{R_2}{\Delta\omega_{AB}}\right];\ \frac{2\sqrt{2}}{3\sqrt{3}} < \frac{R_2}{\Delta\omega_{AB}} \le \frac{\sqrt{3}}{2}\,, \end{aligned} \tag{S17}$$

where $\Delta\omega_{AB} = |\omega_A - \omega_B|$. Note that there is a maximum value of $R_2/\Delta\omega_{AB} = \sqrt{3}/2$ at which the resonances coalesce in the absence of exchange ($k_c = 0\ \mathrm{s}^{-1}$). This is due to the extreme broadness of the individual resonances due to the high intrinsic value of the transverse relaxation rate $R_2$. There is an experimentally practical range of $R_2/\Delta\omega_{AB} \in [0, 0.5]$, where the information can be reliably obtained from spectra. After setting $R_2 = 0$ in Eq. (S17) the expressions in square brackets are equal to unity, and we obtain the well-known (zero-order) expression for transition rate coefficient at coalescence,[S3]

$$k_c = \frac{\Delta\omega_{AB}}{2\sqrt{2}}\,. \tag{S18}$$

The formula (S17) can be approximated as a series in powers of $R/\Delta\omega_{AB}$,

$$k_c \approx \frac{\Delta\omega_{AB}}{2\sqrt{2}}\left[1 + a_1\frac{R_2}{\Delta\omega_{AB}} + a_2\left(\frac{R_2}{\Delta\omega_{AB}}\right)^2 + a_3\left(\frac{R_2}{\Delta\omega_{AB}}\right)^3\right]. \tag{S19}$$

Values of coefficients $a_i$ for different orders of approximation are listed in the following Table S1.

**Table S1.** Coefficients in $n$-th order polynomial approximation of the coalescence point in Eq. (S19) for symmetric two-state exchange lineshape with $R_2^A = R_2^B \neq 0$.

| | $a_1$ | $a_2$ | $a_3$ |
|---|---|---|---|
| 0th order | 0 | 0 | 0 |
| 1st order | −1.1379 | 0 | 0 |
| 2nd order | −1.0805 | $-8.8062\times10^{-2}$ | 0 |
| 3rd order | −1.0659 | $-1.4392\times10^{-1}$ | $4.8246\times10^{-2}$ |

All the coefficients $a_i$ were obtained by least-squares fitting of the polynomial to the exact solution in Eq. (S17) while fixing the zero-order term in square brackets in Eq. (S19) to 1 (to maintain correspondence with the well-known formula $k_c = \Delta\omega_{AB}/(2\sqrt{2})$ when $R_2 = 0$).

We have also explored another type of approximation of Eq. (S17), the so-called Padé approximant.[S14] The Padé approximant of type $[m/n]$ is a rational function with a numerator of order up to $m$ and a denominator of order up to $n$. The Padé approximant of the order [2/2] at $R_2/\Delta\omega_{AB} = 0$ for Eq. (S17) yields the following expression for the transition rate coefficient at coalescence

$$k_c \approx \frac{\Delta\omega_{AB}}{2\sqrt{2}} \frac{1824 + 84\sqrt{2}\frac{R_2}{\Delta\omega_{AB}} - 2579(\frac{R_2}{\Delta\omega_{AB}})^2}{1824 + 1452\sqrt{2}\frac{R_2}{\Delta\omega_{AB}} - 59(\frac{R_2}{\Delta\omega_{AB}})^2}. \tag{S20}$$

The quality of the approximation is illustrated in Fig. S1. Note that the relative error does not depend on $\Delta\omega_{AB}$ alone but on the ratio $R_2/\Delta\omega_{AB}$. It can be seen that the first-order (in $R_2/\Delta\omega_{AB}$) approximation (Fig. S1, blue line) already significantly reduces the relative error of $k_c$ (rel. err. up to ca. 1.6 %) for a very broad region of experimentally relevant $R_2/\Delta\omega_{AB}$ values (i.e., $R_2/\Delta\omega_{AB} \in [0, 0.5]$). The zero-order approximation generates extreme relative errors for already small and experimentally most relevant $R_2/\Delta\omega_{AB}$ values (rel. err. up to ca. 130 % in $R_2/\Delta\omega_{AB} \in [0, 0.5]$, Fig. S1, black line).

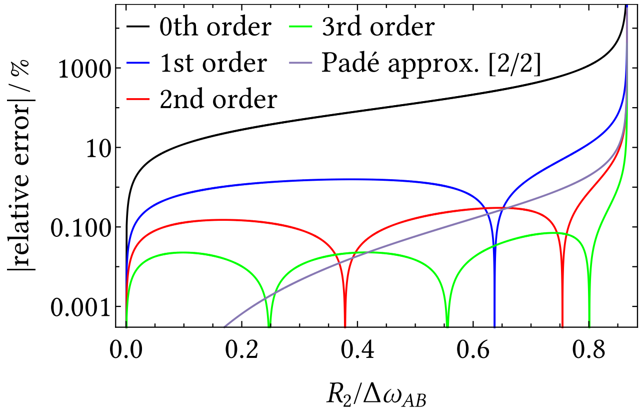


**Figure S1.** Quality of various approximations of transition rate coefficient $k_c$ at coalescence of two-state symmetric exchange with $R_2 > 0$ as expressed in Eqs. (S19) and (S20). The relative error is calculated as $|k_c^{\mathrm{approx}} - k_c^{\mathrm{exact}}|/(k_c^{\mathrm{exact}}) \times 100\%$, where $k_c^{\mathrm{approx}}$ are obtained from approximate formulae and $k_c^{\mathrm{exact}}$ is obtained from exact solution in Eq. (S17).

We also briefly discuss several practical points for rapid utilisation of approximative formulae for $k_c$, since they contain relaxation rate $R_2$, an extra variable that needs to be estimated. Values of $R_2$ and $\Delta\omega_{AB}$ can be obtained from a spectrum with no exchange present (i.e., in the limit $k \to 0$ s$^{-1}$), usually obtained at low temperature or in the absence of an exchange triggering factor such as acid molecules (this situation is extensively discussed in other parts of the manuscript). For convenience, we extract the spectral data in ppm units and use the general conversion relation for the frequency differences $\Delta\omega = 2\pi\nu_0\Delta\delta$, where $\nu_0$ is spectrometer frequency in MHz. The transverse relaxation rate $R_2$ (in s$^{-1}$) can be estimated from full-width at half maximum, which we conveniently denote as $2\gamma$ (in ppm), and then the conversion is $R_2 = 2\pi\nu_0\gamma$. This approach is feasible when the magnetic field inhomogeneities, which contribute to broadening, are small (this is usually the case for spectra obtained from properly shimmed spectrometer). The $\Delta\omega_{AB}$ (in rad.s$^{-1}$) is determined (at $k \to 0$ s$^{-1}$) from the difference between $A$ and $B$ state peak maxima $\Delta\delta_{AB} = |\delta_A - \delta_B|$ (in ppm) as $\Delta\omega_{AB} = 2\pi\nu_0\Delta\delta_{AB}$. The ratio $R_2/\Delta\omega_{AB}$ used in the approximation formulae can then be conveniently evaluated as $R_2/\Delta\omega_{AB} = \gamma/\Delta\delta_{AB}$.

In Table S2, we give a model example of the coalescence transition rate coefficient estimation and associated relative errors for spectrometer with operating frequency $\nu_0 = 400$ MHz. For given $\Delta\delta_{AB} = 0.1$ ppm and $2\gamma = 0.023$ ppm the $\Delta\omega_{AB} = 251.33$ rad.s$^{-1}$ and $R/\Delta\omega_{AB} = 0.115$.

It can be seen that the Padé approximant yields excellent accuracy, but the formula is rather impractical for routine use; the 1$^{\mathrm{st}}$ order approximation has a simple formula and reasonably high accuracy for most practical purposes.

### S5.2 Coalescence in asymmetric two-state exchange

In the case of asymmetric two-state exchange with lineshape given by Eqs. (S8) and (S9), the situation at coalescence is more complex. The coalescence condition from the symmetric case, Eq. (S15), cannot be used because the resulting merged peak after coalescence is not, in general, positioned at $(\omega_A + \omega_B)/2$. In addition, the important region of lineshape (i.e., between the peaks) does not necessarily change from convex to concave when the two resonances coalesce during variation of transition rate coefficients. In general, at coalescence an inflexion point is created. This is in contrast to the symmetric case where a convex-to-concave change is always expected. Therefore, we have introduced a generalized definition for coalescence in the

**Table S2.** Accuracy of $n$-th order polynomial approximation of the coalescence point in Eq. (S19) for symmetric two-state exchange lineshape with $R_2^A = R_2^B \neq 0$. The relative error is calculated as $|k_c^{\text{approx}} - k_c^{\text{exact}}|/(k_c^{\text{exact}}) \times 100\%$.

| | Value [$s^{-1}$] | Rel. error |
|---|---|---|
| $k_c^{\text{exact}}$ | 77.82 | |
| $k_c^{\text{0th-order}}$ | 88.86 | 14.2 % |
| $k_c^{\text{1st-order}}$ | 77.23 | 0.76 % |
| $k_c^{\text{2nd-order}}$ | 77.71 | 0.14 % |
| $k_c^{\text{3rd-order}}$ | 77.80 | 0.02 % |
| $k_c^{\text{Padé}}$ | 77.82 | 4.6×10$^{-5}$ % |

asymmetric exchange mode as *a point of change in the number of peak maxima*. In asymmetric two-state exchange, coalescence occurs when one local maximum and one local minimum merge into one inflexion point (while the other local maximum remains). This situation can be seen in Fig. S15a, where an increase of $k_{BA}$ value causes coalescence of the $\delta_A$ peak at around 0.9 equiv. Hence, coalescence occurs at the point $\{k_{AB,c}, k_{BA,c}\}$ where the number of stationary points (i.e., maxima, minima and inflexion points) changes from two to three (or vice versa). In terms of calculus, the stationary points of the absorption lineshape $\mathrm{Re}(S_{\text{two-state,asym.}}(\omega))$ (Eqs. (S8) and (S9)) are determined by setting the first derivative of the lineshape to zero, formally

$$\frac{\mathrm{d}\,\mathrm{Re}(S_{\text{two-state,asym.}}(\omega))}{\mathrm{d}\omega} = 0\,. \tag{S21}$$

The number of stationary points is given by the number of real solutions of Eq. (S21). Since this equation is a polynomial equation, a variation of the number of real solutions occurs when the *discriminant of Eq. (S21) with respect to $\omega$ is zero*, which mathematically reflects our generalized condition for coalescence.

Now, let us consider $R_2 = 0$ situation. This yields the following cubic equation in $\omega$

$$k_{AB}^2(\omega-\omega_B) + k_{BA}^2(\omega-\omega_A) + k_{AB}k_{BA}\left[(\omega-\omega_A)+(\omega-\omega_A)\right] + (\omega-\omega_A)^2(\omega-\omega_B) + (\omega-\omega_A)(\omega-\omega_B)^2 = 0\,. \tag{S22}$$

For the $R_2 = 0$ case, setting the discriminant (with respect to $\omega$) of Eq. (S22) to zero eliminates the variable $\omega$ and gives a polynomial equation of sixth order in $k_{AB}$ and $k_{BA}$ with dozens of terms (not shown here). Where $R_2^A = R_2^B = R_2 > 0$, Eq. (S21) yields a quintic equation in $\omega$, which also contains dozens of terms (not shown here). Again setting its discriminant (with respect to $\omega$) to zero leads to an eighth order polynomial equation in both $k_{AB}$ and $k_{BA}$ with approximately twenty thousand terms. Both cases ($R_2 = 0$, $R_2 > 0$) are tractable by the *Mathematica* software by a combination of symbolic and numerical approaches.

Unlike the symmetric two-state exchange[S15] where the coalescence transition rate coefficient has only one value of $k_c$ (at fixed $\Delta\omega_{AB}$ and $R_2$), the asymmetric exchange exhibits infinitely many $\{k_{AB,c}, k_{BA,c}\}$ pairs at which the spectrum has coalescence lineshape, see Fig. S2a. In this graph, we have selected several representative situations (three values of $\Delta\omega_{AB}$ and two values of $R_2$) and analyzed the coalescence behavior dependent on $k_{AB}$ and $k_{BA}$. This figure represents a sort of "$k_{AB}$–$k_{BA}$ phase diagram" describing qualitatively the spectral lineshapes in asymmetric exchange.

It would be convenient for asymmetric exchange to have a formula (similar to symmetric exchange case) that predicts both transition rate coefficients at coalescence $k_{AB,c}$ and $k_{BA,c}$. Unfortunately, the complex nature of asymmetric exchange does not permit the derivation of any reasonable analytical formulae for $k_{AB,c}$ and $k_{BA,c}$. However, some rough observations can be made based on the results shown in Fig. S2a. Note that the curves of coalescence in this figure form approximately square shapes, hence, the approximate conditions for coalescence (i.e., change in the number of local extrema of spectral lineshape from three to just one) can be estimated as $k_{AB} \approx 0.9|\omega_B - \omega_A|/(2\sqrt{2})$ or $k_{BA} \approx 0.9|\omega_B - \omega_A|/(2\sqrt{2})$ (formulae analogous to the symmetric case with 0.9 as a correction factor). Essentially, only one value ($k_{AB}$ or $k_{BA}$) can be determined by a naked-eye inspection of the spectrum at coalescence and this choice is based on more intense resonance, as can be seen in Fig. S2a. When the positions of $A$ and $B$ states in the spectrum are known, then if the $A$ state resonance is more intense, then $k_{BA} \approx 0.9|\omega_B - \omega_A|/(2\sqrt{2})$ (Fig. S2b: curves I, II, III); and if $B$ state resonance is more intensive, the $k_{AB} \approx 0.9|\omega_B - \omega_A|/(2\sqrt{2})$ (Fig. S2b: curve IV). Also, note that using this method, the relative error in $k_{AB}$ or $k_{BA}$ estimation is at least 15 %.

Figure S2 is constructed in line with the analysis of exchange kinetics in the 1:1 host–guest binding model in Sec. S11.2. The kinetics was studied during the simulated titration experiments (addition of guest molecule), where we observed an increase in $k_{AB}$ while the $k_{BA}$ transition rate coefficient remained unchanged (Fig. S15e). It can be illustrated in Fig. S2a by changing the $k_{AB}$ value at fixed $k_{BA}$ = 1500 s$^{-1}$ (for $\Delta\omega_{AB}$ = 4710 rad.s$^{-1}$), which yields coalescence encountered at four points I–IV. Hence, Fig. S2b focuses on one extreme case where it is possible to identify four coalescences. This is not necessarily the case for all titration experiments although it is relatively easy to tune the system by varying temperature to approach this situation.

During the analysis of kinetics by variable temperature NMR study (not a titration experiment), the coalescence line in the "$k_{AB}$–$k_{BA}$ phase diagram" (Fig. S2b) is crossed usually only once (i.e., the path in the "$k_{AB}$–$k_{BA}$ phase diagram" starts close to the origin and then increases nearly linearly with constant angle).

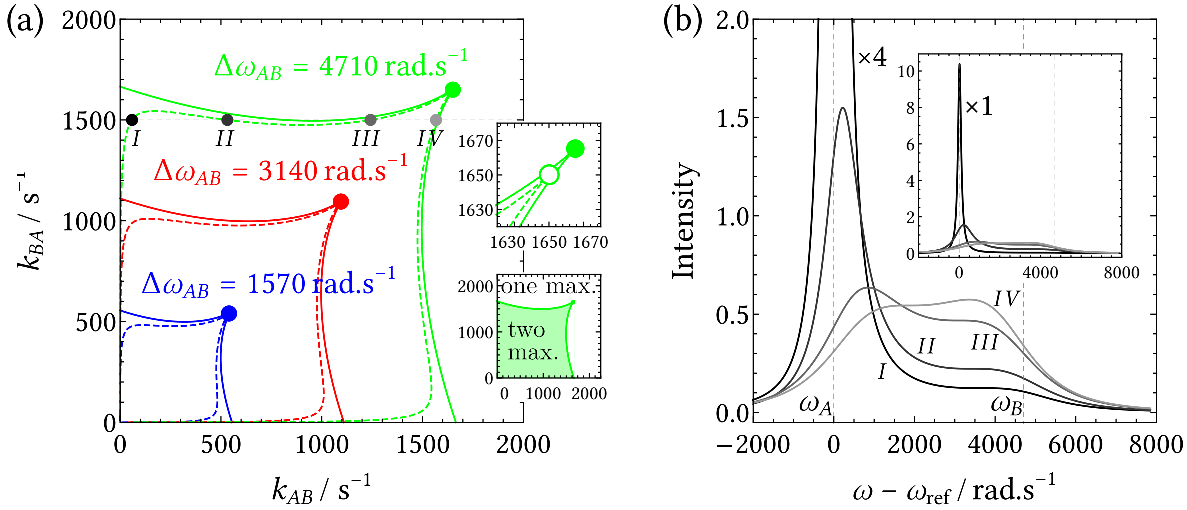


**Figure S2.** Generalized conditions for coalescence of the asymmetric two-state exchange. (**a**) Coalescence points plotted as "$k_{AB}$–$k_{BA}$ phase diagram". Values of $\Delta\omega_{AB}$ =1570, 3140 and 4710 rad.s$^{-1}$ correspond to 0.5, 1.0 and 1.5 ppm, respectively, using a 500.13 MHz spectrometer. Full lines imply coalescence for $R_2^A = R_2^B = 0$, dashed lines are coalescence for $R_2^A = R_2^B = 40$ s$^{-1}$ (0.013 ppm). Solid colored points at the diagonal position denote coalescence of symmetric exchange calculated from Eq. (S18). Symmetric coalescence for zero (solid circle) and non-zero relaxation rates (empty circle) can be distinguished only at high magnification (upper inset). The region inside the coalescence curves implies the presence of two maxima; the outside area corresponds to one maximum (illustrated in the lower inset for $\Delta\omega_{AB} = 4710$ rad.s$^{-1}$ and $R_2^A = R_2^B = 0$ s$^{-1}$). Points I–IV correspond to values $k_{AB} = 59$, 532, 1242 and 1567 s$^{-1}$ and $k_{BA} = 1500$ s$^{-1}$. (**b**) Spectra at coalescence corresponding to the points I–IV in the part (**a**) for $\Delta\omega_{AB} = 4710$ rad.s$^{-1}$ ($\omega_A$ is set to zero) and $R_2^A = R_2^B = 40$ s$^{-1}$. In the main graph, the spectrum I is magnified ×4 for clarity. The definition and meaning of the reference angular frequency $\omega_{\text{ref}}$ is explained at the beginning of Sec. S2.

## S6 Two-state exchange with two processes

### S6.1 Basic relations

The kinetic equations for a two-state system with two processes can be formulated using population fluxes defined as $J^{\text{I}}_{jk} = p_j k^{\text{I}}_{jk} - p_k k^{\text{I}}_{kj}$ (population flux from $j$ to $k$ due to process I) and $J^{\text{II}}_{jk} = p_j k^{\text{II}}_{jk} - p_k k^{\text{II}}_{kj}$ (population flux from $j$ to $k$ due to process II) as

$$\frac{\mathrm{d}p_A}{\mathrm{d}t} = J^{\text{I}}_{BA} + J^{\text{II}}_{BA}\,,$$
$$\frac{\mathrm{d}p_B}{\mathrm{d}t} = J^{\text{I}}_{AB} + J^{\text{II}}_{AB}\,. \qquad \text{(S23)}$$

Only one of these equations is independent, hence, the condition for a steady-state after setting the left-hand side to zero is $J^{\text{I}}_{BA} = -J^{\text{II}}_{BA}$ or equivalently $J^{\text{I}}_{BA} = J^{\text{II}}_{AB}$. The immediate interpretation, illustrated in Fig. S3a–c, implies that the population flux is in a clockwise or counter-clockwise sense depending on the sign of $J^{\text{I}}_{AB}$. If there are non-zero population fluxes in both branches (I and II), a steady-state is achieved. If there is no population flux (i.e., $J^{\text{I}}_{AB} = J^{\text{II}}_{BA} = 0$), the system is in equilibrium.

Comparing this type of dynamics with the simple one-process two-state exchange, the kinetic matrix $\mathbf{K}_{\text{two-proc.}}$ is constructed

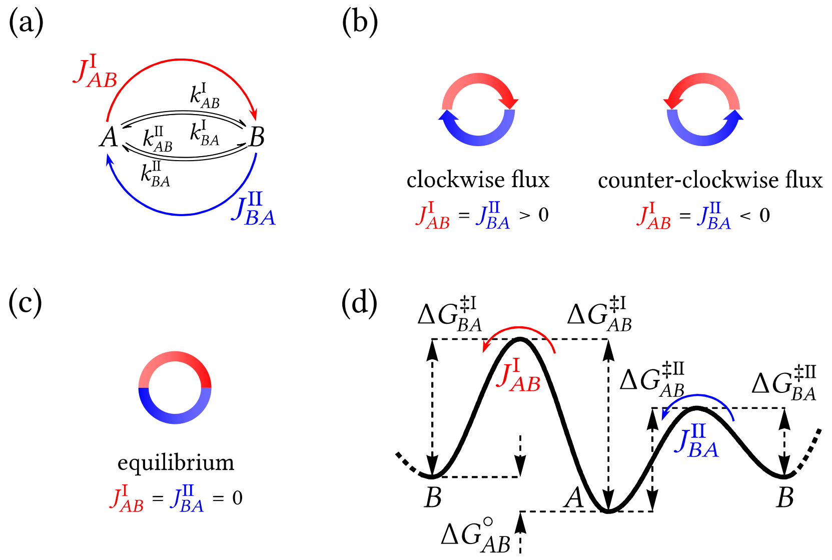


**Figure S3.** Two-state exchange with two independent processes. (**a**) Definition of population fluxes. (**b**) Steady-state population fluxes. (**c**) Equilibrium state (with no population fluxes) and its equilibrium energy landscape shown in (**d**).

by replacing $k_{AB} \to k^{\mathrm{I}}_{AB} + k^{\mathrm{II}}_{AB}$ and $k_{BA} \to k^{\mathrm{I}}_{BA} + k^{\mathrm{II}}_{BA}$ in $\mathbf{K}_{\text{one-proc.}}$. Analogously with Eq. (6) in the main manuscript, it follows

$$p^{\mathrm{ss}}_A = \frac{k^{\mathrm{I}}_{BA} + k^{\mathrm{II}}_{BA}}{k^{\mathrm{I}}_{AB} + k^{\mathrm{II}}_{AB} + k^{\mathrm{I}}_{BA} + k^{\mathrm{II}}_{BA}},$$
$$p^{\mathrm{ss}}_B = \frac{k^{\mathrm{I}}_{AB} + k^{\mathrm{II}}_{AB}}{k^{\mathrm{I}}_{AB} + k^{\mathrm{II}}_{AB} + k^{\mathrm{I}}_{BA} + k^{\mathrm{II}}_{BA}}. \tag{S24}$$

In equilibrium (not steady-state) the populations and transition rate coefficients are linked together by

$$\frac{p^{\mathrm{eq}}_A}{p^{\mathrm{eq}}_B} = \frac{k^{\mathrm{I}}_{BA}}{k^{\mathrm{I}}_{AB}} = \frac{k^{\mathrm{II}}_{BA}}{k^{\mathrm{II}}_{AB}}, \tag{S25}$$

which follows from the conditions $J^{\mathrm{I}}_{AB} = J^{\mathrm{II}}_{BA} = 0$. If the transition rate coefficients do not depend on concentrations (e.g., for a molecular rotor), they are equal to corresponding reaction rate coefficients (i.e., $k^{\mathrm{I}}_{AB} = \kappa^{\mathrm{I}}_{AB}$, $k^{\mathrm{I}}_{BA} = \kappa^{\mathrm{I}}_{BA}$ and analogously for process II). In this case, the populations can be related to the standard reaction Gibbs energy by $p^{\mathrm{eq}}_B/p^{\mathrm{eq}}_A = \exp\left(-\Delta G^{\circ}_{AB}/RT\right)$, and the chemical kinetics scheme can be visualized as Gibbs energy landscape as shown in Fig. S3d.

After substituting the equilibrium condition $p^{\mathrm{eq}}_B/p^{\mathrm{eq}}_A = k^{\mathrm{I}}_{AB}/k^{\mathrm{I}}_{BA}$ (or $p^{\mathrm{eq}}_B/p^{\mathrm{eq}}_A = k^{\mathrm{II}}_{AB}/k^{\mathrm{II}}_{BA}$) into Eq. (S24), or simply into $p^{\mathrm{eq}}_A + p^{\mathrm{eq}}_B = 1$ we obtain the following equilibrium concentrations

$$p^{\mathrm{eq}}_A = \frac{k^{\mathrm{I}}_{BA}}{k^{\mathrm{I}}_{AB} + k^{\mathrm{I}}_{BA}} = \frac{k^{\mathrm{II}}_{BA}}{k^{\mathrm{II}}_{AB} + k^{\mathrm{II}}_{BA}},$$
$$p^{\mathrm{eq}}_B = \frac{k^{\mathrm{I}}_{AB}}{k^{\mathrm{I}}_{AB} + k^{\mathrm{I}}_{BA}} = \frac{k^{\mathrm{II}}_{AB}}{k^{\mathrm{II}}_{AB} + k^{\mathrm{II}}_{BA}}. \tag{S26}$$

Note that in the case of one-process two-state exchange, the system can reach equilibrium only by changing its populations. However, in the presence of two processes between $A$ and $B$ states, not only the populations but also the transition rate coefficients must be adjusted in order to reach equilibrium. Thus, all the transition rate coefficients are interdependent at equilibrium, e.g., $k^{\mathrm{I}}_{BA} = k^{\mathrm{I}}_{AB}k^{\mathrm{II}}_{BA}/k^{\mathrm{II}}_{AB}$, as follows from Eq. (S25).

The NMR spectral lineshape of two-process two-state exchange is described by Eq. (7) using substitutions $k_{AB} \to k^{\mathrm{I}}_{AB} + k^{\mathrm{II}}_{AB}$ and $k_{BA} \to k^{\mathrm{I}}_{BA} + k^{\mathrm{II}}_{BA}$. The corresponding populations are calculated using Eq. (S24) (at steady-state) or Eq. (S26) (at equilibrium). Thus, in principle, it is impossible to infer the number of underlying processes or differentiate a steady-state from an equilibrium from a single spectral lineshape in a two-state exchange. In theory, the presence of two-process exchange can be

distinguished from the one-process exchange by a series of measurements at different temperatures since the Eyring plot should deviate from linearity. However, these deviations could be too small to be correctly identified as two processes. Therefore, the best approach is to consider all possible exchange processes from structural constraints given by the particular molecular system studied. Then the Eyring plot can be correctly analyzed and interpreted.

### S6.2 Catalyzed chemical reaction

In practice, two-process two-state exchange can be achieved, for example, in a chemical reaction where substrate **S** is transformed to product **P** by means of enzyme **E** catalysis (reversible Michaelis-Menten kinetics[S16]), see Fig. S4a(i). During this process, a nuclear spin corresponding to the enzyme undergoes exchange between free and complexed states denoted as $\mathbf{E}^A$ and $(\mathbf{ES})^B$, respectively, see Fig. S4a(ii). The chemical and spin kinetics parameters are related by expressions listed in Fig. S4a(iii). This type of kinetics can be simulated either as freely evolving system (see equations in Fig. S4b), or as a system externally forced to a steady-state by maintaining constant concentrations of free substrate $[\mathbf{S}]_0$ and product $[\mathbf{P}]_0$ (see equations in Fig. S4c).

(a) **kinetic schemes**

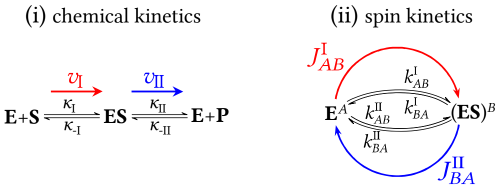


(iii) relation between chemical and spin kinetics

$$k^{\mathrm{I}}_{AB} = \kappa_{\mathrm{I}}[\mathbf{S}] \qquad J^{\mathrm{I}}_{AB} = \frac{\upsilon_{\mathrm{I}}}{[\mathbf{E}]_t}$$
$$k^{\mathrm{I}}_{BA} = \kappa_{-\mathrm{I}}$$
$$k^{\mathrm{II}}_{AB} = \kappa_{-\mathrm{II}}[\mathbf{P}] \qquad J^{\mathrm{II}}_{BA} = \frac{\upsilon_{\mathrm{II}}}{[\mathbf{E}]_t}$$
$$k^{\mathrm{II}}_{BA} = \kappa_{\mathrm{II}}$$

(b) **free evolution**

(i) governing equations

$$\upsilon_{\mathrm{I}} = \kappa_{\mathrm{I}}[\mathbf{S}][\mathbf{E}] - \kappa_{-\mathrm{I}}[\mathbf{ES}]$$
$$\upsilon_{\mathrm{II}} = \kappa_{\mathrm{II}}[\mathbf{ES}] - \kappa_{-\mathrm{II}}[\mathbf{P}][\mathbf{E}]$$
$$\frac{\mathrm{d}}{\mathrm{d}t}[\mathbf{E}] = -\upsilon_{\mathrm{I}} + \upsilon_{\mathrm{II}}$$
$$\frac{\mathrm{d}}{\mathrm{d}t}[\mathbf{P}] = \upsilon_{\mathrm{II}}$$
$$[\mathbf{E}]_t = [\mathbf{E}] + [\mathbf{ES}]$$
$$[\mathbf{S}]_t = [\mathbf{S}] + [\mathbf{ES}] + [\mathbf{P}]$$

initial conditions
$[\mathbf{S}](0) = [\mathbf{S}]_t$
$[\mathbf{P}](0) = 0$

(ii) equations for steady-state ($\upsilon_{\mathrm{I}} = \upsilon_{\mathrm{II}}$)

$$\frac{[\mathbf{E}]^{ss}}{[\mathbf{E}]_t} = \frac{\kappa_{-\mathrm{I}} + \kappa_{\mathrm{II}}}{\kappa_{-\mathrm{I}} + \kappa_{\mathrm{II}} + \kappa_{-\mathrm{II}}[\mathbf{P}] + \kappa_{\mathrm{I}}[\mathbf{S}]}$$
$$\frac{[\mathbf{ES}]^{ss}}{[\mathbf{E}]_t} = \frac{\kappa_{-\mathrm{II}}[\mathbf{P}] + \kappa_{\mathrm{I}}[\mathbf{S}]}{\kappa_{-\mathrm{I}} + \kappa_{\mathrm{II}} + \kappa_{-\mathrm{II}}[\mathbf{P}] + \kappa_{\mathrm{I}}[\mathbf{S}]}$$

(iii) equations for equilibrium ($\upsilon_{\mathrm{I}} = \upsilon_{\mathrm{II}} = 0$)

$$K_{\mathrm{I}} = \frac{\kappa_{\mathrm{I}}}{\kappa_{-\mathrm{I}}} = \frac{[\mathbf{ES}]^{eq}}{[\mathbf{E}]^{eq}[\mathbf{S}]^{eq}}$$
$$K_{\mathrm{II}} = \frac{\kappa_{-\mathrm{II}}}{\kappa_{\mathrm{II}}} = \frac{[\mathbf{ES}]^{eq}}{[\mathbf{E}]^{eq}[\mathbf{P}]^{eq}}$$
$$[\mathbf{E}]_t = [\mathbf{E}]^{eq} + [\mathbf{ES}]^{eq}$$
$$[\mathbf{S}]_t = [\mathbf{S}]^{eq} + [\mathbf{P}]^{eq} + [\mathbf{ES}]^{eq}$$

(iv) solution for equilibrium

$$\frac{[\mathbf{E}]^{eq}}{[\mathbf{E}]_t} = \frac{1}{K_{\mathrm{I}}[\mathbf{S}]^{eq} + 1} = \frac{1}{K_{\mathrm{II}}[\mathbf{P}]^{eq} + 1}$$
$$\frac{[\mathbf{ES}]^{eq}}{[\mathbf{E}]_t} = \frac{K_{\mathrm{I}}[\mathbf{S}]^{eq}}{K_{\mathrm{I}}[\mathbf{S}]^{eq} + 1} = \frac{K_{\mathrm{II}}[\mathbf{P}]^{eq}}{K_{\mathrm{II}}[\mathbf{P}]^{eq} + 1}$$

$$[\mathbf{S}]^{eq} = \frac{\sqrt{[K_{\mathrm{I}}K_{\mathrm{II}}([\mathbf{S}]_t - [\mathbf{E}]_t) + K_{\mathrm{I}} + K_{\mathrm{II}}]^2 + 4[\mathbf{E}]_t K_{\mathrm{I}}K_{\mathrm{II}}(K_{\mathrm{I}} + K_{\mathrm{II}})} - [\mathbf{E}]_t K_{\mathrm{II}}K_{\mathrm{I}} + K_{\mathrm{II}}K_{\mathrm{I}}[\mathbf{S}]_t - K_{\mathrm{I}} - K_{\mathrm{II}}}{2K_{\mathrm{I}}(K_{\mathrm{I}} + K_{\mathrm{II}})}$$
$$[\mathbf{P}]^{eq} = \frac{\sqrt{[K_{\mathrm{I}}K_{\mathrm{II}}([\mathbf{S}]_t - [\mathbf{E}]_t) + K_{\mathrm{I}} + K_{\mathrm{II}}]^2 + 4[\mathbf{E}]_t K_{\mathrm{I}}K_{\mathrm{II}}(K_{\mathrm{I}} + K_{\mathrm{II}})} - [\mathbf{E}]_t K_{\mathrm{II}}K_{\mathrm{I}} + K_{\mathrm{II}}K_{\mathrm{I}}[\mathbf{S}]_t - K_{\mathrm{I}} - K_{\mathrm{II}}}{2K_{\mathrm{II}}(K_{\mathrm{I}} + K_{\mathrm{II}})}$$

(c) **forced steady-state**

(i) governing equations

$$\upsilon_{\mathrm{I}} = \kappa_{\mathrm{I}}[\mathbf{S}]_0[\mathbf{E}] - \kappa_{-\mathrm{I}}[\mathbf{ES}]$$
$$\upsilon_{\mathrm{II}} = \kappa_{\mathrm{II}}[\mathbf{ES}] - \kappa_{-\mathrm{II}}[\mathbf{P}]_0[\mathbf{E}]$$
$$\frac{\mathrm{d}}{\mathrm{d}t}[\mathbf{E}] = -\upsilon_{\mathrm{I}} + \upsilon_{\mathrm{II}}$$
$$[\mathbf{E}]_t = [\mathbf{E}] + [\mathbf{ES}]$$

fixed concentrations
$[\mathbf{S}](t) = [\mathbf{S}]_0$ $\quad t \geq 0$
$[\mathbf{P}](t) = [\mathbf{P}]_0$

(ii) equations for steady-state ($\upsilon_{\mathrm{I}} = \upsilon_{\mathrm{II}}$)

$$[\mathbf{E}]^{ss} = \frac{[\mathbf{E}]_t(\kappa_{-\mathrm{I}} + \kappa_{\mathrm{II}})}{\kappa_{-\mathrm{I}} + \kappa_{\mathrm{II}} + \kappa_{-\mathrm{II}}[\mathbf{P}]_0 + \kappa_{\mathrm{I}}[\mathbf{S}]_0}$$
$$[\mathbf{ES}]^{ss} = \frac{[\mathbf{E}]_t(\kappa_{-\mathrm{II}}[\mathbf{P}]_0 + \kappa_{\mathrm{I}}[\mathbf{S}]_0)}{\kappa_{-\mathrm{I}} + \kappa_{\mathrm{II}} + \kappa_{-\mathrm{II}}[\mathbf{P}]_0 + \kappa_{\mathrm{I}}[\mathbf{S}]_0}$$

**Figure S4.** Enzyme-catalyzed chemical reaction (Michaelis-Menten kinetics). (**a**) Schemes for (i) chemical kinetics and (ii) corresponding spin-exchange kinetics, (iii) relation between the parameters describing both chemical and spin kinetics. Note that $\upsilon_\bullet$ stands for reaction rate in branches I or II. (**b**) Expressions describing free evolution. (i) Governing differential equations, (ii) algebraic equations for steady-state (cf. Eq. (S24) for spin kinetics) and (iii) values of equilibrium populations (at $t \to \infty$, cf. Eq. (S26) for spin kinetics). (**c**) Expressions describing externally forced steady-state. The values of $[\mathbf{S}]_0$ and $[\mathbf{P}]_0$ are fixed, which corresponds to addition of substrate and removal of product, respectively. (i) Governing differential equations and (ii) algebraic equations for steady-state.

Free evolution of the system (Fig. S4b) is governed by differential equations (utilizing reaction rates $\upsilon_{\mathrm{I}}$ and $\upsilon_{\mathrm{II}}$) listed in Fig. S4b(i). The solution does not provide the "true" steady-state, since the concentrations vary with time until they reach equilibrium values, see Fig. S5a,b,c. Figure S5d clearly shows two regimes, (i) initial *pre-steady-state*, where both reaction rates

are very different, i.e., $v_{\rm I}-v_{\rm II}\gg 0$ (the pre-steady-state phase is generally very short, in order of ms[S16]), and (ii) *approximate steady-state*, where both reaction rates are almost the same, i.e., $v_{\rm I}-v_{\rm II}\approx 0$. Steady-state assumption is frequently used in enzyme kinetics studies because it simplifies the governing equations,[S16] see Fig. S4b(ii). The steady-state relations are comprised only of two equations for $[\mathbf{E}]^{\rm ss}$ and $[\mathbf{ES}]^{\rm ss}$ (or $p_A^{\rm ss}=\frac{[\mathbf{E}]^{\rm ss}}{[\mathbf{ES}]_{\rm t}}$ and $p_B^{\rm ss}=\frac{[\mathbf{ES}]^{\rm ss}}{[\mathbf{E}]_{\rm t}}$ for the spin kinetics), both depending on $[\mathbf{S}]$ and $[\mathbf{P}]$. These concentrations should be obtained experimentally in order to calculate the steady-state concentrations of **E** and **ES** (e.g., by NMR peak integration or from UV-vis absorbance values). Comparing the steady-state approximation (green lines in Fig. S5a,b) with exactly calculated dynamics (i.e., numerical solution of governing equations in Fig. S4b(i); purple and blue lines in Fig. S5a,b) an excellent agreement is obtained after the *pre-steady-state* phase. Even when the "true" steady-state is not achieved, during a short time period the concentrations stay approximately constant, since $\frac{{\rm d}[\mathbf{ES}]}{{\rm d}t}=-\frac{{\rm d}[\mathbf{E}]}{{\rm d}t}=v_{\rm I}-v_{\rm II}\approx 0$, see grey zone in Fig. S5a,b,c. Hence, during a short period of time, the spin system approximately achieves a steady-state with constant non-zero flux around the cycle even without external enforcement. The system, eventually, reaches equilibrium, described by equations in Fig. S4b(iii) and S4b(iv). The equilibrium constant $K_{\rm I}$ describes binding between the enzyme and substrate in the first branch of the chemical kinetics scheme, and $K_{\rm II}$ describes binding between the enzyme and product in the second branch. The expressions for steady-state and equilibrium in chemical kinetics in Fig. S4b(ii) and S4b(iv) are equivalent to the expressions for spin kinetics in Eqs. (S24) and (S26), respectively.

"True" steady-state can be forced by an external agent so that the initial substrate and product concentrations are held constant at values $[\mathbf{S}]_0$ and $[\mathbf{P}]_0$, respectively. Here, after the initial pre-steady-state phase (governed by equations in Fig. S4c(i)), the steady-state is achieved indefinitely (described by equations in Fig. S4c(ii)), see Fig. S5e,f,g. Note that another means to force a steady-state is by imposing a constant external influx of substrate and constant product outflow.

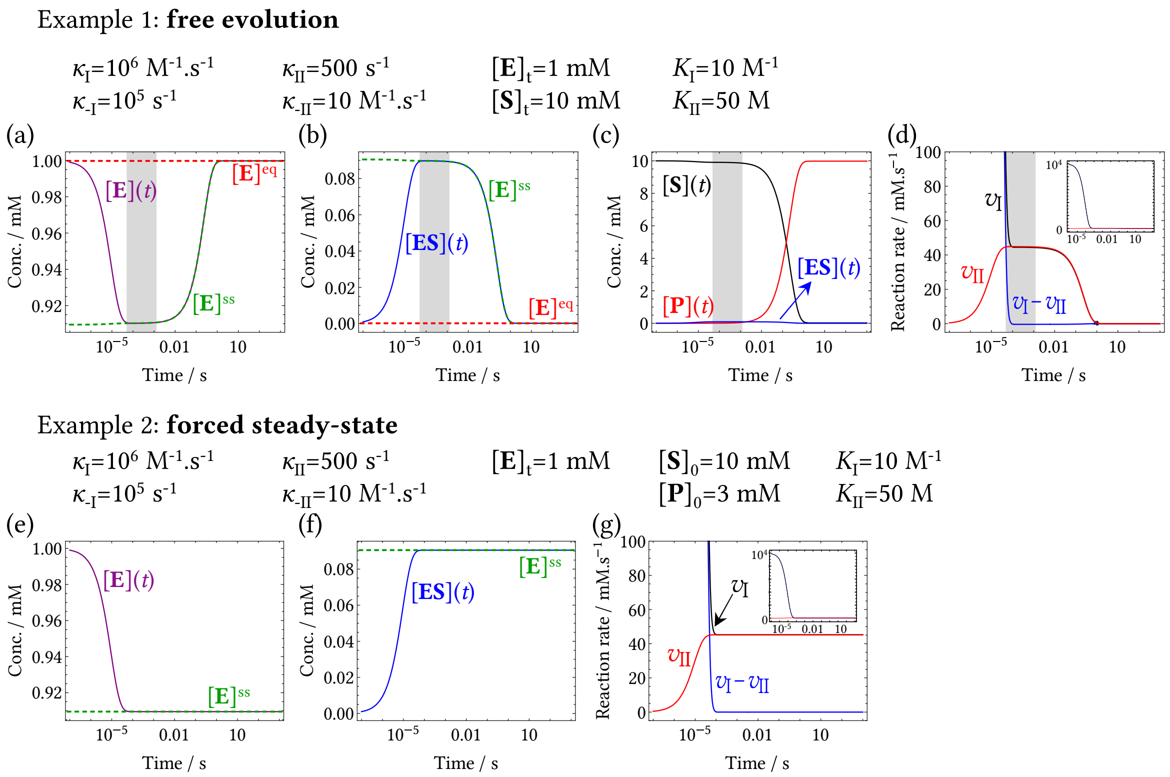


**Figure S5.** Simulated time evolution of the system in Fig. S4a in two different regimes: (**a**–**d**) free evolution (Example 1) and (**e**–**g**) forced steady-state (Example 2). Equilibrium ($[\bullet]^{\rm eq}$) and steady-state ($[\bullet]^{\rm ss}$) concentrations were calculated according to the corresponding equations in Fig. S4b,c. Rates of change of reacting species are equal to $\frac{{\rm d}[\mathbf{S}]}{{\rm d}t}=-v_{\rm I}$, $\frac{{\rm d}[\mathbf{P}]}{{\rm d}t}=-v_{\rm II}$ and $\frac{{\rm d}[\mathbf{ES}]}{{\rm d}t}=-\frac{{\rm d}[\mathbf{E}]}{{\rm d}t}=v_{\rm I}-v_{\rm II}$, and they are depicted in (**d**) and (**g**). Grey zones in (**a**–**d**) show an example of relatively short period of time with approximately constant concentrations. Parameter values used for the simulations are listed in each example.

To summarize, without external enforcement, the steady-state with constant non-zero (cyclic) population flux can be achieved only approximately for a short period of time. On the other hand, the "true" steady-state can be maintained indefinitely by external enforcement. For detection in NMR spectroscopy, free evolution is the experimentally most feasible scenario. However, in order to "catch up" before the system reaches equilibrium, slow dynamics is required ($\kappa_\bullet<1$), which in turn cannot

be measured by using lineshape analysis (more advanced techniques are required, e.g., ZZ-exchange[S17]). Enforcement of a steady-state by an external agent requires modifications of the NMR probe. For example, it is possible to illuminate the sample by LED or laser during measurement,[S18] which allows employment of a photoreaction to maintain the steady-state.

### S6.3 Correlated rotation of dimesityl rings

Here we show an example where there is a larger number of exchange processes between two states. Dimesityl compounds exist in two propeller-like enantiomers[S13,S19,S20] (helical antipodes), $P$ and $M$. Both forms can be interconverted (i.e., an enantiomerization process) by correlated rotation of both mesityl rings. Enantiomerization is accomplished by four different processes, see Fig. S6a (left), each with distinct transition states shown in Fig. S6b. In the transition state of the *zero-ring flip* process, both rings are coplanar and parallel to the C=C plane. In the *one-ring flip* and *two-ring flip* processes, one or two rings are perpendicular to the C=C plane in the transition state, respectively.

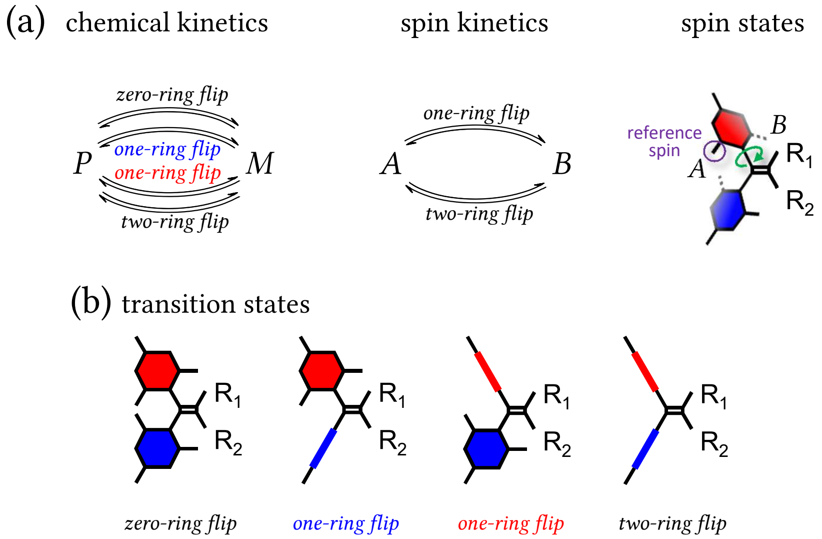


**Figure S6.** Internal dynamics of dimesityl compounds.[S13,S19,S20] (**a**) Chemical and spin kinetics, (**b**) transition states for the present processes.

A particular nuclear spin due to *o*-mesityl methyl group is subject to exchange between two states, see Fig. S6a (middle). However, only two out of four of the processes change the spin state. For example, the highlighted reference spin in Fig. S6a (right) can change its state only by 180° rotation (green arrow), which is possible either in one-ring flip of the red ring or by a two-ring flip process. Zero-ring flip and one-ring flip of the blue ring do not change the spin state, although they change the enantiomeric state. Thus, all *o*-dimesithyl methyl spins undergo two-process two-state chemical exchange.

More complicated molecules with three rings showing correlated rotation[S21,S22] behave analogously to the dimesityl compounds. Both $P$ and $M$ enantiomers are mutually interconverted by one zero-ring flip, three one-ring flips, three two-ring flips and one three-ring flip processes. Spins in *ortho*-positions on the rings undergo exchange between two states only by processes that involve 180° rotation of the corresponding ring. Hence, only one one-ring flip, two two-ring flips and one three-ring flip processes change the state of a particular spin, resulting to four-process two-state exchange.

## S7 Notes on lineshape fitting procedure

### S7.1 Equilibrium, steady-state and time-dependent population modes

During the lineshape analysis, a relationship between populations and transition rate coefficients must be established. Spin kinetics with constant populations (i.e., equilibrium or steady-state mode) implies $\mathbf{K}\mathbf{p} = \mathbf{0}$. In principle, it is possible to analyze out-of-steady-state mode with time-dependent populations. In that case, populations and transition rate coefficients are related using the formula $\frac{\mathrm{d}\mathbf{p}}{\mathrm{d}t} = \mathbf{K}\mathbf{p}$. However, the relaxation time (equal to $1/(2k)$ in symmetric two-state spin kinetics) to reach equilibrium or to reach steady-state (do not mistake for longitudinal or transverse magnetization relaxation) should be long compared to the NMR measurement time (i.e., spectra acquisition time). For example, a measurement time of 30 s enables the capture of relaxation processes of at least 5 min duration, implying a transition rate coefficient less than 0.01 $\mathrm{s}^{-1}$. However, lineshape analysis cannot be used to observe such a value due to undetectable broadening. On the other hand, in a slow exchange regime, the populations can be determined by integration of isolated peaks and the transition rate coefficients obtained directly from the formula $\frac{\mathrm{d}\mathbf{p}}{\mathrm{d}t} = \mathbf{K}\mathbf{p}$ without lineshape fitting (real-time NMR[S23]).

### S7.2 Interdependency of parameters for general $N$-state exchange

As mentioned in Sec. "Kinetics of three-state exchange" of the main manuscript, the $N$-state exchange kinetics is described by interdependent populations and transition rate coefficients. Here, we follow up the discussion from Sec. "Kinetics of three-state exchange" regarding which combination of parameters can be chosen as independent. This is important for the lineshape fitting procedure. It is occasionally possible to obtain values of certain parameters by an independent experiment/data processing method (e.g., populations can be obtained by direct integration if the peaks are isolated) or methods other than NMR spectroscopy (e.g., UV-vis, FTIR). Values obtained in this manner should be considered as being independent and kept constant during the fitting procedure of one particular spectrum. At least one parameter $p_j$ must be considered as dependent, since all populations sum to unity.

The independent parameters should be selected cautiously as illustrated in the following discussion. For instance, in one-process two-state exchange, pairs of parameters $\{k_{AB},k_{BA}\}$ or $\{k_{AB},p_A\}$ can be fitted while calculating the other two parameters using Eq. (6), although the pair $\{p_A,p_B\}$ cannot be fitted since the populations are mutually dependent, $p_A + p_B = 1$.

As an another example, consider three-state exchange kinetics in the steady-state regime (i.e., six independent parameters), assuming all populations $p_j^{\mathrm{ss}}$ are known (reducing the number of independent parameters to four). Then two populations and four transition rate coefficients can serve as independent parameters (out of the total nine parameters describing the kinetics). For example, if we choose $k_{AB}$ and $k_{AC}$ to be the dependent parameters (and $k_{BA}$, $k_{CA}$, $k_{BC}$ and $k_{CB}$ as being the independent parameters), it follows from Eq. (15) that

$$k_{AB} = \frac{(k_{BA} + k_{BC})p_B^{\mathrm{ss}} - k_{CB}p_C^{\mathrm{ss}}}{p_A^{\mathrm{ss}}}\,, \tag{S27a}$$

$$k_{AC} = \frac{(k_{CA} + k_{CB})p_C^{\mathrm{ss}} - k_{BC}p_B^{\mathrm{ss}}}{p_A^{\mathrm{ss}}}\,. \tag{S27b}$$

However, the choice of two dependent transition rate coefficients is not arbitrary. Pairs $\{k_{AB},k_{BA}\}$, $\{k_{AC},k_{CA}\}$ or $\{k_{BC},k_{CB}\}$ cannot be chosen as dependent. More specifically, if one of these pairs is selected as dependent, then the other four independent transition rate coefficients should have arbitrary values, although this situation might violate the condition for a steady-state as expressed in the following equations (which are reformulation of $\mathbf{K}\mathbf{p} = \mathbf{0}$ using population fluxes for three-state kinetics)

$$J_{BA} + J_{CA} = 0\,, \tag{S28a}$$

$$J_{AB} + J_{CB} = 0\,, \tag{S28b}$$

$$J_{AC} + J_{BC} = 0\,. \tag{S28c}$$

For example, if we select the pair $\{k_{AB},k_{BA}\}$, it would mean that the transition fluxes $J_{AC}$ and $J_{BC}$ could be independently set to arbitrary values since they do not depend on $k_{AB}$ or $k_{BA}$ (using Eq. (9)). However, this contradicts the condition for steady-state kinetics in Eq. (S28c). Note that the transition rate coefficients $k_{AB}$ and $k_{AC}$ in Eq. (S27) might become negative if the transition rate coefficients $k_{CB}$ or $k_{BC}$ are large enough (at fixed populations). Such configurations are not physical and during the fitting procedure constraints $k_{AB} > 0$ and $k_{AC} > 0$ should be used.

It is instructive to describe, how the relations in Eq. (S27) are modified at equilibrium. Using the definitions for population

flux, Eq. (9), they can be rewritten as

$$k_{AB} = \frac{p_B^{\mathrm{ss}}}{p_A^{\mathrm{ss}}} k_{BA} + \frac{1}{p_A^{\mathrm{ss}}} J_{BC}\,, \tag{S29a}$$

$$k_{AC} = \frac{p_C^{\mathrm{ss}}}{p_A^{\mathrm{ss}}} k_{CA} + \frac{1}{p_A^{\mathrm{ss}}} J_{CB}\,. \tag{S29b}$$

In the case of equilibrium $J_{BC} = 0$ and $J_{CB} = 0$ (Sec. "Two-state exchange at equilibrium and at steady-state"), hence the above relations become identical with the equilibrium conditions, Eq. (11).

Next, we will discuss in detail the determination of the number of independent parameters describing general $N$-state exchange kinetics, assuming one reversible process between every pair of states. Note that in special cases, some transition rate coefficients can be equal (e.g., half-symmetric three-state exchange) or some can be equal to zero (consecutive three-state exchange). Generally, for $N$-states, there are $N$ populations and $N(N-1)$ corresponding transition rate coefficients, therefore in total $N + N(N-1) = N^2$ parameters describing the kinetics.

Definition of a steady-state implies $N-1$ independent conditions ($\mathcal{J}_k = 0$) and the sum of all populations implies one other condition ($\sum_j p_j = 1$). Thus, in total there are $N^2 - (N-1) - 1 = N(N-1)$ independent parameters. If all populations are known, there are in total $N(N-1)$ transition rate coefficients, which are again bound by $N-1$ conditions ($J_{jk} + J_{kj} = 0$). This means that in total $N(N-1) - (N-1) = (N-1)^2$ transition rate coefficients are independent.

On the other hand, an equilibrium implies $N(N-1)/2$ conditions ($J_{jk} = 0$), which means $N^2 - N(N-1)/2 - 1 = N(N+1)/2 - 1$ independent parameters. Equilibrium with known populations analogously gives $N(N-1) - N(N-1)/2 = N(N-1)/2$ independent transition rate coefficients. An overview of the number of independent parameters is shown in Table S3.

**Table S3.** Number of independent parameters for $N$-state kinetic schemes, assuming one reversible process between every pair of states.

| | Number of independent parameters | | | |
|---|---|---|---|---|
| | steady-state | | equilibrium | |
| No. of states | unknown $p_j^{\mathrm{ss}}$ | known $p_j^{\mathrm{ss}}$ | unknown $p_j^{\mathrm{eq}}$ | known $p_j^{\mathrm{eq}}$ |
| 2 | — | — | 2 | 1 |
| 3 | 6 | 4 | 5 | 3 |
| 4 | 12 | 9 | 9 | 6 |
| $N$ | $N(N-1)$ | $(N-1)^2$ | $N(N+1)/2-1$ | $N(N-1)/2$ |

### S7.3 Steady-state lineshape in three-state exchange

In Sec. S6.1 we have shown that, in the case of two-process two-state exchange, a steady-state cannot be differentiated from an equilibrium based only on the NMR spectral lineshape because of the mathematical equivalence of the corresponding formulae. In the case of three-state exchange, a steady-state is possible in the general case (Table 1 in the main manuscript), where six parameters are independent. An equilibrium condition reduces the number of free parameters to five. Although the corresponding lineshapes (Eq. (13) in the main manuscript) for steady-state and equilibrium differ mathematically, they can be fitted to each other, as illustrated in the three examples shown in Fig. S7. Thus, it is also not possible to distinguish a steady-state from an equilibrium in this case. However, if some of the transition rate coefficients are measured separately (by a different NMR method or a non-NMR experimental technique) and held constant during the fitting procedure, then the steady-state can by distinguished from an equilibrium.

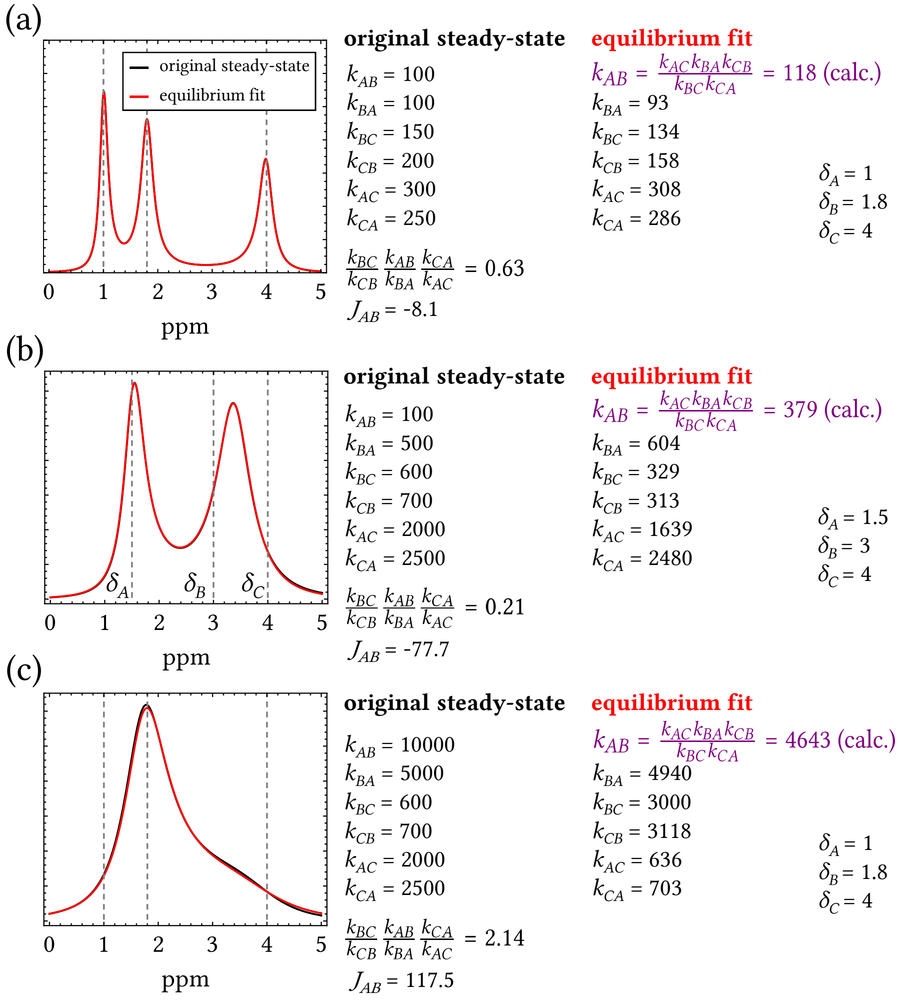


**Figure S7.** Three-state exchange lineshape in a steady-state (black line, 6 free parameters), fitted with an equilibrium lineshape (red line, 5 free parameters). Three different model situations with different transition rate coefficients are given in (**a**), (**b**) and (**c**). Deviation of the ratio $\frac{k_{BC}}{k_{CB}}\frac{k_{AB}}{k_{BA}}\frac{k_{CA}}{k_{AC}}$ from unity as well as deviation of population flux $J_{AB}$ from zero describe how far the system is from equilibrium (while still in steady-state), see Sec. "Kinetics of three-state exchange". Used parameters: $R_2^A = R_2^B = R_2^C = 30\ s^{-1}$, $\nu_0 = 500.13$ MHz. Larmor frequencies $\delta_j$ in ppm units are listed for each example (note that they are the same for steady-state simulation and the equilibrium fit).

### S7.4 Limitations of lineshape analysis

Two approaches can be considered to estimate the range of measurable transition rate coefficients by NMR lineshape analyses. The first is based on the NMR instrument capability (magnetic field strength), and the second is the actual lineshape fitting limitations (sensitivity). In both approaches, we use two singlets under symmetric two-state exchange (without J-couplings) for the illustration.

**NMR instrument capability**

The main NMR instrument specifications are the magnetic field strength (in our considerations, $B_0$ = 11.7 T, $\nu_0$ = 500.13 MHz for $^1$H), spectral width ($\Delta\nu_{\mathrm{width}}$ (in Hz), $\Delta\omega_{\mathrm{width}} = 2\pi\Delta\nu_{\mathrm{width}}$ (in rad.s$^{-1}$), $\Delta\delta_{\mathrm{width}} = \Delta\nu_{\mathrm{width}}/\nu_0$ (in ppm)), and spectral resolution ($\Delta\nu_{\mathrm{res}}$ (in Hz), $\Delta\omega_{\mathrm{res}} = 2\pi\Delta\nu_{\mathrm{res}}$ (in rad.s$^{-1}$), $\Delta\delta_{\mathrm{res}} = \Delta\nu_{\mathrm{res}}/\nu_0$ (in ppm)); more precisely, the possibility to well-resolve two resonances). In this approach, we consider (similarly to Bryant[S24]) that one should be able to observe (at least) coalescence in the spectrum. The simplest relation between the transition rate coefficient at coalescence $k_{\mathrm{c}}$ and the separation of resonances $\Delta\omega$ [rad.s$^{-1}$] = $2\pi \times \nu_0$ [MHz] $\times\, \Delta\delta$ [ppm] is in Eq. (S18), i.e., $k_{\mathrm{c}} = \Delta\omega/(2\sqrt{2})$. Using the limit for spectral resolution $\Delta\omega_{\mathrm{res}}$ and limit for spectral width $\Delta\omega_{\mathrm{width}}$ (depending on the nucleus of interest), we obtain $k_{\mathrm{c}}$ values listed in Table S4. It should be noted that the lower limit of $k$ (ca. $k_{\mathrm{c}}$ = 4.4 s$^{-1}$) requires narrow resonances with full width at half maximum around 3 Hz (which is usually not accessible). The upper bound is, on the other hand, underestimated since the spectrum changes its shape substantially above $k_{\mathrm{c}}$. Through simulations, we can estimate that there is still a well-resolvable change in the spectral shape even at $k \approx 100k_{\mathrm{c}}$ (therefore, Table S4 contains an upper bound for $k$ value $100k_{\mathrm{c}}$). The factor of 100 is also supported by the analyses using lineshape fitting sensitivity shown below (see Figure S8b; move from coalescence point for about two-orders of magnitude to higher $k$ yields still reasonable sensitivity).

**Table S4.** The range of transition rate coefficients as determined from the NMR instrumental capabilities.

| Nucleus | $\nu_0$ / MHz | Spectral resolution limit (lower bound for $k$) | | | | Spectral width limit (upper bound for $k$) | | | |
|---|---|---|---|---|---|---|---|---|---|
| | | $\Delta\delta_{\mathrm{res}}$ / ppm | $\Delta\nu_{\mathrm{res}}$ / Hz | $\Delta\omega_{\mathrm{res}}$ / rad.s$^{-1}$ | $k_{\mathrm{c}}$ / s$^{-1}$ | $\Delta\delta_{\mathrm{width}}$ / ppm | $\Delta\nu_{\mathrm{width}}$ / Hz | $\Delta\omega_{\mathrm{width}}$ / rad.s$^{-1}$ | $100k_{\mathrm{c}}$ / s$^{-1}$ |
| $^1$H | 500.13 | 0.0040 | 2 | 12.57 | 4.4 | 20 | 10000 | 62850 | $2.2\times10^6$ |
| $^{13}$C | 125.76 | 0.032 | 4 | 25.13 | 8.9 | 240 | 30180 | 189600 | $6.7\times10^6$ |
| $^{19}$F | 470.59 | 0.0085 | 4 | 25.13 | 8.9 | 400 | 188200 | 1183000 | $4.2\times10^7$ |

**Lineshape fitting limitations (sensitivity)**

The sensitivity of lineshape fitting depends on peak separation, transverse relaxation rates and the transition rate coefficients. Here, we illustrate the case of symmetric two-state exchange as shown in Fig. S8. The original lineshape (black line) with $k$ = 1000 s$^{-1}$ is "perturbed" by increasing $k$ by 10% (red line), see Fig. S8a. The perturbed spectrum is then compared with the original. The grey zone denotes the difference (area in absolute value) between the original and perturbed spectrum. The larger the difference, the greater the sensitivity of the original lineshape to perturbation (to a small change in $k$). The sensitivity $\epsilon$ can be quantified as the ratio of the difference area (in absolute value) and the original peak area.

$$\epsilon = \frac{|\text{difference area}|}{\text{peak area}}\,. \tag{S30}$$

Note that the original and perturbed lineshapes have the same area. The dependence of $\epsilon$ on $k$ for different values of $|\delta_A - \delta_B|$ and $R_2$ is given in Fig. S8b. The original lineshape is perturbed by increasing the transition rate coefficient by 10%. For $k < 10$ s$^{-1}$ (slow exchange regime) or for $k > 10^5$ s$^{-1}$ (fast exchange regime), the sensitivity drops under 1%. Note that $\epsilon$ has a maximum at the coalescence point (denoted by the full circle). An increase of separation $|\delta_A - \delta_B|$ shifts the sensitivity maximum to higher $k$ values. On the other hand, an increase in $R_2$ value decreases the sensitivity. Thus the lineshape fitting procedure is more accurate when the intrinsic $R_2$ value of exchanging resonances is small (i.e., narrow peaks). In this idealized simulated example, it can also be seen that the reasonable sensitivity range is from about 10 s$^{-1}$ to $10^5 - 10^6$ s$^{-1}$. In reality, the sensitivity drops even faster due to spectral noise, various baseline artifacts or overlapping signals from impurities. However, the range of accessible $k$ values obtained from this simple illustration is similar to those obtained from the NMR instrument capability considerations.

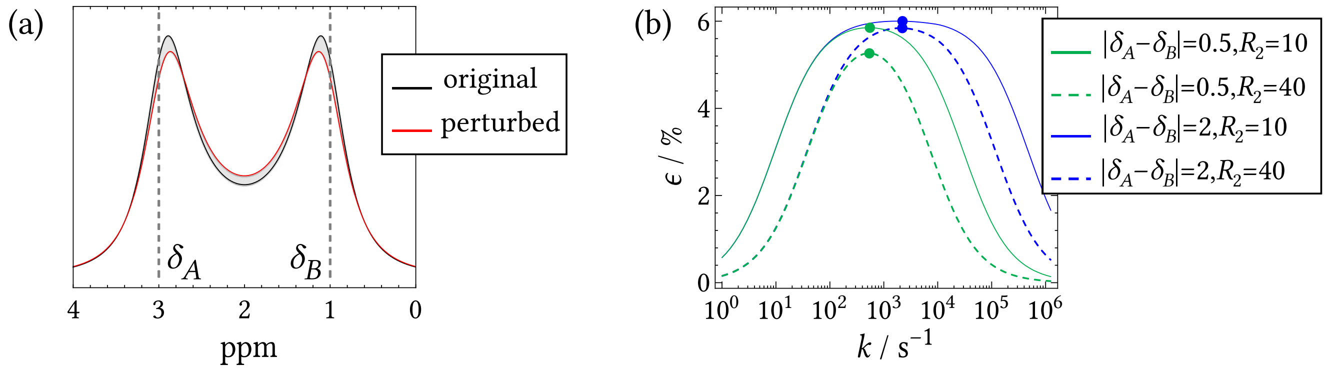


**Figure S8.** Sensitivity of lineshape fitting; an example of symmetric two-state exchange in $^1$H NMR spectrum. (**a**) Original lineshape with $k$ = 1000 s$^{-1}$ (black line) and perturbed lineshape with $k$ = 1100 s$^{-1}$, i.e., 10% increased (red line). The grey zone denotes the difference between the two lineshapes. Other parameters for both lineshapes: $R_2^A = R_2^B = 40$ s$^{-1}$, $\nu_0$ = 500.13 MHz. (**b**) Plot of sensitivity $\epsilon$ of the lineshape to 10% perturbation in $k$ for different values of $\Delta\delta = |\delta_A - \delta_B|$ and $R_2$. The sensitivity has a maximum at the coalescence point (denoted by full circle).

## S8 Four-state exchange in the system of Kartha et al.[S1]

Internal dynamics of a molecular rotor realized by an overcrowded butterfly-shape alkene (**FDF**) has been described by Kartha et al.[S1] The **FDF** molecule has two symmetrically positioned rotors attached to a central stator. The rotors can take either a position $P$ or $M$ relative to the stator. The *cis* isomer $(P,M)^{\mathrm{c}}$ is symmetric and identical to $(M,P)^{\mathrm{c}}$, while *trans* isomers $(P,P)^{\mathrm{t}}$ and $(M,M)^{\mathrm{t}}$ are chiral and distinct. As shown in Fig. S9a, there are three chemical species in total, which can mutually interconvert by flipping of the rotors involving two different processes: (i) 'rim flip' described by $k_{\mathrm{r}}$ (with a transition state where rotor and stator are parallel), and (ii) 'double bond flip' described by $k_{\mathrm{d}}$ (having a transition state where rotor and stator are perpendicular). The dynamics was detected in NMR by a spin located at the rotors, which can be in four states, namely $(\mathrm{E^t})^A$, $(\mathrm{E^c})^B$, $(\mathrm{F^t})^C$ and $(\mathrm{F^c})^D$, see Fig. S9b and the corresponding kinetic matrix in Fig. S9c. The signals $(\mathrm{E^t})^A$ and $(\mathrm{F^t})^C$ belong to the *trans* conformation (enantiomers $(P,P)^{\mathrm{t}}$ and $(M,M)^{\mathrm{t}}$ are indistinguishable in the NMR spectrum), the signals $(\mathrm{E^c})^B$ and $(\mathrm{F^c})^D$ correspond to the *cis* isomer. The capital letter subscripts denote the spin states.

Both *cis* and *trans* isomers generally have different standard Gibbs energy and are differently populated depending on the solvent (i.e., generally $G^\circ_{trans} - G^\circ_{cis} \neq 0$), which can be described by an equilibrium constant $K_{\mathrm{t/c}}$=[*trans*]/[*cis*]. However, in the case of *o*-dichlorbenzene solvent both *cis* and *trans* isomers have approximately the same energy and $K_{\mathrm{t/c}} \approx 1$, see Fig. S9d,e. The value of $K_{\mathrm{t/c}}$ can be calculated directly from the spin kinetics as indicated in Fig. S9d, the symmetric scheme is compatible with the value $K_{\mathrm{t/c}} = 1$. On the other hand, asymmetric Fig. S9e–h assumes $G^\circ_{trans} \neq G^\circ_{cis}$, which yields $K_{\mathrm{t/c}} \neq 1$. Hence, the ratio of *cis* and *trans* isomers can be directly related to the transition rate coefficients, see Fig. S9i.

As indicated in Fig. S9e, the energy barrier for the rim flip process $\Delta G^\ddagger_{\mathrm{r}}$ is lower than the barrier for double bond flip $\Delta G^\ddagger_{\mathrm{d}}$. Thus, at lower temperatures (Fig. S10 left), rim flipping is much faster than double bond flipping. This enabled (together with sufficient frequency separation of states $\mathrm{E}^\bullet$ from $\mathrm{F}^\bullet$) separate fitting of a two-state lineshape to the $(\mathrm{E^t})^A$ and $(\mathrm{E^c})^B$ exchanging spins, blue lines in Fig. S10. At higher temperatures (Fig. S10 right), rim flip is already very fast and $(\mathrm{E^t})^A$ and $(\mathrm{E^c})^B$ merge (resonances of $(\mathrm{F^t})^C$ and $(\mathrm{F^c})^D$ overlap). This enabled fitting of the averaged states $\mathrm{E}^\bullet$ and $\mathrm{F}^\bullet$ with the two-state lineshape as a function of the double bond flip transition rate coefficient $k_{\mathrm{d}}$, red lines in Fig. S10.

We have shown in Fig. S9 that the underlying spin kinetics is comprised of four states. Therefore, it is possible to fit the experimental data from figure 1 in Kartha et al.[S1] with a four-state lineshape. We have used the symbolic derivation of four-state lineshape from Sec. S1.2 and imposed the symmetry from Fig. S9c,d, namely

```
fourStateLineshapeComplex /. {kAB -> kr, kBA -> kr, kAC -> 0, kCA -> 0, kAD -> kd, kDA -> kd,
  kBC -> kd, kCB -> kd, kBD -> 0, kDB -> 0, kCD -> kr, kDC -> kr,
  pA -> 1/4, pB -> 1/4, pC -> 1/4, pD -> 1/4};
```

We have remodeled the true four-state lineshape over the whole temperature range, see black lines in Fig. S10. Our four-state model describes the experimental data very well, especially in the temperature interval of 75–100 °C it provides significant improvement over the two separate two-state fittings.

The (symmetrized) four-state lineshape in its limit cases can be directly mapped on the two-state lineshape. In the low

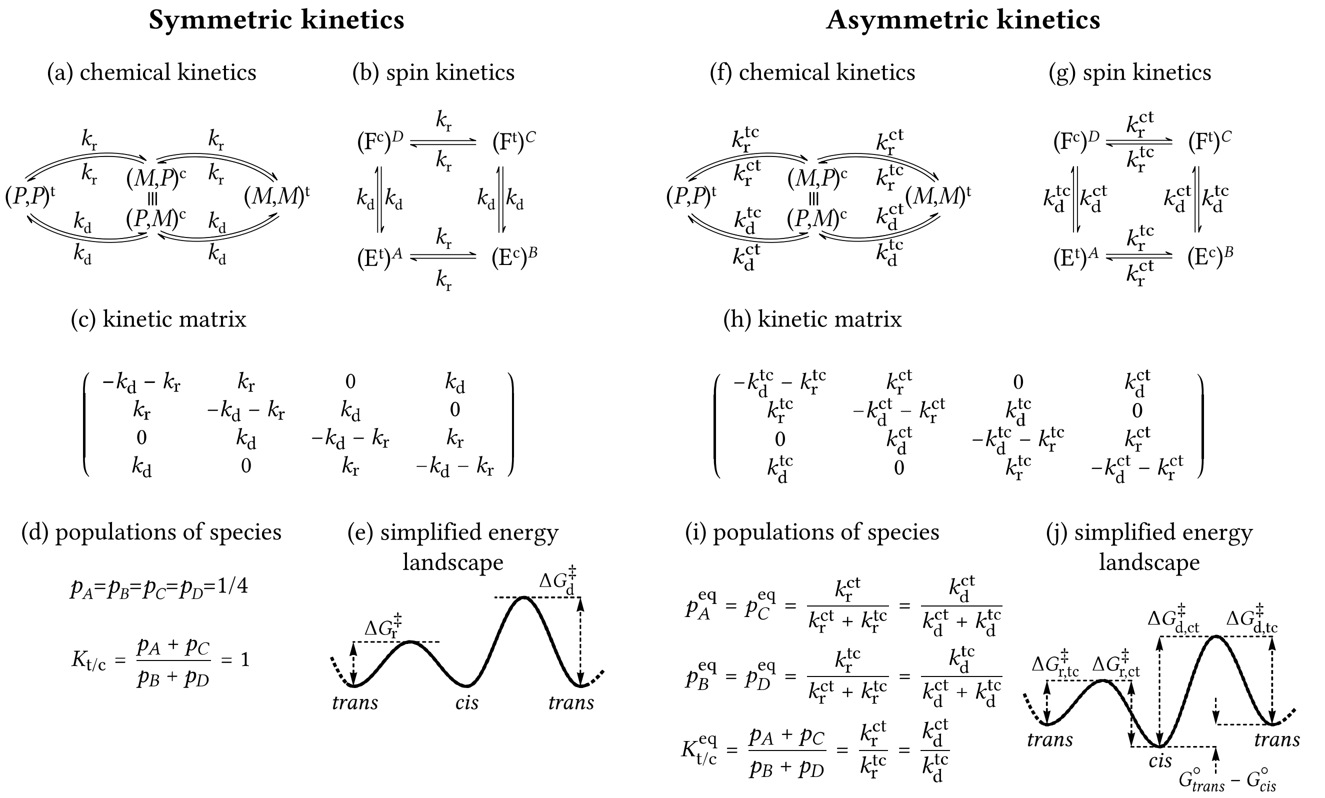


**Figure S9.** Chemical kinetics with three species and corresponding four-state spin kinetics from Kartha et al.[S1] Experimental data in their figure 1[S1] can be described by the symmetric kinetic scheme (**a**–**e**), data in their figure 4[S1] can be described by the asymmetric kinetic scheme (**f**–**j**). Simplified energy landscape plots in (**e**) and (**j**) describe transition between the *cis* state and one particular *trans* state (e.g., $(P,P)^{\mathrm{t}}$), the complete scheme is two-dimensional and can be found in SI of Kartha et al.[S1]

temperature region applying the limit $k_{\mathrm{d}} \to 0$ yields

$$\lim_{k_{\mathrm{d}}\to 0} S_{\text{sym. four-state exch.}}(\omega) = \frac{M_0}{2}\frac{1/2\alpha_A + 1/2\alpha_B + 2k_{\mathrm{r}}}{\alpha_A\alpha_B + k_{\mathrm{r}}\alpha_B + k_{\mathrm{r}}\alpha_A} + \frac{M_0}{2}\frac{1/2\alpha_C + 1/2\alpha_D + 2k_{\mathrm{r}}}{\alpha_C\alpha_D + k_{\mathrm{r}}\alpha_D + k_{\mathrm{r}}\alpha_C}\,. \tag{S31}$$

By comparison with Eq. (7), this equation describes, how the four-state system at the limit effectively disconnects into two independent two-state systems governed by $k_{\mathrm{r}}$ (the first including states $A$ and $B$, the second including states $C$ and $D$). The high temperature region can be described by the limit $k_{\mathrm{r}} \to \infty$, giving

$$\lim_{k_{\mathrm{r}}\to \infty} S_{\text{sym. four-state exch.}}(\omega) = M_0 \frac{1/2\frac{\alpha_A+\alpha_B}{2} + 1/2\frac{\alpha_C+\alpha_D}{2} + 2k_{\mathrm{d}}}{\frac{\alpha_A+\alpha_B}{2}\frac{\alpha_C+\alpha_D}{2} + k_{\mathrm{d}}\frac{\alpha_C+\alpha_D}{2} + k_{\mathrm{d}}\frac{\alpha_A+\alpha_B}{2}}\,, \tag{S32}$$

which stands for a two-state exchange lineshape governed by $k_{\mathrm{d}}$, where the states $A$ and $B$ as well as $C$ and $D$ are merged, and thus form a two-state reduced equivalent scheme (similarly to the derivation of Eq. (19) in Sec. "Exchange schemes containing a fast process").

In the equilibrium (no preferred direction of rotation) asymmetric case, the energy barriers are bound together by the energy landscape geometry according to Fig. S9j, i.e., $2\left[(\Delta G^{\ddagger}_{\mathrm{r,tc}} - \Delta G^{\ddagger}_{\mathrm{r,ct}}) + (\Delta G^{\ddagger}_{\mathrm{d,ct}} - \Delta G^{\ddagger}_{\mathrm{d,tc}})\right] = 0$. Substitution from the Eyring equation, Eq. (23), yields the following equilibrium condition (cf. Eq. (14))

$$\frac{k^{\mathrm{tc}}_{\mathrm{d}}}{k^{\mathrm{ct}}_{\mathrm{d}}}\frac{k^{\mathrm{ct}}_{\mathrm{r}}}{k^{\mathrm{tc}}_{\mathrm{r}}} = 1\,. \tag{S33}$$

To summarize briefly, the detailed analyses of *cis-trans* interconversion in overcrowded butterfly-shape alkene (**FDF**) using full four-state spin kinetics gives a much deeper understanding of the mechanism and also justifies the use of two separate two-state spin kinetics in two different temperature regions (as was used by Kartha et al.[S1]).

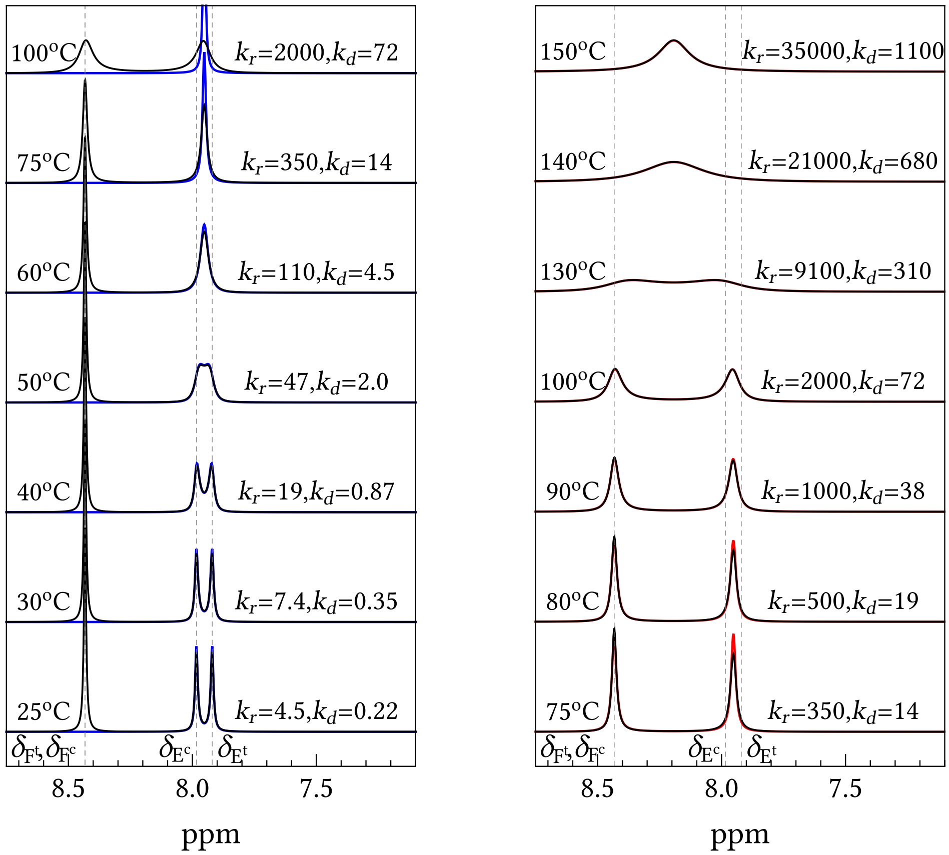


**Figure S10.** Reconstruction of four-state exchange from figure 1 in Kartha et al.[S1] Blue line denotes two-state fit of rim flip process ($k_\mathrm{r}$) in the slow regime of the double bond flip process ($k_\mathrm{d}$), red line denotes two-state fit of the double bond flip process ($k_\mathrm{d}$) in the fast regime of the rim flip process ($k_\mathrm{r}$). Both fits are taken from the original article. Black line denotes our four-state lineshape reconstruction, which reproduces the experimental data (not shown) very well. Transition rate coefficients $k_\mathrm{r}$ and $k_\mathrm{d}$ were calculated from the temperature using the Eyring equation, Eq. (23), (with the transition probability $\eta = 1$) and $\Delta G^\ddagger$ values given in Kartha et al.: $\Delta G^\ddagger_\mathrm{r} = 72300 - 10.2T$ J.mol$^{-1}$ and $\Delta G^\ddagger_\mathrm{d} = 68700 + 27.0T$ J.mol$^{-1}$.

## S9 Explanation of fitting results in work by Feng et al.[S2]

An article by Feng et al.[S2] introducing *NmrLineGuru*, software for (numeric) fitting of multi-state exchange spectral lineshapes, presents simulated spectra and their fitting. They successfully fit a particular case of consecutive three-state exchange spectra during the simulated titration process employing a two-state exchange model. The authors comment on this fact as an interesting feature of this particular setup. However, they could not fit another case involving a similar consecutive scheme, which showed a shift of one of the resonance maxima during the simulated titration process. Our models developed in this study allow for more profound analyses of these systems in terms of 'reduced equivalent schemes'. Here, we extend the discussion from Sec. "Exchange schemes containing a fast process" in the main manuscript and elaborate on the possibility to fit a three-state exchange with a two-state lineshape. Also, we give the relationship between the fitted parameters and their true physical meaning.

In the paper by Feng et al.,[S2] figure 4d shows a simulated titration of a protein **P** with a ligand **L**, where the formation of the complex induces a protein conformational change (isomerization) to **P'L**, see Fig. S11a. The kinetics is described by two equilibrium constants (dissociation and isomerization) and four reaction rate coefficients (we have modified the notation from the paper of Feng[S2] and relabeled the reaction rate coefficients to $\kappa_\bullet$ in order to match our notation), namely

$$K_\mathrm{D} = \frac{[\mathbf{P}][\mathbf{L}]}{[\mathbf{PL}]} = \frac{\kappa_\mathrm{off}}{\kappa_\mathrm{on}} \text{ (dissociation)}, \tag{S34a}$$

$$K_\mathrm{eq} = \frac{[\mathbf{PL}]}{[\mathbf{P'L}]} = \frac{\kappa_\mathrm{rev}}{\kappa_\mathrm{fwd}} \text{ (isomerization)}. \tag{S34b}$$

For our purpose, let us assign the chemical species **P**, **PL** and **P'L** to spin states $A$, $B$ and $C$, respectively, see Fig. S11b. A

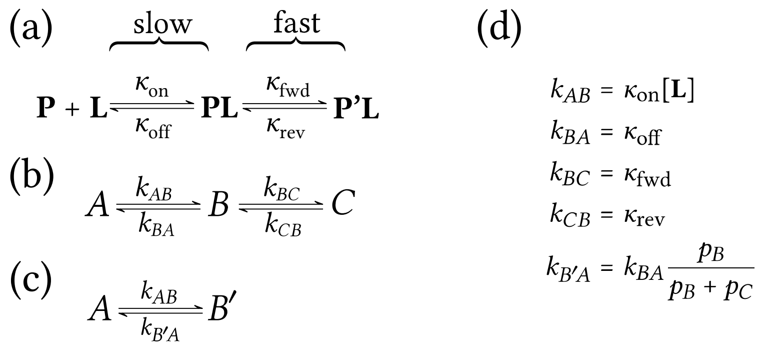


**Figure S11.** Protein–ligand binding with induced protein isomerization describing data in figure 4d in Feng et al.[S2] (**a**) Chemical kinetics scheme and (**b**) corresponding full spin kinetics scheme. (**c**) Reduced spin kinetics scheme at fast isomerization process. (**d**) Correspondence between chemical and spin kinetic schemes.

comparison of the chemical and spin kinetics schemes provides relations between transition and reaction rate coefficients, see Fig. S11d.

The association/dissociation step is in a slow exchange regime and the isomerization in a fast regime. Thus, the NMR spectra of the protein contained two peaks, one due to free **P** and another due to an averaged signal of **PL** and **P'L**. Our analysis in Sec. "Exchange schemes containing a fast process" in the main manuscript showed that under fast exchange, the full consecutive spin kinetics scheme can be replaced by a reduced equivalent scheme, where averaged states $B$ and $C$ are represented by one state $B'$, see Fig. S11c (or equivalently Fig. 4 in the main manuscript). In the reduced scheme in Fig. S11c, the value of $k_{AB}$ is the same as in the full scheme, however, $k_{BA}$ is replaced by $k_{B'A}$. Equation (20) in the main manuscript implies that $k_{B'A} = k_{BA}p_B/(p_B + p_C)$. Using Eq. (S34), this can be further reformulated as

$$k_{B'A} = \kappa_{\text{off}}\frac{p_B}{p_B + p_C} = \kappa_{\text{off}}\frac{[\mathbf{PL}]}{[\mathbf{PL}] + [\mathbf{P'L}]} = \kappa_{\text{off}}\frac{K_{\text{eq}}}{K_{\text{eq}} + 1}\,. \tag{S35}$$

The dissociation constant depends on the transition rate coefficients as $K_{\text{D}} = k_{BA}[\mathbf{L}]/k_{AB}$. In the reduced equivalent scheme, we define an "apparent" dissociation constant $K'_{\text{D}} = k_{B'A}[\mathbf{L}]/k_{AB}$, which can be further related to the true equilibrium constants using Eq. (S35) as

$$K'_{\text{D}} = k_{B'A}\frac{[\mathbf{L}]}{k_{AB}} = \kappa_{\text{off}}\frac{K_{\text{eq}}}{K_{\text{eq}} + 1}\frac{[\mathbf{L}]}{\kappa_{\text{on}}[\mathbf{L}]} = K_{\text{D}}\frac{K_{\text{eq}}}{K_{\text{eq}} + 1}\,. \tag{S36}$$

The latter two equations (Eqs. (S35) and Eq. (S36)) will be utilized in interpretation of the results obtained from two-state fits.

The program *NmrLineGuru* uses the parameters $\kappa_{\text{off}}$, $K_{\text{D}}$, $\kappa_{\text{rev}}$ and $K_{\text{eq}}$ for fitting of three-state exchange lineshape according to Fig. S11a. These parameters were successfully retrieved for the data of figure 4d in Feng et al.,[S2] see Table S5. The two-state model also provided a good fit of these data (figure S3 in Feng et al.[S2]) using only the dissociation part of Fig. S11a and $\kappa_{\text{off}}$ and $K_{\text{D}}$ as fitting parameters. In fact, these two fitting parameters are different from the true $\kappa_{\text{off}}$ and $K_{\text{D}}$, but they are equal to the parameters $k_{B'A}$ and $K'_{\text{D}}$ in the reduced equivalent scheme, Fig. S11c. Hence, the values expected in two-state model fitting can be calculated using Eqs. (S35) and (S36),

$$k_{B'A} = 5\ \text{s}^{-1} \times \frac{1}{1+1} = 2.5\ \text{s}^{-1}\,,$$
$$K'_{\text{D}} = 10\mu\text{M} \times \frac{1}{1+1} = 5\mu\text{M}\,.$$

In the paper by Feng et al.[S2] they could not obtain the value of $k_{B'A}$, since it was too low for the lineshape fitting method. They did however obtain a value of $K'_{\text{D}} = 4.5 \pm 0.8\ \mu$M, which is very close to our predicted value of 5 $\mu$M calculated using the above model, see overview in the Table S5.

Because the isomerization step was much faster than dissociation and $K_{\text{eq}} = 1$, the populations of averaged states $B$ and $C$ were equal throughout the titration (figure 4b in Feng et al.[S2]) and both peaks corresponding to $B'$ and $A$ remained at the same frequency. Therefore, the program *NmrLineGuru* could successfully fit the three-state lineshapes with two-state model, as they did not have to correct for the averaged peak position in the two-state model (where $\omega_{B'} = (p_B\omega_B + p_C\omega_C)/(p_B + p_C)$ remained constant during the simulated titration). Contrary to this situation, the data from figure 4e in Feng et al.[S2] (although in similar conditions with slow and fast exchange regimes) could not be fitted with the two-state model by the *NmrLineGuru* program because the resonance of averaged state $B'$ changes position (correction for shift in $\omega_{B'} = (p_B\omega_B + p_C\omega_C)/(p_B + p_C)$ is needed).

**Table S5.** Parameters of fitting a three-state exchange from figure 4d and figure S3 in Feng et al.[S2] Their results from fitting with a two-state model are explained with our reduced equivalent scheme.

| | $K_\mathrm{D}$ / $\mu$M | $K_\mathrm{eq}$ | $\kappa_\mathrm{off}$ / s$^{-1}$ | $\kappa_\mathrm{rev}$ / s$^{-1}$ |
|---|---|---|---|---|
| true parameters[a] | 10 | 1 | 5 | 5000 |
| three-state fit[b] | 11±2 | 1.0±0.1 | 2.6±1.5 | 3100 |
| two-state fit[c] | 4.5±0.8 ($K'_\mathrm{D}$) | | too low | |
| our model[d] | 5 ($K'_\mathrm{D}$) | | 2.5 ($k_{B'A}$) | |

[a]Input parameters for the simulated titration in Feng et al.[S2] [b]True model fit from Feng et al.[S2] [c]Good fit with the simplified model in Feng et al.[S2] [d]Our model using the reduced equivalent scheme.

## S10 Three-state exchange with appended fast exchanging state

Let us consider a three-state scheme with appended fourth state and assume that this appended state is under fast exchange, see Fig. S12a. Similarly to the example from Sec. "Exchange schemes containing a fast process" in the main manuscript, the limit of $k_{CD}, k_{DC} \to \infty$ while maintaining $k_{CD}/k_{DC} = p_D/p_C$ constant yields three-state spectral lineshape (Eq. (13)) represented by a reduced equivalent scheme (Fig. S12b). From this scheme we obtain the following relationships

$$k_{C'A} = \frac{k_{CA} p_C}{p_C + p_D}, \tag{S37a}$$

$$k_{C'B} = \frac{k_{CB} p_C}{p_C + p_D}, \tag{S37b}$$

$$\alpha_{C'} = \frac{p_C \alpha_C + p_D \alpha_D}{p_C + p_D}, \tag{S37c}$$

$$p_{C'} = p_C + p_D, \tag{S37d}$$

$$M_{C'} = M_C + M_D. \tag{S37e}$$

Four of the six transition rate coefficients in the reduced equivalent scheme are not influenced by the appended state. As expected, only the transition rate coefficients out of the replacement state $C'$ (i.e., $k_{C'A}$ and $k_{C'B}$) are modified.

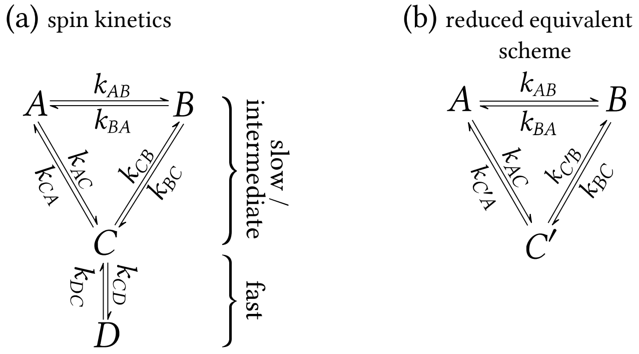


**Figure S12.** Three-state exchange with appended fast-exchanging fourth state. (**a**) Spin kinetics scheme and (**b**) the corresponding reduced equivalent scheme.

Modified transition rate coefficients $k_{C'A}$ and $k_{C'B}$ can also be determined without the use of analytical lineshapes using the

kinetics differential equations (Eq. (4)). In this case, the equations are as follows

$$\frac{\mathrm{d}p_A}{\mathrm{d}t} = -(k_{AB}+k_{AC})p_A + k_{BA}p_B + k_{CA}p_C\,, \tag{S38a}$$

$$\frac{\mathrm{d}p_B}{\mathrm{d}t} = -(k_{BA}+k_{BC})p_B + k_{AB}p_A + k_{CB}p_C\,, \tag{S38b}$$

$$\frac{\mathrm{d}p_C}{\mathrm{d}t} = -(k_{CA}+k_{CB}+k_{CD})p_C + k_{AC}p_A + k_{BC}p_B + k_{DC}p_D\,, \tag{S38c}$$

$$\frac{\mathrm{d}p_D}{\mathrm{d}t} = -k_{DC}p_D + k_{CD}p_C\,. \tag{S38d}$$

After adding Eq. (S38c) and Eq. (S38d), expanding $p_C = \frac{p_C}{p_C+p_D}(p_C+p_D)$ and substituting $p_C + p_D = p_{C'}$ we obtain

$$\frac{\mathrm{d}p_A}{\mathrm{d}t} = -(k_{AB}+k_{AC})p_A + k_{BA}p_B + \frac{k_{CA}p_C}{p_C+p_D}p_{C'}\,, \tag{S39a}$$

$$\frac{\mathrm{d}p_B}{\mathrm{d}t} = -(k_{BA}+k_{BC})p_B + k_{AB}p_A + \frac{k_{CB}p_C}{p_C+p_D}p_{C'}\,, \tag{S39b}$$

$$\frac{\mathrm{d}p_{C'}}{\mathrm{d}t} = -\frac{k_{CA}p_C}{p_C+p_D}p_{C'} - \frac{k_{CB}p_C}{p_C+p_D}p_{C'} + k_{AC}p_A + k_{BC}p_B\,. \tag{S39c}$$

Prefactors of $p_{C'}$ in the above equations yield the correct form of $k_{C'A}$ and $k_{C'B}$ (cf. Eq. (S37a,b)).

## S11 Host–guest binding models

### S11.1 1:1 host–guest binding

Host–guest binding in 1:1 stoichiometry, described by a binding constant $K_{\mathbf{HG}}$, is shown in Fig. S13.

$$\mathbf{H} + \mathbf{G} \xrightleftharpoons{K_{\mathbf{HG}}} \mathbf{HG}$$

**Figure S13.** Scheme of 1:1 host–guest binding model.

Equilibrium in this model is governed by the following equations ($[\bullet]_\mathrm{t}$ stands for total concentration)

$$K_{\mathbf{HG}} = \frac{[\mathbf{HG}]}{[\mathbf{H}][\mathbf{G}]}, \tag{S40a}$$

$$[\mathbf{H}]_\mathrm{t} = [\mathbf{H}] + [\mathbf{HG}], \tag{S40b}$$

$$[\mathbf{G}]_\mathrm{t} = [\mathbf{G}] + [\mathbf{HG}]. \tag{S40c}$$

The concentrations can be calculated analytically in this case as

$$[\mathbf{H}] = \frac{K_{\mathbf{HG}}([\mathbf{H}]_\mathrm{t} - [\mathbf{G}]_\mathrm{t}) - 1 + \sqrt{[K_{\mathbf{HG}}([\mathbf{H}]_\mathrm{t} - [\mathbf{G}]_\mathrm{t}) - 1]^2 + 4K_{\mathbf{HG}}[\mathbf{H}]_\mathrm{t}}}{2K_{\mathbf{HG}}}, \tag{S41a}$$

$$[\mathbf{HG}] = \frac{K_{\mathbf{HG}}([\mathbf{H}]_\mathrm{t} + [\mathbf{G}]_\mathrm{t}) + 1 - \sqrt{[K_{\mathbf{HG}}([\mathbf{H}]_\mathrm{t} - [\mathbf{G}]_\mathrm{t}) - 1]^2 + 4K_{\mathbf{HG}}[\mathbf{H}]_\mathrm{t}}}{2K_{\mathbf{HG}}}, \tag{S41b}$$

$$[\mathbf{G}] = \frac{K_{\mathbf{HG}}([\mathbf{G}]_\mathrm{t} - [\mathbf{H}]_\mathrm{t}) - 1 + \sqrt{[K_{\mathbf{HG}}([\mathbf{H}]_\mathrm{t} - [\mathbf{G}]_\mathrm{t}) - 1]^2 + 4K_{\mathbf{HG}}[\mathbf{H}]_\mathrm{t}}}{2K_{\mathbf{HG}}}. \tag{S41c}$$

### S11.2 Two-state chemical exchange and 1:1 host–guest binding

Correspondence between chemical forms of host and states of a nuclear spin located at the host molecule is illustrated in Fig. S14a,b.

(a) chemical kinetics

$$\mathbf{H} + \mathbf{G} \underset{\kappa_{BA}}{\overset{\kappa_{AB}}{\rightleftharpoons}} \mathbf{HG}$$

(b) spin kinetics

$$A \underset{k_{BA}}{\overset{k_{AB}}{\rightleftharpoons}} B$$

**Figure S14.** Two-state exchange with 1:1 host–guest binding. Schemes for (**a**) chemical kinetics and (**b**) corresponding spin kinetics of a nucleus located at the host molecule.

The binding constant is related to the reaction rate coefficients by the formula $K_{\mathbf{HG}} = \kappa_{AB}/\kappa_{BA}$. As discussed in the main manuscript (Sec. "Chemical exchange in host–guest complexes"), parameters describing chemical kinetics and spin kinetics are related as follows

$$k_{AB} = \kappa_{AB}[\mathbf{G}], \tag{S42a}$$

$$k_{BA} = \kappa_{BA}. \tag{S42b}$$

Figure S15 illustrates a simulated titration of a host with a guest, which form a complex according to Fig. S14a. Simulated spectra shown in Fig. S15a contain two resonances in an intermediate exchange regime. The resonances belong to the host in states $A$ (corresponding to $\mathbf{H}$) and $B$ (corresponding to $\mathbf{HG}$). As the total amount of guest ($[\mathbf{G}]_\mathrm{t}$) increases from zero to one equivalent (from bottom to the top in Fig. S15a), the population of state $B$ increases (Fig. S15b) as well as the concentration of free and bound guest ($[\mathbf{G}]$ and $[\mathbf{HG}]$ in Fig. S15c). Because the exchange is in an intermediate regime, the apparent peak maxima shift towards each other and eventually coalesce (Fig. S15d, details about two-state exchange coalescence are given in Sec. S5.2). In accordance with Eq. (S42) the transition rate coefficient $k_{AB}$ depends on guest concentration, while $k_{BA}$ does not. Note that although the function $k_{AB}([\mathbf{G}])$ is linear, the function $k_{AB}([\mathbf{G}]_\mathrm{t})$ is nonlinear and can be calculated using Eqs. (S42a) and (S41c).

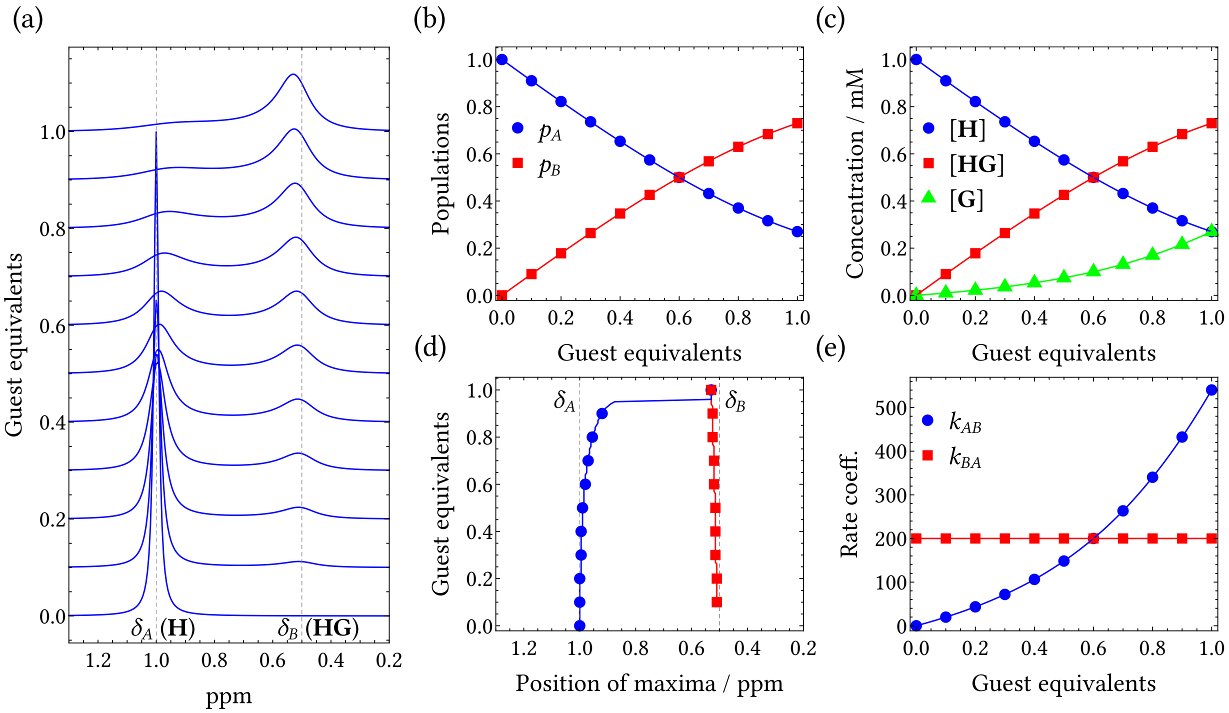


**Figure S15.** Illustration of two-state exchange combined with 1:1 host–guest binding in a simulated titration of host with guest (at constant $[\mathbf{H}]_t$). (**a**) Simulated spectral lineshapes ($y$-scaling of spectra is adjusted for clarity). Concentration of guest corresponds to the value where the simulated spectrum meets the $y$-axis. The spectrum of the free host is cropped for clarity. Concentration dependence of (**b**) populations, (**c**) concentration of all species, (**d**) positions of peak maxima, (**e**) transition rate coefficients. Discrete points in plots of various quantities correspond to the spectra shown on the left. Simulation parameters: $R_2^A = R_2^B = 30\ \mathrm{s}^{-1}$, $\kappa_{AB} = 2\times10^6\ \mathrm{s}^{-1}\mathrm{M}^{-2}$, $K_{\mathbf{HG}} = 1\times10^4\ \mathrm{M}^{-1}$, $[\mathbf{H}]_t = 1$ mM, spectrometer frequency $\nu_0 = 500.13$ MHz (which is used for conversion from ppm to rad.s$^{-1}$). Chemical shifts of pure $A$ (corresponding to **H**) and pure $B$ (**HG**) states are $\delta_A = 1$ ppm and $\delta_B = 0.5$ ppm, respectively. Equivalents of guest are defined as $[\mathbf{G}]_t/[\mathbf{H}]_t$. The spectral lineshapes were calculated using Eq. (7) and concentrations determined using Eq. (S41).

### S11.3 Competitive host–ligand binding

Competitive binding is a situation where two different ligands bind to a single binding site at the host molecule (and the binding of one ligand prevents binding of another ligand). If the first ligand is denoted as **G** (guest) and the second competitive ligand as **W** (in this case it is water), the binding can be described by two binding constants $K_{\mathbf{HG}}$ and $K_{\mathbf{HW}}$ according to the scheme shown in Fig. S16. We assume the existence of a single binding site in the host molecule (1:1 stoichiometry).

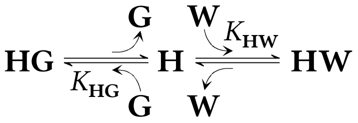


**Figure S16.** Scheme of host–ligand competitive binding model.

Simple model of competitive host–guest–water binding is given in the report by Labuta et al.[S25] The governing equations

$$K_{\mathbf{HG}} = \frac{[\mathbf{HG}]}{[\mathbf{H}][\mathbf{G}]}, \tag{S43a}$$

$$K_{\mathbf{HW}} = \frac{[\mathbf{HW}]}{[\mathbf{H}][\mathbf{W}]}, \tag{S43b}$$

$$[\mathbf{H}]_t = [\mathbf{H}] + [\mathbf{HG}] + [\mathbf{HW}], \tag{S43c}$$

$$[\mathbf{G}]_t = [\mathbf{G}] + [\mathbf{HG}], \tag{S43d}$$

$$[\mathbf{W}]_t = [\mathbf{W}] + [\mathbf{HW}] \tag{S43e}$$

can be solved either analytically[S25] or numerically. Here, we describe the numerical solution and follow the approach given in the paper of Hargrove,[S26] where the concentrations of host-related species are expressed as functions of [**G**] and [**W**] instead of $[\mathbf{G}]_t$ and $[\mathbf{W}]_t$. Then we obtain simple expressions for free host, host–guest and host–water complex, respectively, in the following forms

$$[\mathbf{H}] = \frac{[\mathbf{H}]_t}{1 + K_{\mathbf{HG}}[\mathbf{G}] + K_{\mathbf{HW}}[\mathbf{W}]}, \tag{S44a}$$

$$[\mathbf{HG}] = \frac{K_{\mathbf{HG}}[\mathbf{G}][\mathbf{H}]_t}{1 + K_{\mathbf{HG}}[\mathbf{G}] + K_{\mathbf{HW}}[\mathbf{W}]}, \tag{S44b}$$

$$[\mathbf{HW}] = \frac{K_{\mathbf{HW}}[\mathbf{W}][\mathbf{H}]_t}{1 + K_{\mathbf{HG}}[\mathbf{G}] + K_{\mathbf{HW}}[\mathbf{W}]}. \tag{S44c}$$

A numerical method is then applied to find the concentrations [**G**] and [**W**] as functions of $[\mathbf{G}]_t$ and $[\mathbf{W}]_t$ by solving the following system of equations

$$0 = [\mathbf{G}] - [\mathbf{G}]_t + [\mathbf{G}]^2 K_{\mathbf{HG}} - [\mathbf{G}][\mathbf{G}]_t K_{\mathbf{HG}} + [\mathbf{G}][\mathbf{H}]_t K_{\mathbf{HG}} + [\mathbf{G}]K_{\mathbf{HW}}[\mathbf{W}] - [\mathbf{G}]_t K_{\mathbf{HW}}[\mathbf{W}], \tag{S45a}$$

$$0 = [\mathbf{W}] + [\mathbf{G}]K_{\mathbf{HG}}[\mathbf{W}] + [\mathbf{H}]_t K_{\mathbf{HW}}[\mathbf{W}] + K_{\mathbf{HW}}[\mathbf{W}]^2 - [\mathbf{W}]_t - [\mathbf{G}]K_{\mathbf{HG}}[\mathbf{W}]_t - K_{\mathbf{HW}}[\mathbf{W}][\mathbf{W}]_t. \tag{S45b}$$

### S11.4 Three-state chemical exchange and competitive host–ligand binding

From the spectrum of the host molecule, the species **HG**, **H** and **HW** can be identified as spin states $A$, $B$ and $C$, respectively, see Fig. S17a,b.

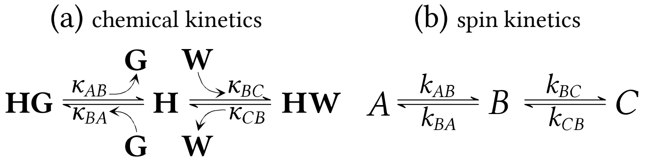


**Figure S17.** Consecutive three-state exchange in 1:1 host–ligand competitive binding. Schemes for (**a**) chemical kinetics and (**b**) corresponding spin kinetics of a nucleus located at the host molecule.

The binding constants are related to the reaction rate coefficients by the formulae $K_{\mathbf{HG}} = \kappa_{BA}/\kappa_{AB}$ and $K_{\mathbf{HW}} = \kappa_{BC}/\kappa_{CB}$. Similarly to the example shown in Sec. "Chemical exchange in host–guest complexes" in the main manuscript, after a comparison of equations for forward and backward reaction and transition rates (in Fig. S17a,b), we obtain the following relationships

$$k_{AB} = \kappa_{AB}, \tag{S46a}$$

$$k_{BA} = \kappa_{BA}[\mathbf{G}], \tag{S46b}$$

$$k_{BC} = \kappa_{BC}[\mathbf{W}], \tag{S46c}$$

$$k_{CB} = \kappa_{CB}. \tag{S46d}$$

Figure S18 illustrates a simulated titration of a host with a guest in the presence of water ($[\mathbf{H}]_t$ and $[\mathbf{W}]_t$ are kept constant), the host forms a complex with both ligands according to Fig. S17a. Simulated spectra in Fig. S18a contain three resonances in an intermediate exchange regime. The resonances belong to the host in states $A$ (corresponding to **HG**), $B$ (**H**) and $C$ (**HW**). In the absence of guest (bottom spectrum in Fig. S18a), host molecules are either in a free state or are complexed with water. When the total quantity of guest ($[\mathbf{G}]_t$) increases, the amount of host–guest complex increases, while the host–water complex is depopulated due to competition with the guest (Fig. S18b,c). Being in the intermediate exchange regime, all peaks are broadened. The apparent maxima of peaks corresponding to states $A$ and $B$ shift towards each other and eventually coalesce (Fig. S18d) due to an increase of the $k_{BA}$ transition rate coefficient. In accordance with Eqs. (S46a,d), the rate coefficients $k_{AB}$ and $k_{CB}$ do not depend on concentration, while $k_{BA}$ and $k_{BC}$ depend nonlinearly on $[\mathbf{G}]_t$ (Eqs. (S46b,c) and (S45)).

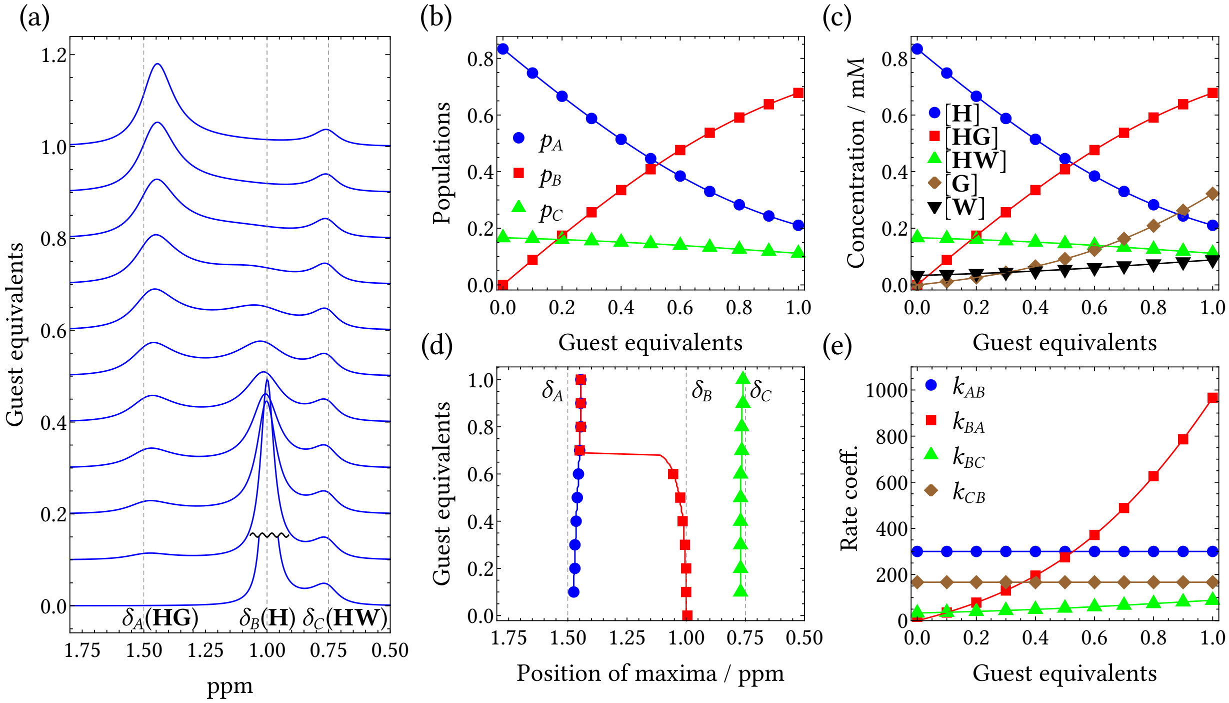


**Figure S18.** Illustration of three-state exchange combined with host–ligand competitive binding in a simulated titration of host with guest (at constant $[\mathbf{H}]_\mathrm{t}$ and $[\mathbf{W}]_\mathrm{t}$). (**a**) Simulated spectral lineshapes ($y$-scaling of spectra is adjusted for clarity). Concentration of guest corresponds to the value where the simulated spectrum meets the $y$-axis. Concentration dependence of (**b**) populations, (**c**) concentration of all species, (**d**) positions of peak maxima, (**e**) transition rate coefficients. Discrete points in plots of various quantities correspond to the spectra shown on the left. Simulation parameters: $R_2^A = R_2^B = R_2^C = 30\ \mathrm{s}^{-1}$, $\kappa_{BA} = 3\times10^6\ \mathrm{s}^{-1}\mathrm{M}^{-2}$, $\kappa_{BC} = 1\times10^6\ \mathrm{s}^{-1}\mathrm{M}^{-2}$, $K_{\mathbf{HG}} = 1\times10^4\ \mathrm{M}^{-1}$, $K_{\mathbf{HW}} = 6000\ \mathrm{M}^{-1}$, $[\mathbf{H}]_\mathrm{t} = 1$ mM, $[\mathbf{W}]_\mathrm{t} = 0.2$ mM, spectrometer frequency $\nu_0 = 500.13$ MHz (which is used for conversion from ppm to rad.s$^{-1}$). Chemical shifts of pure $A$ (corresponding to **HG**), pure $B$ (**H**) and pure $C$ (**HW**) states are $\delta_A = 1.5$ ppm, $\delta_B = 1$ ppm and $\delta_C = 0.75$ ppm, respectively. Equivalents of guest are defined as $[\mathbf{G}]_\mathrm{t}/[\mathbf{H}]_\mathrm{t}$. The spectral lineshapes were calculated using Eq. (13) and concentrations determined using Eqs. (S44) and (S45).

## S11.5 Kinetics of the di-bromobenzylated oxoporphyrinogen system

Our model for the system of di-bromobenzylated oxoporphyrinogen (**H**) in the presence of ($R$)-camphorsulfonic acid (**G**) and water (**W**) combines the competitive host–ligand binding scheme for chemical kinetics (described in the previous section) and the half-symmetric three-state exchange model for the observed spin kinetics. The spin kinetics corresponds to the central NH nuclear spins located at the host.

There are multiple levels of description of the molecular kinetics in our system. The most elementary description is a simplified chemical kinetics scheme in Fig. S19a, assuming the host molecule in three possible chemical states, **H**, **HG** and **HW**. The simplified chemical kinetics is governed by the following equations

$$\frac{\mathrm{d}[\mathbf{HG}]}{\mathrm{d}t} = -\kappa_{AC}[\mathbf{HG}] + 4\kappa_{CA}[\mathbf{G}][\mathbf{H}], \tag{S47a}$$

$$\frac{\mathrm{d}[\mathbf{H}]}{\mathrm{d}t} = -4\kappa_{CA}[\mathbf{G}][\mathbf{H}] - \kappa_{CD}[\mathbf{W}][\mathbf{H}] + \kappa_{AC}[\mathbf{HG}] + \kappa_{DC}[\mathbf{HW}], \tag{S47b}$$

$$\frac{\mathrm{d}[\mathbf{HW}]}{\mathrm{d}t} = -\kappa_{DC}[\mathbf{HW}] + \kappa_{CD}[\mathbf{W}][\mathbf{H}]. \tag{S47c}$$

The factor of four in the term $4\kappa_{CA}[\mathbf{G}][\mathbf{H}]$ is caused by the presence of four possible protonation sites, increasing the rate of **HG** formation. The reaction rate coefficients are related to the equilibrium constants through the following formulae (obtained

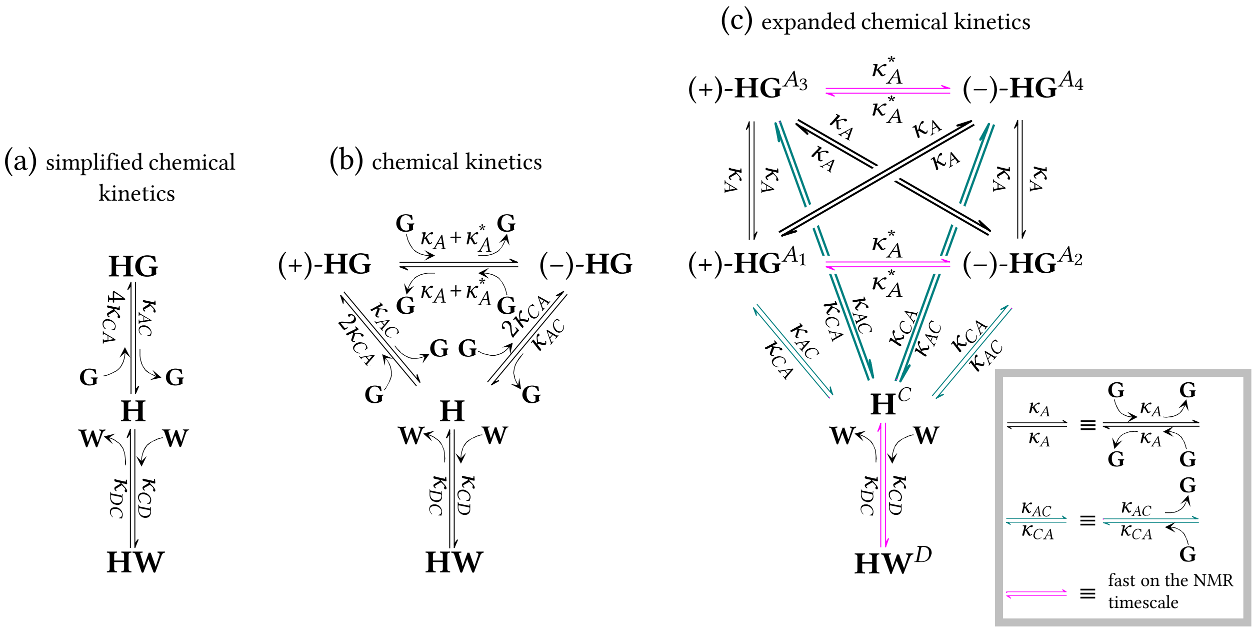


**Figure S19.** Chemical kinetics schemes for the multi-state system of di-bromobenzylated oxoporphyrinogen (host **H**) in the presence of two potential ligands, (*R*)-camphorsulfonic acid (ligand **G**) and water (ligand **W**). (**a**) Simplified chemical kinetics scheme corresponding to 1:1 **H**:**G** binding with competitive 1:1 **H**:**W** binding described by Eqs. (S47). (**b**) Chemical kinetics scheme described by Eqs. (S50). (**c**) Expanded chemical kinetics scheme described by Eqs. (S51). All relevant molecular processes and their reaction rate coefficients are shown. Processes denoted by magenta arrows are fast on the NMR timescale.

for the time derivatives in Eqs. (S47a) and (S47c) equal to zero)

$$K_{\mathbf{HG}} = 4K_{\mathbf{HG}}^{\text{micro}} = 4\frac{\kappa_{CA}}{\kappa_{AC}}, \tag{S48a}$$

$$K_{\mathbf{HW}} = \frac{\kappa_{CD}}{\kappa_{DC}}. \tag{S48b}$$

The stepwise binding constant, defined as $K_{\mathbf{HG}} = [\mathbf{HG}]/([\mathbf{H}][\mathbf{G}])$, describes protonation of any of the four possible protonation sites (hence the factor of four in Eq. (S48a)). On the other hand, the microscopic binding constant $K_{\mathbf{HG}}^{\text{micro}} = \kappa_{CA}/\kappa_{AC}$ describes binding to one particular binding site.[S27] Thus, this constant corresponds to the "microscopic" standard reaction Gibbs energy $\Delta G_{\mathbf{HG}}^{\circ\text{micro}}$, which can be obtained from quantum chemistry calculations[S28] as $\Delta G_{\mathbf{HG}}^{\circ\text{micro}} = \mu_{\mathbf{HG}}^{\circ} - \mu_{\mathbf{H}}^{\circ} - \mu_{\mathbf{G}}^{\circ}$ ($\mu_{\bullet}^{\circ}$ represents the standard chemical potential of species indicated in subscript). The quantity $\Delta G_{\mathbf{HG}}^{\circ\text{micro}}$ is higher than the standard reaction Gibbs energy $\Delta G_{\mathbf{HG}}^{\circ} = -RT\ln K_{\mathbf{HG}}$ (the quantity $\Delta G_{\mathbf{HG}}^{\circ}$ is always defined by the latter equation from the stepwise equilibrium constant, but it should be correctly interpreted[S29,S30]),

$$\Delta G_{\mathbf{HG}}^{\text{omicro}} = -RT\ln K_{\mathbf{HG}}^{\text{micro}} = -RT\ln\frac{K_{\mathbf{HG}}}{4} = \Delta G_{\mathbf{HG}} + RT\ln 4. \tag{S49}$$

The numerical value is $\Delta G_{\mathbf{HG}}^{\text{omicro}} = \Delta G_{\mathbf{HG}} + RT\ln 4 = -27.76 + 3.43\ \text{kJ.mol}^{-1} = -24.33\ \text{kJ.mol}^{-1}$ at $T = 298$ K.

In fact, the protonated species **HG** are not identical as they form two enantiomers (+)-**HG** and (–)-**HG**. The chemical kinetics is described by the scheme in Fig. S19b, which is an expansion of the scheme in Fig. S19a. This scheme is governed by the following equations

$$\frac{\mathrm{d}[(+)\text{-}\mathbf{HG}]}{\mathrm{d}t} = -(\kappa_A + \kappa_A^*)[\mathbf{G}][(+)\text{-}\mathbf{HG}] - \kappa_{AC}[(+)\text{-}\mathbf{HG}] + (\kappa_A + \kappa_A^*)[\mathbf{G}][(-)\text{-}\mathbf{HG}] + 2\kappa_{CA}[\mathbf{G}][\mathbf{H}], \tag{S50a}$$

$$\frac{\mathrm{d}[(-)\text{-}\mathbf{HG}]}{\mathrm{d}t} = -(\kappa_A + \kappa_A^*)[\mathbf{G}][(-)\text{-}\mathbf{HG}] - \kappa_{AC}[(-)\text{-}\mathbf{HG}] + (\kappa_A + \kappa_A^*)[\mathbf{G}][(+)\text{-}\mathbf{HG}] + 2\kappa_{CA}[\mathbf{G}][\mathbf{H}], \tag{S50b}$$

$$\frac{\mathrm{d}[\mathbf{H}]}{\mathrm{d}t} = -4\kappa_{CA}[\mathbf{G}][\mathbf{H}] - \kappa_{CD}[\mathbf{W}][\mathbf{H}] + \kappa_{AC}([(+)\text{-}\mathbf{HG}] + [(-)\text{-}\mathbf{HG}]) + \kappa_{DC}[\mathbf{HW}], \tag{S50c}$$

$$\frac{\mathrm{d}[\mathbf{HW}]}{\mathrm{d}t} = -\kappa_{DC}[\mathbf{HW}] + \kappa_{CD}[\mathbf{W}][\mathbf{H}]. \tag{S50d}$$

These equations are compatible with the simplified chemical kinetics (Eqs. (S47)) by adding Eqs. (S50a) and (S50b), and substituting [(+)-**HG**] + [(–)-**HG**] = [**HG**].

In order to establish the connection between chemical kinetics and spin kinetics, the scheme in Fig. S19b must be expanded to incorporate all relevant microstates, see Fig. S19c. The states (+)-$\mathbf{HG}^{A_1}$, (+)-$\mathbf{HG}^{A_3}$, (–)-$\mathbf{HG}^{A_2}$ and (–)-$\mathbf{HG}^{A_4}$ correspond to protonation of different C=O sites (see Fig. 6 for molecular structures). The expanded chemical kinetics is described by the following equations

$$\begin{aligned}
\frac{\mathrm{d}[(+)\text{-}\mathbf{HG}^{A_1}]}{\mathrm{d}t} &= -(2\kappa_A + \kappa_A^*)[\mathbf{G}][(+)\text{-}\mathbf{HG}^{A_1}] - \kappa_{AC}[(+)\text{-}\mathbf{HG}^{A_1}] \\
&\quad + \kappa_A[\mathbf{G}][(+)\text{-}\mathbf{HG}^{A_3}] + \kappa_A^*[\mathbf{G}][(-)\text{-}\mathbf{HG}^{A_2}] + \kappa_A[\mathbf{G}][(-)\text{-}\mathbf{HG}^{A_4}] + \kappa_{CA}[\mathbf{G}][\mathbf{H}^C], &\text{(S51a)} \\
\frac{\mathrm{d}[(+)\text{-}\mathbf{HG}^{A_3}]}{\mathrm{d}t} &= -(2\kappa_A + \kappa_A^*)[\mathbf{G}][(+)\text{-}\mathbf{HG}^{A_3}] - \kappa_{AC}[(+)\text{-}\mathbf{HG}^{A_3}] \\
&\quad + \kappa_A[\mathbf{G}][(+)\text{-}\mathbf{HG}^{A_1}] + \kappa_A[\mathbf{G}][(-)\text{-}\mathbf{HG}^{A_2}] + \kappa_A^*[\mathbf{G}][(-)\text{-}\mathbf{HG}^{A_4}] + \kappa_{CA}[\mathbf{G}][\mathbf{H}^C], &\text{(S51b)} \\
\frac{\mathrm{d}[(-)\text{-}\mathbf{HG}^{A_2}]}{\mathrm{d}t} &= -(2\kappa_A + \kappa_A^*)[\mathbf{G}][(-)\text{-}\mathbf{HG}^{A_2}] - \kappa_{AC}[(-)\text{-}\mathbf{HG}^{A_2}] \\
&\quad + \kappa_A^*[\mathbf{G}][(+)\text{-}\mathbf{HG}^{A_1}] + \kappa_A[\mathbf{G}][(+)\text{-}\mathbf{HG}^{A_3}] + \kappa_A[\mathbf{G}][(-)\text{-}\mathbf{HG}^{A_4}] + \kappa_{CA}[\mathbf{G}][\mathbf{H}^C], &\text{(S51c)} \\
\frac{\mathrm{d}[(-)\text{-}\mathbf{HG}^{A_4}]}{\mathrm{d}t} &= -(2\kappa_A + \kappa_A^*)[\mathbf{G}][(-)\text{-}\mathbf{HG}^{A_4}] - \kappa_{AC}[(-)\text{-}\mathbf{HG}^{A_4}] \\
&\quad + \kappa_A[\mathbf{G}][(+)\text{-}\mathbf{HG}^{A_1}] + \kappa_A^*[\mathbf{G}][(+)\text{-}\mathbf{HG}^{A_3}] + \kappa_A[\mathbf{G}][(-)\text{-}\mathbf{HG}^{A_2}] + \kappa_{CA}[\mathbf{G}][\mathbf{H}^C], &\text{(S51d)} \\
\frac{\mathrm{d}[\mathbf{H}^C]}{\mathrm{d}t} &= -4\kappa_{CA}[\mathbf{G}][\mathbf{H}^C] - \kappa_{CD}[\mathbf{W}][\mathbf{H}^C] \\
&\quad + \kappa_{AC}\left([(+)\text{-}\mathbf{HG}^{A_1}] + [(+)\text{-}\mathbf{HG}^{A_3}] + [(-)\text{-}\mathbf{HG}^{A_2}] + [(-)\text{-}\mathbf{HG}^{A_4}]\right) + \kappa_{DC}[\mathbf{HW}^D], &\text{(S51e)} \\
\frac{\mathrm{d}[\mathbf{HW}^D]}{\mathrm{d}t} &= -\kappa_{DC}[\mathbf{HW}^D] + \kappa_{CD}[\mathbf{W}][\mathbf{H}^C]. &\text{(S51f)}
\end{aligned}$$

The above equations are compatible with the chemical kinetics scheme governed by Eqs. (S50) as shown by summing Eq. (S51a) with Eq. (S51b) and Eq. (S51c) with Eq. (S51d), and substituting [(+)-$\mathbf{HG}^{A_1}$] + [(+)-$\mathbf{HG}^{A_3}$] = [(+)-**HG**] and [(–)-$\mathbf{HG}^{A_2}$] + [(–)-$\mathbf{HG}^{A_4}$] = [(–)-**HG**].

This expanded chemical kinetics scheme accounts for all relevant molecular processes and microstates (i.e., there is absence of integer prefactors or sums of reaction rate coefficients accounting for degeneracy) and explains all the prefactors or sums of reaction rate coefficients used in previous schemes in Fig. S19a,b. The corresponding energy barriers can be calculated employing the Eyring equation (Eq. 23). In order to simplify the model, the guest-mediated prototropic tautomerization between states (+)-$\mathbf{HG}^{A_1}$ ↔ (+)-$\mathbf{HG}^{A_3}$, (+)-$\mathbf{HG}^{A_1}$ ↔ (–)-$\mathbf{HG}^{A_4}$, (–)-$\mathbf{HG}^{A_2}$ ↔ (+)-$\mathbf{HG}^{A_3}$ and (–)-$\mathbf{HG}^{A_2}$ ↔ (–)-$\mathbf{HG}^{A_4}$ is characterized by single effective reaction rate coefficient $\kappa_A$, although the real energy barriers might differ. On the other hand, prototropic tautomerization between states (+)-$\mathbf{HG}^{A_1}$ ↔ (–)-$\mathbf{HG}^{A_2}$ and (+)-$\mathbf{HG}^{A_3}$ ↔ (–)-$\mathbf{HG}^{A_4}$ is characterized by the reaction rate coefficient $\kappa_A^*$.

At this point, fast exchange between states (+)-$\mathbf{HG}^{A_1}$ ↔ (–)-$\mathbf{HG}^{A_2}$, (+)-$\mathbf{HG}^{A_3}$ ↔ (–)-$\mathbf{HG}^{A_4}$ and $\mathbf{H}^C$ ↔ $\mathbf{H}^D$ should be taken into account. The equivalent reduced spin kinetics scheme is constructed from the expanded chemical kinetics scheme in Fig. S19c according to the procedure explained in Sec. S10. In this case, Eqs. (S51a) and (S51c), Eqs. (S51b) and (S51d), and Eqs. (S51e) and (S51f), respectively, are added up, expansion $[\mathbf{H}^C] = \frac{[\mathbf{H}^C]}{[\mathbf{H}^C]+[\mathbf{H}^D]}([\mathbf{H}^C] + [\mathbf{H}^D])$ is made and substitutions [(+)-$\mathbf{HG}^{A_1}$] + [(–)-$\mathbf{HG}^{A_2}$] = $[\mathbf{HG}^{A'_{\mathrm{I}}}]$, [(+)-$\mathbf{HG}^{A_3}$] + [(–)-$\mathbf{HG}^{A_4}$] = $[\mathbf{HG}^{A'_{\mathrm{II}}}]$ and $[\mathbf{H}^C] + [\mathbf{H}^D] = [\mathbf{H}^{C'}]$ are used. This procedure yields governing equations for three-state spin kinetics corresponding to the spin kinetics scheme in Fig. S20a, in particular

$$\begin{aligned}
\frac{\mathrm{d}[\mathbf{HG}^{A'_{\mathrm{I}}}]}{\mathrm{d}t} &= -2\kappa_A[\mathbf{G}][\mathbf{HG}^{A'_{\mathrm{I}}}] - \kappa_{AC}[\mathbf{HG}^{A'_{\mathrm{I}}}] + 2\kappa_A[\mathbf{G}][\mathbf{HG}^{A'_{\mathrm{II}}}] + 2\kappa_{CA}[\mathbf{G}][\mathbf{H}^C], &\text{(S52a)} \\
\frac{\mathrm{d}[\mathbf{HG}^{A'_{\mathrm{II}}}]}{\mathrm{d}t} &= -2\kappa_A[\mathbf{G}][\mathbf{HG}^{A'_{\mathrm{II}}}] - \kappa_{AC}[\mathbf{HG}^{A'_{\mathrm{II}}}] + 2\kappa_A[\mathbf{G}][\mathbf{HG}^{A'_{\mathrm{I}}}] + 2\kappa_{CA}[\mathbf{G}][\mathbf{H}^C], &\text{(S52b)} \\
\frac{\mathrm{d}[\mathbf{H}^{C'}]}{\mathrm{d}t} &= -4\kappa_{CA}\frac{[\mathbf{G}][\mathbf{H}^C]}{[\mathbf{H}^C] + [\mathbf{H}^D]}[\mathbf{H}^{C'}] + \kappa_{AC}\left([\mathbf{HG}^{A'_{\mathrm{I}}}] + [\mathbf{HG}^{A'_{\mathrm{II}}}]\right). &\text{(S52c)}
\end{aligned}$$

These equations can be further compared with a spin kinetics scheme formulated in terms of transition rate coefficients as

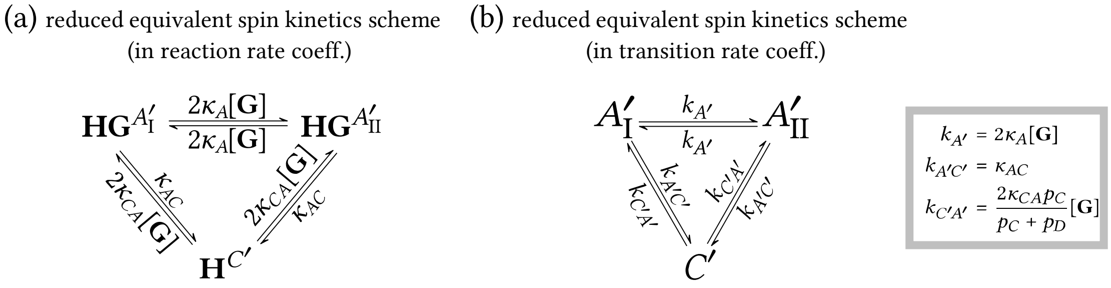


**Figure S20.** Reduced equivalent spin kinetics schemes for the multi-state system of di-bromobenzylated oxoporphyrinogen (host **H**) in the presence of two potential ligands, (*R*)-camphorsulfonic acid (ligand **G**) and water (ligand **W**). The schemes refer to central NH protons of the host molecule. (**a**) Spin kinetics in terms of reaction rate coefficients as obtained from contraction of the scheme in Fig. S19c. It is described by Eqs. (S52). (**b**) Corresponding spin kinetics in terms of transition rate coefficients described by Eqs. (S53). It has the form of half-symmetric three-state exchange. Comparison with (a) gives the relation between transition and reaction rate coefficients in Eqs. (S54). This model was used for lineshape fitting, see Sec. S12 for details.

shown in Fig. S20b, which is governed by the following set of differential equations

$$\frac{\mathrm{d}p_{A'_\mathrm{I}}}{\mathrm{d}t} = -(k_{A'} + k_{A'C'})p_{A'_\mathrm{I}} + k_{A'}p_{A'_\mathrm{II}} + k_{C'A'}p_{C'}\,, \tag{S53a}$$

$$\frac{\mathrm{d}p_{A'_\mathrm{II}}}{\mathrm{d}t} = -(k_{A'} + k_{A'C'})p_{A'_\mathrm{II}} + k_{A'}p_{A'_\mathrm{I}} + k_{C'A'}p_{C'}\,, \tag{S53b}$$

$$\frac{\mathrm{d}p_{C'}}{\mathrm{d}t} = -2k_{C'A'}p_{C'} + k_{A'C'}p_{A'_\mathrm{I}} + k_{A'C'}p_{A'_\mathrm{II}}\,. \tag{S53c}$$

Relationships between the transition and reaction rate coefficients are established by comparison of Eqs. (S52) and (S53) using the appropriate relations for spin state populations, i.e., $p_{A'_\mathrm{I}} = \frac{[\mathbf{HG}^{A'_\mathrm{I}}]}{[\mathbf{H}]_\mathrm{t}}$, $p_{A'_\mathrm{II}} = \frac{[\mathbf{HG}^{A'_\mathrm{II}}]}{[\mathbf{H}]_\mathrm{t}}$ and $p_{C'} = \frac{[\mathbf{H}^{C'}]}{[\mathbf{H}]_\mathrm{t}}$. As a result, we also obtain the dependencies of transition rate coefficients on guest concentration,

$$k_{A'} = 2\kappa_A[\mathbf{G}]\,, \tag{S54a}$$

$$k_{A'C'} = \kappa_{AC}\,, \tag{S54b}$$

$$k_{C'A'} = \frac{2\kappa_{CA}p_C}{p_C + p_D}[\mathbf{G}]\,. \tag{S54c}$$

### S11.6 Prototropic tautomerization processes

Prototropic tautomerization consists of several steps, which are indistinguishable in NMR (due to fast exchange regime). Here, we suggest the corresponding reaction schemes for tautomerization processes. Initial state (–)-**HG** can undergo the tautomerization process in three different ways as illustrated in Fig. S21 (final state (+)-**HG**, characterized by $\kappa_A^*$), Fig. S22 (final state (–)-**HG**, characterized by $\kappa_A$) and Fig. S23 (final state (+)-**HG**, characterized by $\kappa_A$). All three reaction paths are reversible and consist of analogous steps. In step (1), an incoming guest (red) forms a hydrogen bond with host's carbonyl group thus forming hydrogen-bonded structure (−)-**HG·G**. The next step (2) shows the suggested transition state $(\mathbf{HG}_2)^\ddagger$, which is created by formal redistribution of electron density (denoted by blue arrows in (−)-**HG·G** structure and by dashed bonds in $(\mathbf{HG}_2)^\ddagger$ structure). Formally, partial charge $+\delta$ is located at the central alkylated amines and at the protonated carbonyls although in reality, the charge is delocalized. During this step, guest anions change positions and are located close to partially charged atoms of the host in $(\mathbf{HG}_2)^\ddagger$. This allows for (blue and red) guest anions exchange, as shown in step (3). Hence, prototropic tautomerization can, in principle, proceed in two branches, i.e., the initial guest counteranion (blue) either remains at the porphyrinogen center or it is replaced by the incoming acid anion (red). Both branches lead to hydrogen bonded species (+)-**HG·G** or (−)-**HG·G** in step (4) (redistribution of electron density is denoted by blue arrows in $(\mathbf{HG}_2)^\ddagger$ structure). Finally, monoprotonated host–guest complex (+)-**HG** or (–)-**HG** is formed in step (5). Although the tautomerization processes consist of several steps, in our chemical kinetics scheme (Fig. S19c), they are described by effective barriers $\Delta G_A^\ddagger$ (corresponding to $\kappa_A$) and $\Delta G_{A^*}^\ddagger$ (corresponding to $\kappa_A^*$). Step (1) is a second-order reaction, hence the effective forward reaction rate ($\kappa_A$[**G**][**HG**] or $\kappa_A^*$[**G**][**HG**]) depends linearly on [**G**] and [**HG**]. Similarly, step (5) is also a second-order reaction, hence the effective backward reaction rate ($\kappa_A$[**G**][**HG**] or $\kappa_A^*$[**G**][**HG**]) depends linearly on [**G**] and [**HG**]. Note that we assume equal effective barriers $\Delta G_A^\ddagger$ for protonation transfers "left-to-right" (Fig. S22) and "left-to-down" and (Fig. S23).

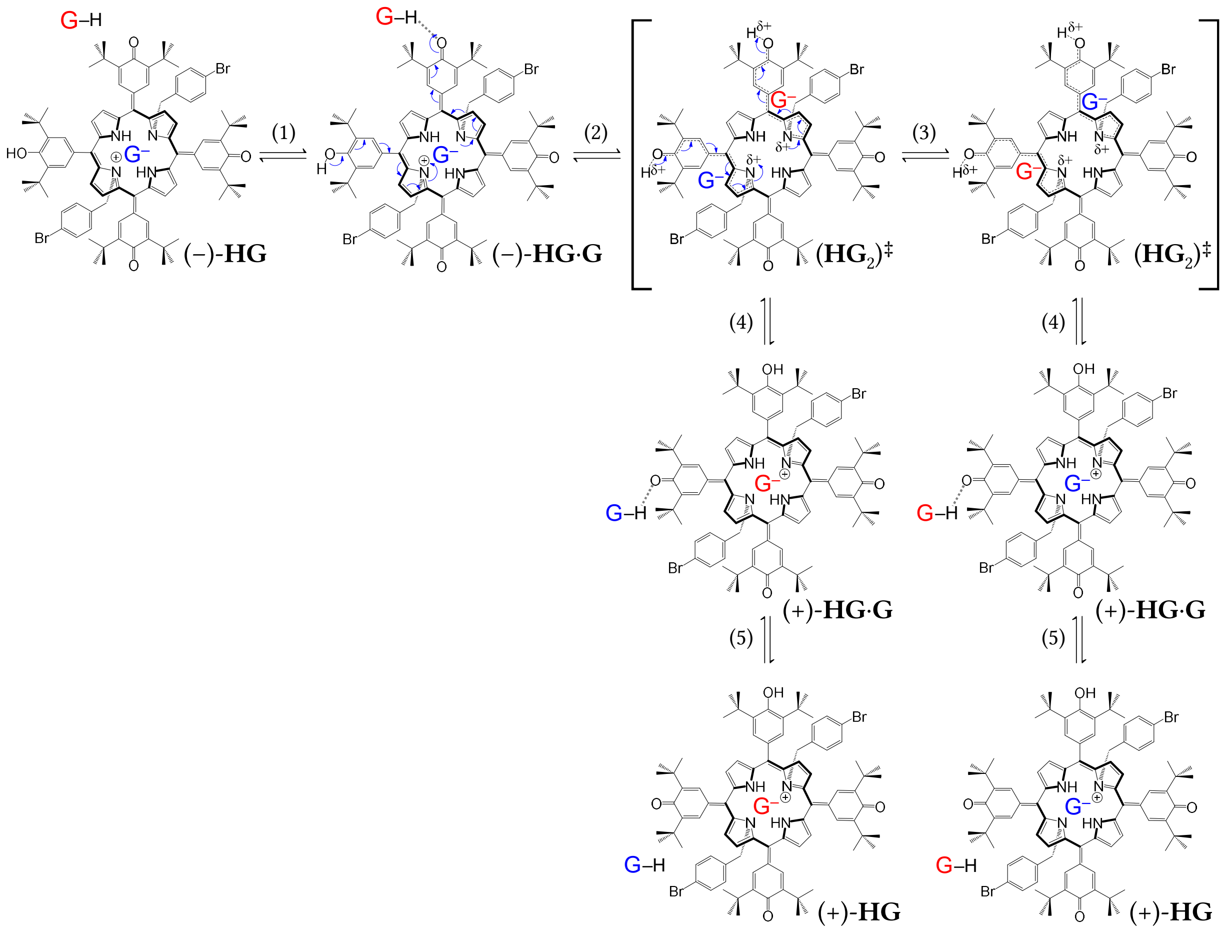


**Figure S21.** Suggested transition structures for prototropic tautomerization from (–)-**HG** to (+)-**HG** characterized by $\kappa_A^*$. Dotted line in (+)-**HG·G** and (−)-**HG·G** represents hydrogen bond, dashed lines in $(\mathbf{HG}_2)^\ddagger$ represent partially formed bonds in transition state.

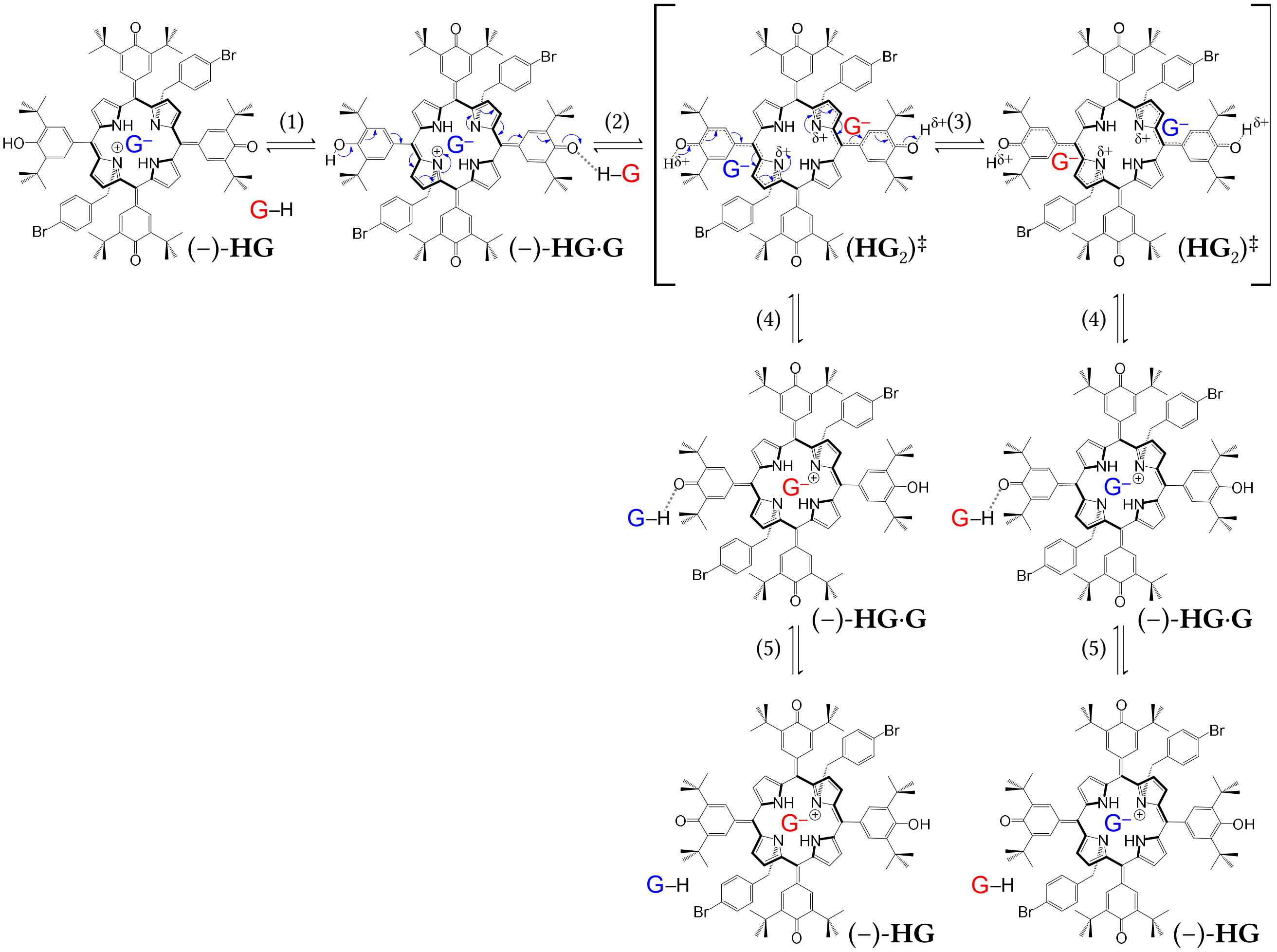


**Figure S22.** Suggested transition structures for prototropic tautomerization from (–)-**HG** to (–)-**HG** characterized by $\kappa_A$. Dotted line in (−)-**HG·G** represents hydrogen bond, dashed lines in (**HG**$_2$)$^‡$ represent partially formed bonds in transition state.

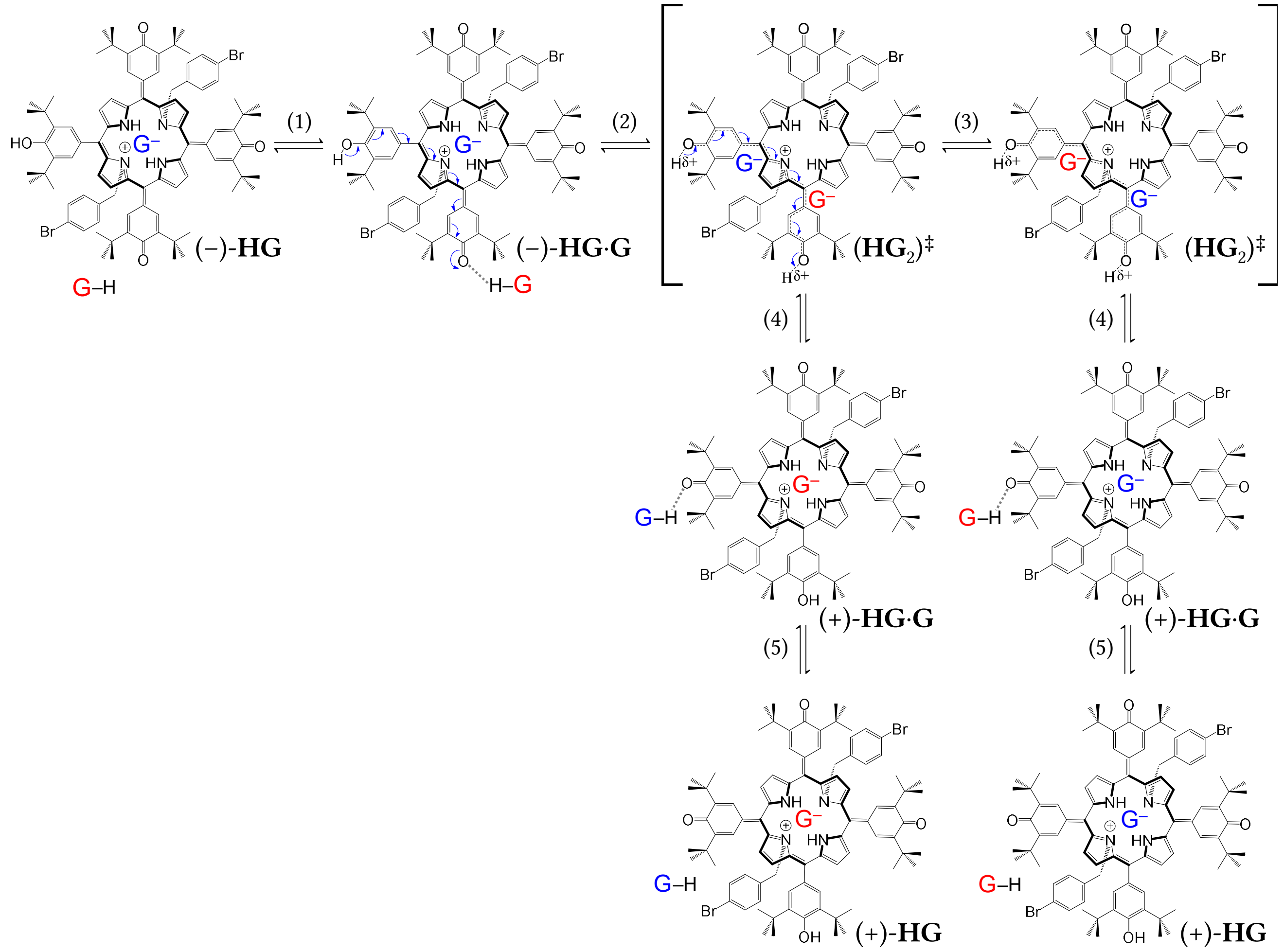


**Figure S23.** Suggested transition structures for prototropic tautomerization from (–)-**HG** to (+)-**HG** characterized by $\kappa_A$. Dotted line in (+)-**HG·G** and (−)-**HG·G** represents hydrogen bond, dashed lines in ($\mathbf{HG}_2$)$^{\ddagger}$ represent partially formed bonds in transition state.

## S12 Fitting of experimental data

The fitting procedure comprises of two consecutive steps:

**Step 1 – Lineshape fitting:**

Fitting of tertiary butyl (TB) resonances of the host, methyl (MET) group resonances of the guest (averaged signal due to fast exchange between **G** and **HG**), and resonance due to water (averaged signal due to fast exchange between **W** and **HW**) with Lorentzian lineshapes was performed in order to provide accurate number of equivalents of guest and water (with respect to host) and consequently their total concentrations. This fitting procedure was realized in *Excel* (with Solver add-in using iterative generalized reduced gradient (GRG) nonlinear method for minimization) as illustrated in Supplementary file `41598_2022_20136_MOESM2_ESM.xlsx`. Total concentration of host was calculated from the weight of the sample in powder form and the corresponding volume of solvent (the effect of dilution during the titration was also incorporated). Values of $[\mathbf{G}]_t$, $[\mathbf{W}]_t$ and $[\mathbf{H}]_t$ were interpolated and served for calculation of continuous values of all transition rate coefficients and concentrations shown in Fig. 7 by solid lines.

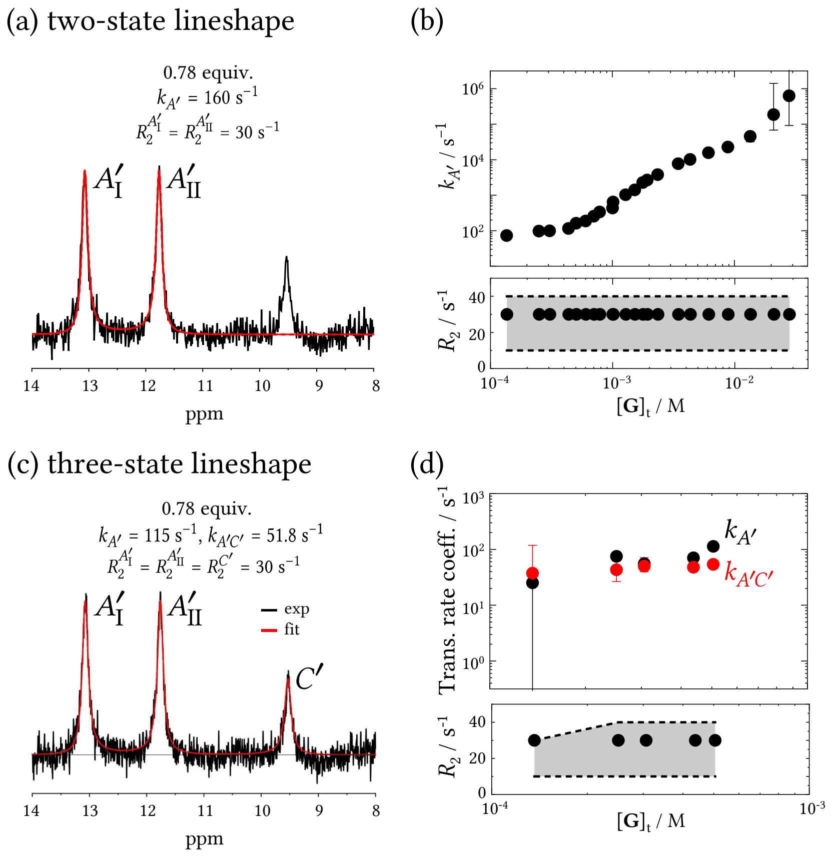


**Figure S24.** Fitting of two- and three-state exchange spectral lineshape and determination of transition rate coefficients in Step 1. (**a**) Example of two-state fitting (spectrum at 0.78 equiv. of guest). Parameters $\delta_{A'_I}$, $\delta_{A'_{II}}$ and $R_2^{A'_I} = R_2^{A'_{II}}$ were fixed during the fitting of one particular spectrum. Values of $k_{A'}$ and $M_0$ are fitted parameters (value of $M_0$ differs from the real total magnetization since it depends on technical details of the experimental setup and data processing). (**b**) Resulting values of $k_{A'}$ from two-state fitting (top) and the range of the $R_2$ parameter, where the fit was successful (grey zone, bottom). Full circles (top and bottom) correspond to $R_2 = 30\ \mathrm{s}^{-1}$, error bars denote maximum and minimum $k_{A'}$ values from repeated fitting procedure at different $R_2$ values in the range $R_2 \in [10\ \mathrm{s}^{-1}, 40\ \mathrm{s}^{-1}]$ (grey zone, bottom). (**c**) Example of three-state fitting. Parameters $\delta_{A'_I}$, $\delta_{A'_{II}}$, $\delta_{C'}$ and $R_2^{A'_I} = R_2^{A'_{II}} = R_2^{C'}$ were fixed during the fitting of one particular spectrum. Values of $k_{A'}$, $k_{A'C'}$ and $M_0$ are fitted parameters. (**d**) Resulting values of $k_{A'}$ and $k_{A'C'}$ from three-state fitting (top) and the range of the $R_2$ parameter (grey zone, bottom). Full circles (top and bottom) correspond to $R_2 = 30\ \mathrm{s}^{-1}$, error bars denote maximum and minimum $k_{A'C'}$, $k_{C'A'}$ values from the repeated fitting procedure at different $R_2$ values in the range $R_2 \in [10\ \mathrm{s}^{-1}, 40\ \mathrm{s}^{-1}]$ (grey zone, bottom).

Symmetric two-state exchange lineshape and half-symmetric three-state exchange lineshapes were fitted to the NH peaks

of the host to obtain the concentration dependence of transition rate coefficients. Parameters $\delta_{A'_{\mathrm{I}}}$, $\delta_{A'_{\mathrm{II}}}$ and $\delta_{C'}$ were obtained from the positions of peak maxima in the slow regime. In the intermediate and fast regime the Larmor frequencies shifted due to variation in solvent polarity, so the difference $|\delta_{A'_{\mathrm{I}}} - \delta_{A'_{\mathrm{II}}}|$ was fixed, while the center $(\delta_{A'_{\mathrm{I}}} + \delta_{A'_{\mathrm{II}}})/2$ was adjusted during the fitting, see grey dashed lines in Fig. 7a,b. Relaxation rates were assumed to be equal, i.e., $R_2^{A'_{\mathrm{I}}} = R_2^{A'_{\mathrm{II}}} = R_2^{C'}$. During the fitting procedure, the relaxation rates were fixed. The parameters adjusted during the fitting procedure are: $k_{A'}$ for two-state exchange, $k_{A'}$ and $k_{A'C'}$ for three-state exchange and total magnetization in relative units $M_0$. This fitting procedure was realized in *Mathematica* (using in-house scripts with `FindFit` command for the minimization procedure) as illustrated in Supplementary file `41598_2022_20136_MOESM5_ESM.nb` (note that this notebook has three Slides) or in its pdf version `41598_2022_20136_MOESM6_ESM.pdf`. The excellent match of fits is illustrated in Fig. S24a,c. The fitting procedure was repeated for every measured spectrum (at particular $[\mathbf{G}]_{\mathrm{t}}$) for different $R_2$ values, which were varied in a reasonable range of $R_2 \in [10\ \mathrm{s}^{-1}, 40\ \mathrm{s}^{-1}]$, grey zone in Fig. S24b,d (bottom). Upon visual inspection, the fitted lineshapes provided an excellent match to the experimental data in almost the whole range of the $R_2$ parameter (grey zone in Fig. S24b,d (bottom)). Maximum and minimum values of the fitted transition rate coefficients $k_{A'}$, $k_{A'C'}$ and $k_{C'A'}$ form the error bars in Fig. S24b,d (top). Mean $k_{A'}$, $k_{A'C'}$ and $k_{C'A'}$ values correspond to $R_2 = 30\ \mathrm{s}^{-1}$. Figure 7c in the main manuscript shows that the transition rate coefficients in the intermediate exchange regime are determined with the highest accuracy since the spectral lineshape at this point is most sensitive to the change of the transition rate coefficients (see also Sec. S7.4).

**Step 2 – Binding model fitting:**
Simultaneous fitting of $k_{A'}$ concentration dependence together (using Eq. (S54a)) with shift of fast-exchanging peaks of **H**/**HW** (denoted as $C'$ in Fig. 7b), two host TB resonances and two guest MET resonances (Fig. S25) was performed. This fit provides equilibrium binding constant $K_{\mathbf{HG}}$, reaction rate coefficient $\kappa_A$ and Larmor frequencies of the peaks involved. The following binding isotherms were fitted simultaneously

$$
\begin{aligned}
k_{A'} &= 2\kappa_A[\mathbf{G}], \\
\delta_{C'} &= \delta_C \frac{[\mathbf{H}]}{[\mathbf{H}]+[\mathbf{HW}]} + \delta_D \frac{[\mathbf{HW}]}{[\mathbf{H}]+[\mathbf{HW}]}, \\
\delta(\mathbf{G}\ \text{MET1 observed}) &= \delta(\mathbf{G}\ \text{MET1 free})\frac{[\mathbf{G}]}{[\mathbf{G}]_{\mathrm{t}}} + \delta(\mathbf{G}\ \text{MET1 bound})\frac{[\mathbf{HG}]}{[\mathbf{G}]_{\mathrm{t}}}, \\
\delta(\mathbf{G}\ \text{MET2 observed}) &= \delta(\mathbf{G}\ \text{MET2 free})\frac{[\mathbf{G}]}{[\mathbf{G}]_{\mathrm{t}}} + \delta(\mathbf{G}\ \text{MET2 bound})\frac{[\mathbf{HG}]}{[\mathbf{G}]_{\mathrm{t}}}, \\
\delta(\mathbf{H}\ \text{TB1 observed}) &= \delta(\mathbf{H}\ \text{TB1 free})\frac{[\mathbf{H}]}{[\mathbf{H}]_{\mathrm{t}}} + \delta(\mathbf{H}\ \text{TB1 bound})\frac{[\mathbf{HG}]}{[\mathbf{H}]_{\mathrm{t}}}, \\
\delta(\mathbf{H}\ \text{TB2 observed}) &= \delta(\mathbf{H}\ \text{TB2 free})\frac{[\mathbf{H}]}{[\mathbf{H}]_{\mathrm{t}}} + \delta(\mathbf{H}\ \text{TB2 bound})\frac{[\mathbf{HG}]}{[\mathbf{H}]_{\mathrm{t}}}.
\end{aligned}
$$

Concentrations of the chemical species were obtained from numerical solution of the competitive host–ligand binding model, summarized in Eqs. (S44) and (S45). The fitting procedure was realized in *Mathematica* (using in-house scripts) as illustrated in Supplementary file `41598_2022_20136_MOESM3_ESM.nb` (note that this notebook has two Slides) or in its pdf version `41598_2022_20136_MOESM4_ESM.pdf`. In order to obtain error of all fitted parameters, the fitting procedure was repeated several times with different fixed values of parameters $K_{\mathbf{HG}}$, $\kappa_A$, $\delta_C$ or $\delta_D$. Acceptable fits were determined by visual inspection of fitted curves and subsequently confidence intervals were constructed from minimum and maximum values of these fitted parameters. The parameter $\kappa_A$ was calculated as a mean, hence its error was determined as standard deviation. Furthermore, errors of other parameters, denoted here as $\sigma_\bullet$, were obtained from propagation of uncertainty. In particular, error of $\kappa_{CA}$ (where $\kappa_{CA} = K_{\mathbf{HG}}\kappa_{AC}/4$) was calculated as $\sigma_{\kappa_{AC}} = \sqrt{(\frac{\kappa_{AC}}{4})^2\sigma^2_{K_{\mathbf{HG}}} + (\frac{K_{\mathbf{HG}}}{4})^2\sigma^2_{\kappa_{AC}}}$, errors of Gibbs energy barriers $\Delta G_j^\ddagger$ (where $\Delta G_j^\ddagger = -RT\ln\frac{h\kappa_j}{k_{\mathrm{B}}T}$) were calculated as $\sigma_{\Delta G_j^\ddagger} = \sqrt{(\frac{RT}{\kappa_j})^2\sigma^2_{\kappa_j}}$ and errors of standard reaction Gibbs energies $\Delta G_j^\circ$ (where $\Delta G_j^\circ = -RT\ln K_j$) were calculated as $\sigma_{\Delta G_j^\circ} = \sqrt{(\frac{RT}{K_j})^2\sigma^2_{K_j}}$. All parameters fixed or fitted during the fitting procedures in Step 1 and Step 2 are listed in Table S6.

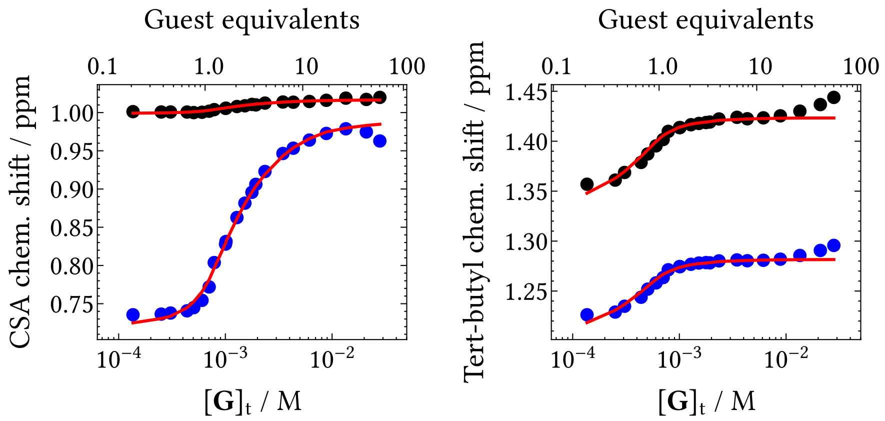


**Figure S25.** Result of simultaneous fitting of chemical shifts of guest's MET resonances (left panel) and host's TB resonances (right panel) during the Step 2.

**Table S6.** Overview of fitted and fixed parameters in the fitting procedures in Step 1 and Step 2.

| Parameter | Value | Comment |
|---|---|---|
| $k_{A'C'}$ | $47 \pm 6$ s$^{-1}$ | mean from raw data fit (Step 1) |
| $k_{A'}$ | $10 - 10^5$ s$^{-1}$ | fitted (Step 1), changing with concentration |
| $K_{\mathbf{HG}}$ | $(7.4 \pm 3.0) \times 10^4$ M$^{-1}$ | fitted (Step 2), |
| | | reported value[S31] $(8.0 \pm 5.0) \times 10^4$ M$^{-1}$ |
| $K_{\mathbf{HW}}$ | $240 \pm 35$ M$^{-1}$ | reported value,[S25] fixed during Step 2 |
| $\kappa_A$ | $(10 \pm 1) \times 10^5$ M$^{-1}$s$^{-1}$ | fitted (Step 2), binding isotherm $k_{A'} = 2\kappa_A[\mathbf{G}]$ |
| $\kappa_{AC}$ | $47 \pm 6$ s$^{-1}$ | $\kappa_{AC} = k_{A'C'}$, mean from raw data fit (Step 1), |
| | | fixed during Step 2 |
| $\kappa_{CA}$ | $(9 \pm 4) \times 10^5$ M$^{-1}$s$^{-1}$ | calculated, $\kappa_{CA} = K_{\mathbf{HG}}\kappa_{AC}/4$, |
| $\delta_{A'_{\mathrm{I}}}$ | 13.08 ppm | at low $[\mathbf{G}]_t$; fixed (Step 1), obtained from peak maxima, |
| | <13.08 ppm | at high $[\mathbf{G}]_t$; fitted with fixed $\delta_{A'_{\mathrm{I}}} - \delta_{A'_{\mathrm{II}}}$ (Step 1) |
| $\delta_{A'_{\mathrm{II}}}$ | 11.76 ppm | at low $[\mathbf{G}]_t$; fixed (Step 1), obtained from peak maxima, |
| | <11.76 ppm | at high $[\mathbf{G}]_t$; fitted with fixed $\delta_{A'_{\mathrm{I}}} - \delta_{A'_{\mathrm{II}}}$ (Step 1) |
| $\delta_{C'}$ | $9.19 - 9.65$ ppm | fixed, changing with concentration, |
| | | obtained from the peak maxima (Step 1) |
| $\delta_C$ | $7.8 \pm 0.2$ ppm | fitted (Step 2), reported value[S25] $7.86 \pm 0.40$ ppm |
| $\delta_D$ | $10.4 \pm 0.2$ ppm | fitted (Step 2), reported value[S25] $10.09 \pm 0.03$ ppm |
| $\delta$(**G** MET1 free) | $1.02 \pm 0.01$ ppm | fitted (Step 2) |
| $\delta$(**G** MET1 bound) | $1.00 \pm 0.01$ ppm | fitted (Step 2) |
| $\delta$(**G** MET2 free) | $0.99 \pm 0.01$ ppm | fitted (Step 2) |
| $\delta$(**G** MET2 bound) | $0.71 \pm 0.01$ ppm | fitted (Step 2) |
| $\delta$(**H** TB1 free) | $1.33 \pm 0.01$ ppm | fitted (Step 2) |
| $\delta$(**H** TB1 bound) | $1.42 \pm 0.01$ ppm | fitted (Step 2) |
| $\delta$(**H** TB2 free) | $1.20 \pm 0.01$ ppm | fitted (Step 2) |
| $\delta$(**H** TB2 bound) | $1.28 \pm 0.01$ ppm | fitted (Step 2) |

## References


[S1] Kartha, K. K., Takai, A., Futera, Z., Labuta, J. & Takeuchi, M. Dynamics of Meso–Chiral Interconversion in a Butterfly-Shape Overcrowded Alkene Rotor Tunable by Solvent Properties. *Angewandte Chemie Int. Ed.* **60**, 16466–16471, DOI: https://doi.org/10.1002/anie.202102719 (2021).

[S2] Feng, C., Kovrigin, E. L. & Post, C. B. NmrLineGuru: Standalone and User-Friendly GUIs for Fast 1D NMR Lineshape Simulation and Analysis of Multi-State Equilibrium Binding Models. *Sci. Rep.* **9**, 1–14, DOI: https://doi.org/10.1038/s41598-019-52451-8 (2019).

[S3] Levitt, M. H. *Spin Dynamics: Basics of Nuclear Magnetic Resonance* (John Wiley & Sons, 2001).

[S4] Bain, A. D. Chemical exchange in NMR. *Prog. Nucl. Magn. Reson. Spectrosc.* **43**, 63–103, DOI: https://doi.org/10.1016/j.pnmrs.2003.08.001 (2003).

[S5] Římal, V., Štěpánková, H. & Štěpánek, J. Analysis of NMR spectra in case of temperature-dependent chemical exchange between two unequally populated sites. *Concepts Magn. Reson. Part A* **38A**, 117–127, DOI: https://doi.org/10.1002/cmr.a.20214 (2011).

[S6] Cavanagh, J., Fairbrother, W. J., Palmer, A. G. & Skelton, N. J. *Protein NMR Spectroscopy: Principles and Practice* (Academic Press, 1996).

[S7] Kovrigin, E. L. NMR line shapes and multi-state binding equilibria. *J. Biomol. NMR* **53**, 257–270, DOI: https://doi.org/10.1007/s10858-012-9636-3 (2012).

[S8] Greenwood, A. I. *et al.* Complete determination of the Pin1 catalytic domain thermodynamic cycle by NMR lineshape analysis. *J. Biomol. NMR* **51**, 21–34, DOI: https://doi.org/10.1007/s10858-011-9538-9 (2011).

[S9] Binsch, G. *Topics in Stereochemistry*, vol. 3 (Interscience publishers., 1968).

[S10] Günther, U. L. & Schaffhausen, B. NMRKIN: Simulating line shapes from two-dimensional spectra of proteins upon ligand binding. *J. Biomol. NMR* **22**, 201–209, DOI: https://doi.org/10.1023/A:1014985726029 (2002).

[S11] Post, C. B. [19] Characterization of enzyme-complex formation by analysis of nuclear magnetic resonance line shapes. In *Methods in Enzymology*, vol. 240 of *Part B: Numerical Computer Methods*, 438–446, DOI: https://doi.org/10.1016/S0076-6879(94)40058-X (Academic Press, 1994).

[S12] Anamimoghadam, O. *et al.* Discrete Open-Shell Tris(bipyridinium radical cationic) Inclusion Complexes in the Solid State. *J. Am. Chem. Soc.* **143**, 163–175, DOI: https://doi.org/10.1021/jacs.0c07148 (2021).

[S13] Biali, S. E., Nugiel, D. A. & Rappoport, Z. Stable simple enols. Part 19. Steric effects and threshold rotational mechanisms in 1-substituted 2,2-dimesitylethenols. *J. Am. Chem. Soc.* **111**, 846–852, DOI: https://doi.org/10.1021/ja00185a010 (1989).

[S14] Baker, G. A., Jr. & Graves-Morris, P. *Padé Approximants: Encyclopedia of Mathematics and It's Applications*, vol. 59 (Cambridge University Press, 1996), second edn.

[S15] Labuta, J. *et al.* Chiral Guest Binding as a Probe of Macrocycle Dynamics and Tautomerism in a Conjugated Tetrapyrrole. *J. Am. Chem. Soc.* **136**, 2112–2118, DOI: https://doi.org/10.1021/ja4124175 (2014).

[S16] Mulquiney, P. J. & Kuchel, P. W. *Modelling Metabolism with Mathematica: Detailed Examples Including Erythrocyte Metabolism* (CRC Press, Boca Raton, Fla, 2003).

[S17] Furukawa, A., Konuma, T., Yanaka, S. & Sugase, K. Quantitative analysis of protein–ligand interactions by NMR. *Prog. Nucl. Magn. Reson. Spectrosc.* **96**, 47–57, DOI: https://doi.org/10.1016/j.pnmrs.2016.02.002 (2016).

[S18] Nitschke, P., Lokesh, N. & Gschwind, R. M. Combination of illumination and high resolution NMR spectroscopy: Key features and practical aspects, photochemical applications, and new concepts. *Prog. Nucl. Magn. Reson. Spectrosc.* **114–115**, 86–134, DOI: https://doi.org/10.1016/j.pnmrs.2019.06.001 (2019).

[S19] Grilli, S., Lunazzi, L., Mazzanti, A., Casarini, D. & Femoni, C. Conformational Studies by Dynamic NMR. 78.1 Stereomutation of the Helical Enantiomers of Trigonal Carbon Diaryl-Substituted Compounds: Dimesitylketone, Dimesitylthioketone, and Dimesitylethylene. *J. Org. Chem.* **66**, 488–495, DOI: https://doi.org/10.1021/jo0012871 (2001).

[S20] Grilli, S., Lunazzi, L., Mazzanti, A. & Mazzanti, G. Conformational Studies by Dynamic NMR. 79.1 Dimesityl Sulfine Revisited: Detection of the Helical Antipodes and Determination of Their Enantiomerization Pathways. *J. Org. Chem.* **66**, 748–754, DOI: https://doi.org/10.1021/jo001408h (2001).

[S21] Biali, S. E. & Rappoport, Z. Stable simple enols. 3. Static and dynamic NMR behavior of crowded triarylethenols and related compounds. Three-ring flip as the threshold mechanism for enantiomerization of crowded triarylvinyl propellers. *J. Am. Chem. Soc.* **106**, 477–496, DOI: https://doi.org/10.1021/ja00315a003 (1984).

[S22] Gust, D. & Mislow, K. Analysis of isomerization in compounds displaying restricted rotation of aryl groups. *J. Am. Chem. Soc.* **95**, 1535–1547, DOI: https://doi.org/10.1021/ja00786a031 (1973).

[S23] Kleckner, I. R. & Foster, M. P. An introduction to NMR-based approaches for measuring protein dynamics. *Biochimica et Biophys. Acta (BBA) - Proteins Proteomics* **1814**, 942–968, DOI: https://doi.org/10.1016/j.bbapap.2010.10.012 (2011).

[S24] Bryant, R. G. The NMR time scale. *J. Chem. Educ.* **60**, 933, DOI: https://doi.org/10.1021/ed060p933 (1983).

[S25] Labuta, J. *et al.* NMR spectroscopic detection of chirality and enantiopurity in referenced systems without formation of diastereomers. *Nat. Commun.* **4**, 2188, DOI: https://doi.org/10.1038/ncomms3188 (2013).

[S26] Hargrove, A. E., Zhong, Z., Sessler, J. L. & Anslyn, E. V. Algorithms for the determination of binding constants and enantiomeric excess in complex host : Guest equilibria using optical measurements. *New J. Chem.* **34**, 348–354, DOI: https://doi.org/10.1039/B9NJ00498J (2010).

[S27] Connors, K. A. *Binding Constants: The Measurement of Molecular Complex Stability* (Wiley, 1987).

[S28] Muddana, H. S. & Gilson, M. K. Calculation of Host–Guest Binding Affinities Using a Quantum-Mechanical Energy Model. *J. Chem. Theory Comput.* **8**, 2023–2033, DOI: https://doi.org/10.1021/ct3002738 (2012).

[S29] Atkins, P. W., Paula, J. D. & Keeler, J. *Atkins' Physical Chemistry* (Oxford University Press, 2018).

[S30] Ercolani, G. Assessment of Cooperativity in Self-Assembly. *J. Am. Chem. Soc.* **125**, 16097–16103, DOI: https://doi.org/10.1021/ja038396c (2003).

[S31] Březina, V. *et al.* Structural Modulation of Chromic Response: Effects of Binding-Site Blocking in a Conjugated Calix[4]pyrrole Chromophore. *ChemistryOpen* **7**, 323–335, DOI: https://doi.org/10.1002/open.201800005 (2018).
