# Supplementary material for: NMR lineshape analysis using analytical solutions of multi-state chemical exchange with applications to kinetics of host–guest systems
Source: Sci Rep. 2022 Oct 17;12:17369. doi: 10.1038/s41598-022-20136-4 (PMC9576801; doi:10.1038/s41598-022-20136-4)
Supplement: Supplementary file 4 — Supplementary Information 4. [file 41598_2022_20136_MOESM4_ESM.pdf]

Slide 1

Experimental data

In ‘Evaluation’ select ‘Evaluate Initialization Cells’ to load the experimental data and to initialize the fitting functions.

No. of guest equivalents was obtained from peak integration and [H]t from measurements of sample mass and solvent volume. These values provide [G]t = guest equiv. * [H]t.
No. of water equivalents was also obtained from peak integration and yields [W]t = water equiv. * [H]t (the data points were intepolated by a smooth curve).

Legend:
`eqData` ... No. of guest equivalents
`HtData` ... [H]t
`GtData` ... [G]t
`WtData` ... [W]t
`log10kAData` ... logarithm of kA’ values
`δCprimeData` ... values of δC’
`MET1Data` ... δ(G MET1 observed)
`MET2Data` ... δ(G MET2 observed)
`TB1Data` ... δ(G TB1 observed)
`TB2Data` ... δ(G TB2 observed)

```
eqData={0.207311365`,0.382375373`,0.469249973`,0.67242578`,0.779079114`,0.934428144`,
1.094196882`,1.226635839`,1.573420242`,1.59933349`,2.040652857`,2.442411868`,2.864300
866`,3.142037489`,3.885345735`,5.988144379`,7.819192705`,11.83401689`,18.95947097`,28
.89347006`};

HtData={0.000657181`,0.000655009`,0.000652851`,0.000650707`,0.000648577`,0.000646461`
,0.000644358`,0.00064123`,0.000638133`,0.000634049`,0.000629017`,0.000624064`,0.00061
9188`,0.000614388`,0.000605008`,0.000578512`,0.000554238`,0.000518014`,0.000466212`,0
.000466212`};

GtData=eqData*HtData;

WtData={0.006539454012927474`,0.007332924195850111`,0.007698594234269399 5`,0.00849863
2891959746`,0.008879661237430888`,0.009396168063013808`,0.009409797339253 74`,0.010555
6012134631`,0.011069682219781711`,0.01155742804633651`,0.01229245352487474 1`,0.013536
70395766976`,0.01486626456611 4`,0.0149236260749952`,0.013050972356009119`,0.015364572
98742608`,0.015037222611805 2`,0.01652438216939342`,0.018187671681469317`,0.0225156554
3379108`};

log10kAData={1.4013597661268054`,1.8754583985682218`,1.744767827525096`,1.84923351925
7295`,2.0547351237367737`,2.2751335404347137`,2.4092191306874966`,2.528858205871014 5`
,2.640384547848104`,2.808781913815932`,3.014378349681189`,3.149808052077982`,3.357423
3210606206`,3.429156095343393`,3.580946331457232`,3.887849609328694`,4.00753093573043
4`,4.196600928681497`,4.358087382619229`,4.657839416177932`};
```

```
δCprimeData={9.339029`,9.40295987`,9.433287193`,9.482102423`,9.529621846`,9.585042626
`};

MET1Data={1.00121764`,1.000756574`,1.000761747`,1.000681525`,0.999980526`,1.000411762
`,1.00179248`,1.003842881`,1.005492556`,1.005727926`,1.0078847`,1.008847915`,1.010639
365`,1.010026634`,1.012383747`,1.013797182`,1.013530664`,1.014529311`,1.016059664`,1.
018872363`};

MET2Data={0.73553824`,0.736367938`,0.737795474`,0.740700598`,0.744873649`,0.75438175`
,0.77191815`,0.803781209`,0.827971709`,0.831388648`,0.862676189`,0.881532001`,0.89568
0913`,0.906112297`,0.922923636`,0.946466956`,0.953369627`,0.964040753`,0.97267027`,0.
978793005`};

TB1Data={1.356935797`,1.361095284`,1.368718657`,1.378772363`,1.387267194`,1.395338688
`,1.401703497`,1.409873969`,1.413654671`,1.413717084`,1.416509045`,1.417803306`,1.418
693873`,1.419201284`,1.422299351`,1.423937472`,1.422500011`,1.423417998`,1.425419478`
,1.430104277`};

TB2Data={1.226249969`,1.228957348`,1.234788209`,1.243792865`,1.251896765`,1.258154295
`,1.263515968`,1.271282476`,1.274545193`,1.274415151`,1.276783467`,1.278058858`,1.278
48487`,1.27824053`,1.280205266`,1.281157708`,1.28035384`,1.280795042`,1.281908328`,1.
285613178`};
```

Slide 2

Fitting procedure

kA values at RA = RC = 30
MET - methyl protons of guest
TB - tert-butyl protons of host
Concentrations [H], [HG], [HW], [G] and [W] were calculated numerically according to Eq. (S43) and (S44) in Supplementary Information.

functions for calculation of concentrations

```
H[KHG_,KHW_,Ht_,G_,W_]:=Ht/(1+G KHG+KHW W);
HG[KHG_,KHW_,Ht_,G_,W_]:=G Ht KHG/(1+G KHG+KHW W);
HW[KHG_,KHW_,Ht_,G_,W_]:=Ht KHW W/(1+G KHG+KHW W);
G[KHG_?NumericQ,KHW_?NumericQ,Ht_,Gt_,Wt_]:=G/. FindRoot[{Gt-G^2 KHG+Gt KHW W+G (-
1+Gt KHG-Ht KHG-KHW W)==0,-KHW W^2+Wt+G KHG Wt+W (-1-G KHG-Ht KHW+KHW
Wt)==0},{{G,Gt},{W,Wt}}];
W[KHG_?NumericQ,KHW_?NumericQ,Ht_,Gt_,Wt_]:=W/. FindRoot[{Gt-G^2 KHG+Gt KHW W+G (-
1+Gt KHG-Ht KHG-KHW W)==0,-KHW W^2+Wt+G KHG Wt+W (-1-G KHG-Ht KHW+KHW
Wt)==0},{{G,Gt},{W,Wt}}];
SetAttributes[G,Listable];
SetAttributes[W,Listable];
```

the fitting procedure

Fitting of binding isotherms as described in Sec. S12

```
fitRule=FindMinimum[{Gval=G[KHG,(*KHW*)240,HtData,GtData,WtData];Wval=W[KHG,(*KHW*)24
0,HtData,GtData,WtData];

Hval=H[KHG,(*KHW*)240,HtData,Gval,Wval];HGval=HG[KHG,(*KHW*)240,HtData,Gval,Wval];HWv
al=HW[KHG,(*KHW*)240,HtData,Gval,Wval];
    Total[(log10kAData-Log10[2κA Gval])^2]
     +Total[(δCprimeData-
(δC*Hval/(Hval+HWval)+δD*HWval/(Hval+HWval))[[1;;Length@δCprimeData]] )^2]
     +Total[(MET1Data-(δMET1free*Gval/GtData+δMET1bound*HGval/GtData) )^2]
     +Total[(MET2Data-(δMET2free*Gval/GtData+δMET2bound*HGval/GtData) )^2]
     +Total[(TB1Data-(δTB1free*(Hval+HWval)/HtData+δTB1bound*HGval/HtData) )^2]
     +Total[(TB2Data-(δTB2free*(Hval+HWval)/HtData+δTB2bound*HGval/HtData) )^2]},
{{κA,1000000.},{δMET1free,1.02},{δMET1bound,1.},{δMET2free,1.0},{δMET2bound,.75},{δTB
1free,1.34},{δTB1bound,1.425},{δTB2free,1.21},{δTB2bound,1.28},{δC,7.7(* NH free
7.86+-.4 *)},{δD,10.4(* NH water 10.09 *)},{KHG,80000}}][[2]]

{κA->969395.,δMET1free->1.0167,δMET1bound->0.998027,δMET2free->0.989356,δMET2bound-
>0.70765,δTB1free->1.32962,δTB1bound->1.42388,δTB2free->1.20303,δTB2bound-
>1.28198,δC->7.77239,δD->10.3699,KHG->73044.1}
```

plot of fitting results

```
Grid[{{
   Show[{ListPlot[Transpose@{Log10@GtData,log10kAData},ImageSize->400,AspectRatio->1.618/2,Axes->False,Frame->True,BaseStyle->{FontSize->24},FrameLabel->{Style["Log10 [G]t"],Style["Log10 kA"]},PlotStyle->{Black,PointSize[.03]},ImagePadding->{{100,15},{70,15}}],ListLinePlot[Transpose@{Log10@GtData,Log10[2κA Gval]/.fitRule},PlotStyle->Red]}],
   Show[{ListPlot[{Transpose@{Log10@GtData,MET1Data},Transpose@{Log10@GtData,MET2Data}},ImageSize->400,AspectRatio->1.618/2,Axes->False,Frame->True,BaseStyle->{FontSize->24},FrameLabel->{Style["Log10 [G]t"],Style["MET chem. shift"]},PlotStyle->Directive[Black,PointSize[.03]],ImagePadding->{{100,15},{70,15}}],
ListLinePlot[{Transpose@{Log10@GtData,δMET1free*Gval/GtData+δMET1bound*HGval/GtData/.fitRule},Transpose@{Log10@GtData,δMET2free*Gval/GtData+δMET2bound*HGval/GtData/.fitRule}},PlotStyle->Red]}],
   Show[{ListPlot[{Transpose@{Log10@GtData,TB1Data},Transpose@{Log10@GtData,TB2Data}},ImageSize->400,AspectRatio->1.618/2,Axes->False,Frame->True,BaseStyle->{FontSize->24},FrameLabel->{Style["Log10 [G]t"],Style["TB chem. shift"]},PlotStyle->Directive[Black,PointSize[.03]],ImagePadding->{{100,15},{70,15}}],
ListLinePlot[{Transpose@{Log10@GtData,δTB1free*(Hval+HWval)/HtData+δTB1bound*HGval/HtData/.fitRule},Transpose@{Log10@GtData,δTB2free*(Hval+HWval)/HtData+δTB2bound*HGval/HtData/.fitRule}},PlotStyle->Red]}],
   Show[{ListPlot[Transpose@{Log10@GtData[[1;;Length@δCprimeData]],δCprimeData},ImageSize->400,AspectRatio->1.618/2,Axes->False,Frame->True,BaseStyle->{FontSize->24},FrameLabel->{Style["Log10 [G]t"],Style["δC'"]},PlotStyle->Directive[Black,PointSize[.03]],ImagePadding->{{100,15},{70,15}}],
ListLinePlot[Transpose@{Log10@GtData[[1;;Length@δCprimeData]],(δC*Hval/(Hval+HWval)+δD*HWval/(Hval+HWval))[[1;;Length@δCprimeData]]/.fitRule},PlotStyle->Red]  }]  }}]
```

{

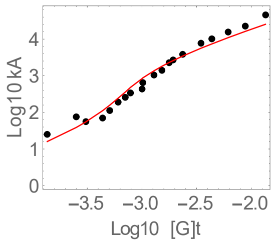

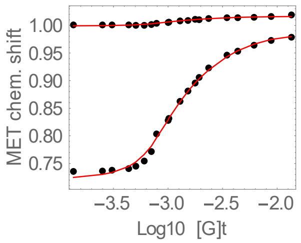

{ , ,

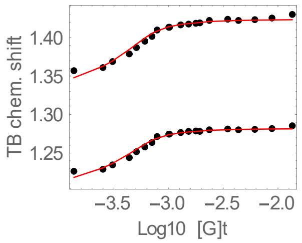

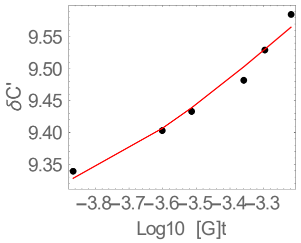

, }}
