# Supplementary material for: NMR lineshape analysis using analytical solutions of multi-state chemical exchange with applications to kinetics of host–guest systems
Source: Sci Rep. 2022 Oct 17;12:17369. doi: 10.1038/s41598-022-20136-4 (PMC9576801; doi:10.1038/s41598-022-20136-4)
Supplement: Supplementary file 6 — Supplementary Information 6. [file 41598_2022_20136_MOESM6_ESM.pdf]

Slide 1

Experimental data

In ‘Evaluation’ select ‘Evaluate Initialization Cells’ to load the experimental data and to initialize the fitting functions.

```
ppmData={8.00061406598522`,8.009714065985218`,8.01891406598522`,8.028014065985218`,8.
03721406598522`,8.04631406598522`,8.05551406598522`,8.06461406598522`,8.0738140659852
2`,8.08291406598522`,8.09211406598522`,8.10121406598522`,8.11041406598522`,8.11951406
598522`,8.12871406598522`,8.13791406598522`,8.14701406598522`,8.15621406598522`,8.165
31406598522`,8.17451406598522`,8.18361406598522`,8.19281406598522`,8.20191406598522`,
8.21111406598522`,8.22021406598522`,8.22941406598522`,8.23851406598522`,8.24771406598
522`,8.25681406598522`,8.26601406598522`,8.27511406598522`,8.28431406598522`,8.293414
06598522`,8.30261406598522`,8.311814065985219`,8.32091406598522`,8.330114065985219`,8
.33921406598522`,8.348414065985219`,8.35751406598522`,8.366714065985219`,8.3758140659
8522`,8.385014065985219`,8.39411406598522`,8.403314065985219`,8.41241406598522`,8.421
614065985219`,8.430714065985219`,8.439914065985219`,8.449014065985219`,8.458214065985
219`,8.467414065985219`,8.476514065985219`,8.485714065985219`,8.494814065985219`,8.50
4014065985219`,8.513114065985219`,8.522314065985219`,8.531414065985219`,8.54061406598
5219`,8.549714065985219`,8.558914065985219`,8.568014065985219`,8.577214065985219`,8.5
86314065985219`,8.595514065985219`,8.604614065985219`,8.613814065985219`,8.6230140659
85219`,8.632114065985219`,8.641314065985219`,8.650414065985219`,8.659614065985219`,8.
668714065985219`,8.677914065985219`,8.687014065985219`,8.696214065985219`,8.705314065
985219`,8.714514065985218`,8.723614065985219`,8.732814065985218`,8.741914065985219`,8
.751114065985218`,8.760214065985219`,8.769414065985218`,8.77861406598522`,8.787714065
985218`,8.79691406598522`,8.806014065985218`,8.81521406598522`,8.824314065985218`,8.8
3351406598522`,8.842614065985218`,8.85181406598522`,8.860914065985218`,8.870114065985
22`,8.87921406598522`,8.88841406598522`,8.89751406598522`,8.90671406598522`,8.9158140
6598522`,8.92501406598522`,8.93421406598522`,8.94331406598522`,8.95251406598522`,8.96
161406598522`,8.97081406598522`,8.97991406598522`,8.98911406598522`,8.99821406598522`
,9.00741406598522`,9.01651406598522`,9.02571406598522`,9.03481406598522`,9.0440140659
8522`,9.05311406598522`,9.06231406598522`,9.07141406598522`,9.08061406598522`,9.08981
406598522`,9.09891406598522`,9.10811406598522`,9.11721406598522`,9.12641406598522`,9.
13551406598522`,9.14471406598522`,9.15381406598522`,9.16301406598522`,9.1721140659852
2`,9.18131406598522`,9.19041406598522`,9.19961406598522`,9.20871406598522`,9.21791406
598522`,9.22701406598522`,9.23621406598522`,9.24541406598522`,9.25451406598522`,9.263
71406598522`,9.27281406598522`,9.28201406598522`,9.29111406598522`,9.30031406598522`,
9.30941406598522`,9.31861406598522`,9.32771406598522`,9.33691406598522`,9.34601406598
522`,9.35521406598522`,9.36431406598522`,9.37351406598522`,9.38261406598522`,9.391814
06598522`,9.401014065985219`,9.41011406598522`,9.419314065985219`,9.42841406598522`,9
.437614065985219`,9.44671406598522`,9.455914065985219`,9.465014065985219`,9.474214065
985219`,9.483314065985219`,9.492514065985219`,9.501614065985219`,9.510814065985219`,9
.519914065985219`,9.529114065985219`,9.538214065985219`,9.547414065985219`,9.55661406
5985219`,9.565714065985219`,9.574914065985219`,9.584014065985219`,9.593214065985219`,
9.602314065985219`,9.611514065985219`,9.620614065985219`,9.629814065985219`,9.6389140
65985219`,9.648114065985219`,9.657214065985219`,9.666414065985219`,9.675514065985219`
,9.684714065985219`,9.693814065985219`,9.703014065985219`,9.712214065985219`,9.721314
065985219`,9.730514065985219`,9.739614065985219`,9.748814065985218`,9.757914065985219
`,9.767114065985218`,9.776214065985219`,9.785414065985218`,9.794514065985219`,9.80371
4065985218`,9.812814065985219`,9.822014065985218`,9.831114065985219`,9.84031406598521
8`,9.849414065985219`,9.858614065985218`,9.86781406598522`,9.876914065985218`,9.88611
406598522`,9.89521406598522`,9.90441406598522`,9.91351406598522`,9.92271406598522`,9.
```

93181406598522`,9.94101406598522`,9.95011406598522`,9.95931406598522`,9.9684140659852
2`,9.97761406598522`,9.98671406598522`,9.99591406598522`,10.00501406598522`,10.014214
06598522`,10.02341406598522`,10.03251406598522`,10.04171406598522`,10.05081406598522`
,10.06001406598522`,10.06911406598522`,10.07831406598522`,10.08741406598522`,10.09661
406598522`,10.10571406598522`,10.11491406598522`,10.12401406598522`,10.13321406598522
`,10.14231406598522`,10.15151406598522`,10.16061406598522`,10.16981406598522`,10.1790
1406598522`,10.18811406598522`,10.19731406598522`,10.20641406598522`,10.2156140659852
2`,10.22471406598522`,10.23391406598522`,10.24301406598522`,10.25221406598522`,10.261
31406598522`,10.27051406598522`,10.27961406598522`,10.28881406598522`,10.297914065985
22`,10.30711406598522`,10.31621406598522`,10.32541406598522`,10.33451406598522`,10.34
371406598522`,10.35291406598522`,10.36201406598522`,10.37121406598522`,10.38031406598
522`,10.38951406598522`,10.39861406598522`,10.40781406598522`,10.41691406598522`,10.4
261406598522`,10.43521406598522`,10.44441406598522`,10.45351406598522`,10.4627140659
8522`,10.47181406598522`,10.48101406598522`,10.49011406598522`,10.499314065985219`,10
.508514065985219`,10.517614065985219`,10.526814065985219`,10.535914065985219`,10.5451
14065985219`,10.554214065985219`,10.563414065985219`,10.572514065985219`,10.581714065
985219`,10.590814065985219`,10.600014065985219`,10.609114065985219`,10.61831406598521
9`,10.627414065985219`,10.636614065985219`,10.645714065985219`,10.654914065985219`,10
.664114065985219`,10.673214065985219`,10.682414065985219`,10.691514065985219`,10.7007
14065985219`,10.709814065985219`,10.719014065985219`,10.728114065985219`,10.737314065
985219`,10.746414065985219`,10.755614065985219`,10.764714065985219`,10.77391406598521
9`,10.783014065985219`,10.792214065985219`,10.801314065985219`,10.810514065985219`,10
.819714065985218`,10.828814065985219`,10.838014065985218`,10.847114065985219`,10.8563
14065985218`,10.865414065985219`,10.874614065985218`,10.883714065985219`,10.892914065
985218`,10.902014065985218`,10.911214065985218`,10.920314065985218`,10.92951406598522
`,10.938614065985218`,10.94781406598522`,10.956914065985218`,10.96611406598522`,10.97
531406598522`,10.98441406598522`,10.99361406598522`,11.00271406598522`,11.01191406598
522`,11.02101406598522`,11.03021406598522`,11.03931406598522`,11.04851406598522`,11.0
5761406598522`,11.06681406598522`,11.07591406598522`,11.08511406598522`,11.0942140659
8522`,11.10341406598522`,11.11251406598522`,11.12171406598522`,11.13091406598522`,11.
14001406598522`,11.14921406598522`,11.15831406598522`,11.16751406598522`,11.176614065
98522`,11.18581406598522`,11.19491406598522`,11.20411406598522`,11.21321406598522`,11
.22241406598522`,11.23151406598522`,11.24071406598522`,11.24981406598522`,11.25901406
598522`,11.26811406598522`,11.27731406598522`,11.28651406598522`,11.29561406598522`,1
1.30481406598522`,11.31391406598522`,11.32311406598522`,11.33221406598522`,11.3414140
6598522`,11.35051406598522`,11.35971406598522`,11.36881406598522`,11.37801406598522`,
11.38711406598522`,11.39631406598522`,11.40541406598522`,11.41461406598522`,11.423714
06598522`,11.43291406598522`,11.44211406598522`,11.45121406598522`,11.46041406598522`
,11.46951406598522`,11.47871406598522`,11.48781406598522`,11.49701406598522`,11.50611
406598522`,11.51531406598522`,11.52441406598522`,11.533614065985219`,11.5427140659852
2`,11.551914065985219`,11.56101406598522`,11.570214065985219`,11.57931406598522`,11.5
88514065985219`,11.597714065985219`,11.606814065985219`,11.616014065985219`,11.625114
065985219`,11.634314065985219`,11.643414065985219`,11.652614065985219`,11.66171406598
5219`,11.670914065985219`,11.680014065985219`,11.689214065985219`,11.698314065985219`
,11.707514065985219`,11.716614065985219`,11.725814065985219`,11.734914065985219`,11.7
44114065985219`,11.753314065985219`,11.762414065985219`,11.771614065985219`,11.780714
065985219`,11.789914065985219`,11.799014065985219`,11.808214065985219`,11.81731406598
5219`,11.826514065985219`,11.835614065985219`,11.844814065985219`,11.853914065985219`
,11.863114065985219`,11.872214065985219`,11.881414065985219`,11.890514065985219`,11.8
99714065985219`,11.908914065985218`,11.918014065985219`,11.927214065985218`,11.936314
065985218`,11.945514065985218`,11.954614065985218`,11.96381406598522`,11.972914065985
218`,11.98211406598522`,11.991214065985218`,12.00041406598522`,12.009514065985218`,12
.01871406598522`,12.027814065985218`,12.03701406598522`,12.046114065985218`,12.055314
06598522`,12.06451406598522`,12.07361406598522`,12.08281406598522`,12.09191406598522`
,12.10111406598522`,12.11021406598522`,12.11941406598522`,12.12851406598522`,12.13771

```
406598522`,12.14681406598522`,12.15601406598522`,12.16511406598522`,12.17431406598522
`,12.18341406598522`,12.19261406598522`,12.20171406598522`,12.21091406598522`,12.2201
1406598522`,12.22921406598522`,12.23841406598522`,12.24751406598522`,12.2567140659852
2`,12.26581406598522`,12.27501406598522`,12.28411406598522`,12.29331406598522`,12.302
41406598522`,12.31161406598522`,12.32071406598522`,12.32991406598522`,12.339014065985
22`,12.34821406598522`,12.35731406598522`,12.36651406598522`,12.37571406598522`,12.38
481406598522`,12.39401406598522`,12.40311406598522`,12.41231406598522`,12.42141406598
522`,12.43061406598522`,12.43971406598522`,12.44891406598522`,12.45801406598522`,12.4
6721406598522`,12.47631406598522`,12.48551406598522`,12.49461406598522`,12.5038140659
8522`,12.51291406598522`,12.52211406598522`,12.53121406598522`,12.54041406598522`,12.
54961406598522`,12.55871406598522`,12.567914065985219`,12.57701406598522`,12.58621406
5985219`,12.59531406598522`,12.604514065985219`,12.61361406598522`,12.622814065985219
`,12.63191406598522`,12.641114065985219`,12.65021406598522`,12.659414065985219`,12.66
851406598522`,12.677714065985219`,12.686814065985219`,12.696014065985219`,12.70521406
5985219`,12.714314065985219`,12.723514065985219`,12.732614065985219`,12.7418140659852
19`,12.750914065985219`,12.760114065985219`,12.769214065985219`,12.778414065985219`,1
2.787514065985219`,12.796714065985219`,12.805814065985219`,12.815014065985219`,12.824
114065985219`,12.833314065985219`,12.842414065985219`,12.851614065985219`,12.86081406
5985219`,12.869914065985219`,12.879114065985219`,12.888214065985219`,12.8974140659852
19`,12.906514065985219`,12.915714065985219`,12.924814065985219`,12.934014065985219`,1
2.943114065985219`,12.952314065985219`,12.961414065985219`,12.970614065985218`,12.979
714065985219`,12.988914065985218`,12.998014065985219`,13.007214065985218`,13.01641406
598522`,13.025514065985218`,13.03471406598522`,13.043814065985218`,13.05301406598522`
,13.062114065985218`,13.07131406598522`,13.080414065985218`,13.08961406598522`,13.098
714065985218`,13.10791406598522`,13.11701406598522`,13.12621406598522`,13.13531406598
522`,13.14451406598522`,13.15361406598522`,13.16281406598522`,13.17201406598522`,13.1
8111406598522`,13.19031406598522`,13.19941406598522`,13.20861406598522`,13.2177140659
8522`,13.22691406598522`,13.23601406598522`,13.24521406598522`,13.25431406598522`,13.
26351406598522`,13.27261406598522`,13.28181406598522`,13.29091406598522`,13.300114065
98522`,13.30921406598522`,13.31841406598522`,13.32761406598522`,13.33671406598522`,13
.34591406598522`,13.35501406598522`,13.36421406598522`,13.37331406598522`,13.38251406
598522`,13.39161406598522`,13.40081406598522`,13.40991406598522`,13.41911406598522`,1
3.42821406598522`,13.43741406598522`,13.44651406598522`,13.45571406598522`,13.4648140
6598522`,13.47401406598522`,13.48321406598522`,13.49231406598522`,13.50151406598522`,
13.51061406598522`,13.51981406598522`,13.52891406598522`,13.53811406598522`,13.547214
06598522`,13.55641406598522`,13.56551406598522`,13.57471406598522`,13.58381406598522`
,13.59301406598522`,13.60211406598522`,13.61131406598522`,13.62041406598522`,13.62961
406598522`,13.638814065985219`,13.64791406598522`,13.657114065985219`,13.666214065985
22`,13.675414065985219`,13.68451406598522`,13.693714065985219`,13.70281406598522`,13.
712014065985219`,13.721114065985219`,13.730314065985219`,13.739414065985219`,13.74861
4065985219`,13.757714065985219`,13.766914065985219`,13.776014065985219`,13.7852140659
85219`,13.794414065985219`,13.803514065985219`,13.812714065985219`,13.821814065985219
`,13.831014065985219`,13.840114065985219`,13.849314065985219`,13.858414065985219`,13.
867614065985219`,13.876714065985219`,13.885914065985219`,13.895014065985219`,13.90421
4065985219`,13.913314065985219`,13.922514065985219`,13.931614065985219`,13.9408140659
85219`,13.950014065985219`,13.959114065985219`,13.968314065985219`,13.977414065985219
`,13.986614065985219`,13.995714065985219`};

spectralData2states={-
21945.55374141546`,44252.74701247716`,23241.254174166148`,64399.97795382811`,-
13471.952142652564`,-48188.07478186286`,-43485.84235136511`,31256.652446494918`,-
17163.180656262168`,39640.13370627017`,-6903.677722879748`,-
54173.113206279355`,8428.781306004925`,-21307.268902991902`,50713.480400243374`,-
64034.66716933108`,5625.936531130965`,-55514.969770966316`,-
1811.2716897429664`,31750.06974790784`,31692.3415390364`,25009.474946517006`,43835.04
```

4863817966`,84667.73691933583`,-33552.70628616151`,985.4354676595756`,-
289.0850828343151`,33098.92198338454`,57278.2299476658`,21916.981099341403`,-
64162.798895831496`,7622.783561066932`,-17677.508730254638`,-
26614.897990955877`,17367.67887290254`,-31258.10223308519`,-
39132.48714399871`,31027.77141217275`,9623.764938662936`,-
48184.54508289945`,59360.8420927141`,-
2702.071553271667`,28105.8483974964`,33437.962841979424`,-381.5177457681459 7`,-
29694.46385753293`,-37283.60489551247`,-
21518.186457829255`,26741.734033556513`,8577.1834772123`,31582.420825139718`,17558.08
5279195577`,-7633.530428185629`,-357.2165272080771`,-34040.63225967375`,-
19108.12194331389`,-61636.55317550485`,-7981.080470171393`,-59437.78915497592`,-
74028.37946559135`,-65562.20836784094`,-35771.82714240925`,35948.84486891773`,-
34502.52583565932`,-31603.117257802314`,-33294.35310935366`,-270.5662260946038`,-
20531.79264773051`,-49065.83832646601`,-114118.40036207906`,-44977.72712806051`,-
4675.644323276078`,-6808.777863380883`,8671.95844698048`,-
8006.211691954464`,21520.85130479769`,-26870.372079383473`,31804.044608331424`,-
4981.286755975332`,7884.406763417822`,-6950.089959732049`,-
14196.440466897617`,22533.61921038625`,-55137.497451223455`,-
25987.244911394388`,70576.80024001267`,46828.91988197772`,9560.776864492373`,12607.69
3157777749`,38191.80423168779`,-8718.150618961194`,-
18485.755348292965`,9391.352062243053`,46249.202808804104`,49300.78932906358`,-
76959.09655622071`,5446.293816143884`,80285.47489188208`,24644.606060180344`,-
52870.78589127768`,19509.986330168227`,23707.636621601374`,11565.284046541467`,19578.
23922224481`,-3693.273217707979`,-3324.282649821243`,33434.49983767869`,-
44882.82691612653`,-75738.17306030686`,15511.511879751053`,-
28127.720246073746`,12481.251023262303`,-48448.92600249387`,-12594.522979654692`,-
72536.84656198857`,-19352.004697281405`,-15843.431647195266`,-
38914.56210944446`,28076.863875728774`,-
11224.537584251346`,36723.38478638258`,29456.30535669427`,68681.85383513411`,241.7590
7527797924`,36903.51232183146`,55584.840605868856`,-3538.946975158986`,-
62081.32576090085`,-31447.395526133754`,-34390.777469691915`,-3673.1756731248643`,-
9168.22779534919`,1485.6966578446645`,5845.547946677788`,-
19747.400311893784`,21654.844565369433`,22331.290568763678`,-67702.05594222745`,-
10967.88659666208`,-63817.85357429914`,11900.49935323103`,-6074.470108056875`,-
61912.86010391431`,-
40213.67045803754`,16697.254101824376`,60639.71002363225`,21182.066418352497`,-
49079.12542961744`,-45285.675756895886`,-18627.048674651793`,46585.49305156021`,-
26657.7351911968`,47431.704302156606`,-
36125.25557940954`,15591.586958443879`,8946.492170530591`,57718.98334731428`,25984.50
457131391`,34807.84890856926`,12353.86869280329`,16946.303462814805`,8489.51379743516
4`,7980.745509199964`,-19463.680126898416`,-22797.538323860223`,-
35935.95389106814`,5141.719795946631`,7659.208439557965`,15881.980343222996`,-
26017.738177034844`,26498.840249343702`,-77408.88022297862`,48706.07460891956`,-
50213.773494713074`,-7605.194118348973`,114394.52829344728`,87345.38674948843`,-
63158.378154535756`,43538.18410265078`,50754.74905405859`,51386.61109700247`,27027.21
994686097`,67317.44353739328`,46357.75709082712`,-29750.963851286117`,-
25812.730188730042`,11837.49049983255`,57713.37124309891`,-40840.2054783523`,-
47069.083798168846`,-7566.894810883675`,-13326.416908370098`,-
27213.823868389998`,10519.146490071524`,76.9098547869266`,48725.17728432083`,5174.987
8295251`,34817.922277542115`,11903.990293401044`,-10711.782233378037`,-
28579.549351132133`,23582.198938708276`,-62874.68666067623`,-5368.244287697699`,-
27193.205517201495`,69499.75786995557`,72460.66911200115`,39625.65658788932`,-
13926.22694695604`,-325.3770257196262`,-57736.09305404818`,-
59751.98392815022`,8.364533370320068`,-8609.214231114402`,45965.659820982204`,-
8732.90877823785`,-

75073.94852864547`,52648.22178356075`,11574.39313511502`,11706.582116360312`,-
65415.53828066211`,4242.468318066818`,-14678.350281321229`,-34673.517952505645`,-
42072.25796777215`,-64792.45884850611`,2864.2681241359746`,94308.58276785344`,-
3217.975790639793`,27395.174185023145`,-
2516.4985522240004`,20189.15871412109`,17869.276259508828`,62425.050447372705`,3321.8
98017977185`,38568.80341783802`,52128.885446781496`,25109.8198569100 8`,-
34048.04384750477`,3417.8314002137176`,-25808.41696698106 5`,40046.666754961276`,-
175.7160630192926`,47172.157618122`,-38802.04314522954`,-1661.7100537750914`,-
20618.011390832886`,-31410.820844360584`,-29028.105881954998`,13988.907051597003`,-
27007.603728460756`,-76669.68557868406`,-54317.51389505347`,-
67545.2130101234`,58033.32135076548`,46960.40274981944`,53782.33207627978`,32589.9872
3733435`,45744.79863145908`,-
22805.961537941563`,24095.045349021308`,14361.82300173217`,-
1232.7978139166032`,4171.139307917616`,12703.77195523838`,-37218.95056587444`,-
26226.49196984594`,-33125.43104989886`,-13700.730072362148`,-45606.98772810513`,-
53603.51975180816`,-30338.367307488257`,-
42202.19795849749`,43577.56745279426`,48682.05487590466`,-
9139.904667248737`,41646.39351345439`,39722.24163613641`,1587.2421302143778`,-
44607.902180237994`,-71042.88702141511`,-21722.801077634926`,-26594.634205707047`,-
42466.06123139215`,12676.441496331803`,-30411.21296782299`,-26745.345346610553`,-
2578.899647388155`,-16463.39240263488`,-55601.97297896685`,-4134.185685710994`,-
11700.122689904758`,43543.599805091864`,11666.090878686715`,67140.74885589328`,27832.
60537244249`,-48957.81812396267`,63891.02263546486`,-49592.94312437778`,-
20981.9008790097`,-41019.34699321218`,43273.75809268904`,49141.13039768567`,-
40068.86177673508`,20449.584891407656`,-9144.26694873899`,62785.56317581857`,-
11524.544189454435`,-27763.511121011492`,-9190.991192986798`,48724.94876933347`,-
3357.969464160252`,47008.24003019103`,-
12678.10796474051`,35894.4543517073`,31653.363849008376`,-
45105.18230536926`,50496.857870362524`,22987.47121472899`,1378.2839765351075`,21857.5
0111236116`,-16968.30472703693`,-46188.06086002895`,-
7379.903686780508`,41787.53514817451`,-35637.21922963846`,-21892.77383455169`,-
32768.623574932244`,19137.88045056157`,19899.957952969176`,54400.82746040909`,-
47346.85478657998`,6753.294550403757`,-64949.49869696344`,2666.2465822441027`,-
58235.603593307984`,-20926.543141498358`,11458.871320864942`,-23240.83857293257`,-
50738.863949355`,20760.173017207893`,-
55460.10394871166`,23833.558820340168`,22710.48690553599`,63534.91278615762`,22492.40
5477687247`,-46094.53480975106`,-102830.15256279876`,15446.823951603721`,-
34284.59884521215`,-5262.943260688483`,11430.81718413772`,-
21454.630511545405`,48187.58883283804`,9940.758011669386`,10244.44123171287`,52759.96
1182127554`,61569.73232700483`,42440.48076926352`,-9818.579020371339`,-
2438.297112707486`,15736.07903935124`,-55921.212259791995`,-
9773.80912525551`,5540.502280992032`,-61076.40069578861`,-61099.43375040534`,-
11508.35345025402`,7105.334515368995`,45160.41395545622`,-
14101.513567977272`,3049.5302035618665`,22747.26620432205`,-
21371.826321211738`,22012.199037372313`,13190.50576005493`,6482.347291198613`,71013.2
0822265463`,64110.59430301728`,92798.09514611469`,32061.417308122655`,43230.290488788
2`,38046.14864855338`,-8406.462737049083`,-26645.909030194438`,20049.943767645203`,-
76813.90924012312`,-
13493.631739044837`,67479.51253444793`,38290.95454011036`,69463.34395750321`,109867.5
0393996973`,113801.23798625905`,69687.52472571166`,114974.41218483858`,128600.8987843
6846`,110077.33876033867`,133604.0549238196`,107585.34333195885`,97153.09461919329`,1
55059.95949986944`,114095.06432637943`,197612.7098687619`,232280.382586823`,235652.05
978564033`,242153.60323459274`,202117.75691892914`,197544.25437480718`,188327.7262155
9954`,180573.4055507805`,210081.50305543948`,177535.28897925615`,228164.31104982`,296
041.6825362107`,266722.39506087615`,271161.69660896296`,248405.20061058836`,287999.52

32056895`,322454.45396234683`,250197.39950503228`,302156.45122634916`,217744.61581511
068`,252774.5263695416`,233723.65236828098`,230852.8670949265`,225290.0791008481`,206
817.9709562043`,213299.84801365723`,260999.13992668584`,143507.2722343721`,176483.592
63693713`,186477.76306418728`,188771.67411065823`,164732.7712116579`,75889.1301190675
1`,98693.1729968885`,155606.97566260508`,185169.71606307934`,207079.83174272164`,1120
97.11741037457`,136012.56207304454`,85240.6418288365`,64112.20924378572`,86787.266647
08334`,90004.72407299999`,22822.006827308407`,92364.66945916157`,35463.959960081615`,
81322.022730138`,53858.140527586715`,103267.67813017649`,121563.50432039783`,108120.0
489623907`,87830.73918746308`,39546.77956381471`,91002.06137838184`,78570.90499775585
`,83452.59583787402`,40493.07830160139`,2137.554120818832`,119984.29447367419`,-
33379.20430245068`,87868.94023419864`,33520.785523613675`,19067.737890321998`,57211.1
0294117013`,43683.06908179866`,57273.93795079926`,16218.172623975615`,-
35885.03630181473`,-1424.2957814121273`,50501.636740406575`,-
3917.722195921717`,17282.208694682216`,16961.81905950696`,39555.076220100535`,40373.5
2117760983`,15974.065729589502`,59714.91919099333`,19578.294142267085`,57085.17364585
7605`,-9303.259817858232`,-926.2516104706642`,76683.71626720046`,61705.63150348972`,-
32404.285267566112`,64018.80908554467`,11963.397718467751`,-
53980.34247049525`,64703.812964932855`,76917.66113406647`,38334.16721256309`,-
23607.461146287307`,-2381.220900208226`,-41559.14688269697`,-
27430.46340608064`,27365.4194602427`,34270.19709776479`,101407.07366578687`,9448.624
376444788`,15788.381357037037`,40947.82244352909`,1070.1917920140172`,91575.237743397
29`,62937.39470590806`,10670.353065575366`,444.09500254821717`,10174.195857132185`,69
158.54907662966`,-
39908.90001934765`,47396.87251369933`,28958.491660278527`,5061.129045201703`,78823.44
443165493`,45896.340342904674`,21071.260346696767`,28650.429849616798`,39749.11876215
387`,17631.360832348357`,99869.437633001`,159050.8859799533`,157367.4659119252`,11285
7.97212282261`,133005.38544847988`,150785.28134613181`,69672.03020908003`,80620.18520
516832`,71480.26541330581`,129162.17385476836`,168746.43033830484`,171626.34450118063
`,204502.44372808933`,198513.47251812692`,183161.561746704`,181044.44772810614`,11452
1.56919691947`,153368.33807314158`,161116.15815002407`,180963.3429174359`,175325.3055
196873`,183197.6553639054`,206524.59248652885`,275075.5511019314`,287960.76141925785`
,171502.97540318707`,226834.63153015968`,189438.63228222245`,194836.7328655961`,24300
3.51333071286`,232733.61952014337`,308748.5675904511`,292079.298460159`,198961.551747
79915`,269023.8486007158`,208564.10032855775`,232067.29851872576`,227288.13538531447`
,206853.81468117223`,123336.36037908767`,189003.51480750996`,151709.90949779635`,2017
65.84794850575`,140110.8026795348`,173595.8724318119`,161428.18763467035`,153188.2677
0643884`,117918.66639082346`,95427.01765969425`,165894.2012434695`,125964.94087516163
`,67355.87601239306`,26189.37912382689`,91383.60724410761`,93771.67324530105`,29750.3
0560349451`,40249.52951844533`,32747.084598781406`,42149.26220958643`,65830.732332514
77`,19975.140352159204`,-2628.498855993935`,84539.64810668697`,3550.1835678809393`,-
45529.717635602945`,23576.87151038164`,53815.61066688376`,72049.2338858177`,119845.07
876536033`,31595.758090011717`,23617.80653485114`,-29348.16950075717`,-
117142.45596491624`,8555.560686389817`,79263.4831297903`,37340.6168181982`,66322.6420
936932`,56530.53593671648`,48316.10304055574`,-
14354.16220312713`,35860.651060150725`,22833.76073094909`,26016.912047099777`,-
4273.336338646864`,28962.721642441207`,86564.72098390116`,4747.926183723482`,24979.32
9382327443`,33649.25629379209`,-19549.682973780524`,36586.543009427434`,-
37200.314560431994`,3309.5577768253183`,-24285.503987838263`,-113453.64028139287`,-
15711.73102315816`,868.259393891228`,42222.41498913202`,-
44766.977034875155`,23298.072191215277`,-7242.674549397953`,44485.66770672535`,-
10104.825416502226`,-7960.4224962417275`,4280.522044864008`,-
18891.41733657186`,12219.31245204256`,-
61612.28690745159`,19384.24009259816`,14199.178023254826`,54161.64828113475`,26377.89
8556756`,14854.639331193239`,27922.43089837922`,60336.80551881594`,-
20633.65059989475`,-31530.671339167853`,-

12762.05748198576`,23323.82534117426`,19699.27526078492`,-
51170.47990123664`,17760.780334365834`,-49559.95279787286`,4955.743495436128`,-
15314.49435583782`,13348.725011075949`,32879.75957384159`,-
16398.40505391924`,32427.74190967338`,29488.77830270359`,-1764.0650891183714`,-
20422.480207917368`,-75825.5712109438`,-86384.69903049683`,-2915.1311552562443`,-
17166.528864975175`,16660.466706683867`};

spectralData3states={42389.49233918438`,90847.22207634126`,105361.33099361022`,26658.
52443011644`,31323.800316299017`,134495.98239860844`,89684.31437595497`,46952.7199627
1164`,68835.45827304802`,90675.32773935571`,22916.395422605492`,-
44561.9381638652`,921.974610886136`,9735.423475126043`,-71169.91375052417`,-
35108.88368837579`,42008.22829019908`,21028.84829861142`,6058.217009115261`,-
32217.731195697852`,98429.58584895388`,-21498.526981513354`,-32159.652970199728`,-
17483.111736193714`,34625.8969568016`,-37262.227515251194`,4859.9810376655205`,-
11657.250897200902`,-54108.51414804244`,-11278.605561981563`,-57865.350013554744`,-
21710.54975276728`,-44741.42344680733`,77205.93435127562`,20340.563261570853`,-
23104.10892477915`,39904.31207907193`,84041.0488329236`,-
34466.31711028734`,13199.949820556647`,47517.037199480015`,-
83467.62907101608`,37940.017384735365`,36660.72238897927`,34988.922108971434`,15893.3
43843467901`,-80282.62789240223`,-30709.915603822745`,-
85971.31744503112`,20667.67349864473`,31617.370729517064`,-36358.12242537137`,-
119332.97531319942`,-11710.14541060086`,37299.60342424737`,3250.340256086399`,-
55597.718218409165`,-66615.46848883039`,40472.03042472928`,920.4278814511032`,-
25967.580924390873`,33361.794520048126`,28772.023512024465`,-
106678.09257576925`,22498.890904055377`,-70119.27081522786`,1565.0344579707398`,-
130149.06162066029`,-
34160.32898985883`,41520.981214571206`,22081.1173380109`,59149.79273790241`,28677.478
47278042`,-17593.253583092766`,-
56505.2426125643`,58432.06859610105`,35141.383729680856`,-
54155.51325866772`,7258.017270041002`,-64485.93533267328`,-47170.73907502299`,-
85950.07301111417`,-56629.37286053332`,-6422.591815101805`,-61165.05058430036`,-
5068.691022206162`,-74320.17315357052`,-116468.31704259083`,-80509.37921512898`,-
13432.232885492007`,-103325.59424640267`,-66130.58446555435`,-79311.78531766853`,-
58828.8819252334`,12732.443012176587`,-71586.31825022594`,-5577.368056761887`,-
47597.6014949003`,21988.942129841464`,17527.26665342863`,52206.639245345876`,-
32384.037245666055`,-17995.798125218378`,-
98511.65199991692`,90757.71208517949`,120427.08968438655`,-
14751.046770401981`,44241.12864715801`,-5893.546284830476`,-144881.24033489148`,-
75778.98580724037`,-
21064.019037001908`,38636.29187370378`,5156.243180171833`,39307.97082355048`,-
4679.101699336111`,98550.4029445961`,-5335.132373138419`,38687.4371453086`,-
64933.245116172686`,-19298.545530155043`,7439.977196976972`,74509.14636337929`,-
76855.09777443793`,-31692.350085751215`,-23463.35214764274`,-
13330.876815453077`,23681.011078077652`,-12948.924072578637`,5496.94682724516`,-
8137.807566345118`,-41202.099383176566`,-
46747.26117907575`,91632.07104355947`,12304.13935176097`,-64622.009778442545`,-
93746.45868858989`,19391.030207429227`,-34208.90107897647`,-
43032.229158578084`,8175.195600529439`,65080.20858192608`,139297.02595403473`,58831.7
1455070431`,83953.80709763787`,111564.37585509478`,113311.41699567526`,105728.5509543
2143`,220320.823590078`,315417.570160347`,384688.615703576`,577582.1184632723`,808773
.5239757772`,1.0453372369077714`*^6,1.569689158637727`*^6,2.167604336239395`*^6,2.771

7919837288545`*^6,2.8692970992254517`*^6,2.3839024609097824`*^6,1.6836296838604927`*^
6,1.3036986386268355`*^6,911273.7006248292`,594785.9226229502`,441335.56273448485`,31
4280.03845403163`,271959.9863157565`,81352.48467209621`,221226.73871967345`,157155.18
982441397`,59827.04123154562`,68766.07248860592`,112432.19536854513`,50937.4461156060
64`,21739.310765830356`,-
56275.894804643794`,145143.65015328684`,98020.36627245239`,17705.205694452732`,7502.8
19944119`,-
718.4826242271927`,49265.576203366894`,132610.41290735244`,35493.75999943349`,-
20122.331101865726`,116564.28945793366`,20951.84443248784`,53811.28426364525`,15443.8
55867825398`,7921.10459300239`,71100.96404349146`,-86514.4724863802`,-
63291.23903802396`,32642.547074024234`,71553.60158371262`,-71597.67935952471`,-
77665.37491313976`,-5780.662531555438`,-88369.1217548796`,131111.20174070683`,-
72696.10049102089`,-5459.240922901324`,-3749.670428561618`,99253.27012213955`,-
27254.950054945628`,-71949.86425611777`,-44421.98088283276`,-
127230.2310229449`,72968.5409326966`,63585.822827851705`,34528.852390695574`,30877.71
3927923967`,-1674.7449456491017`,-99129.23833426402`,13806.784441179067`,-
17127.842952171428`,617.8646815558955`,30359.884879587004`,65822.18349872595`,-
112200.65041135186`,-
21700.6141206315`,99325.4484111225`,97159.92431940457`,35483.42736182304`,-
10628.779420734058`,11867.186579855985`,93155.24633668849`,-
49768.7642166398`,42946.18628964769`,77559.99635317978`,40704.308108221805`,128368.26
682829564`,-
130764.17127416588`,19596.098273737567`,4592.215074265895`,62728.277729733345`,-
4548.679468063142`,20195.065021276794`,-
54731.30811538679`,30280.124172436965`,11645.310901570425`,86362.41981920412`,3703.54
90925418017`,58789.07345542899`,48887.856133456684`,-
21426.31050740186`,35418.23433959105`,-196.75508583341968`,-41470.22510911222`,-
63648.63551707547`,-8120.12526087008`,-12273.730605770928`,-
28987.675565561214`,3200.815179687369`,42180.94524028325`,-42434.21251187054`,-
26158.282056101063`,29896.19226526734`,-37951.31018724279`,27673.5148310733`,-
14783.37910129953`,-
10066.896898233546`,86906.61836428032`,3422.255574896899`,20566.070602369393`,14104.5
73443377745`,-82662.65296186975`,-17246.929044691173`,-68489.89871049643`,-
106508.22021945969`,-
34631.3166752409`,1197.713046218379`,68689.41954656695`,124254.04364376253`,-
15541.785512062566`,-
89859.572430516`,5461.520596791025`,10232.960544409085`,128331.20362041159`,105290.81
278995385`,-76805.69635584416`,-
10265.39999183198`,15043.174613021438`,110955.67526353057`,20468.79869333226`,-
35379.15896867642`,-49560.762447933026`,2082.8402594082245`,-
144292.0253555897`,9475.063099780209`,-24954.031783873877`,-14747.239388364449`,-
76253.41738318616`,59788.86981189252`,-80961.72925872885`,-
73089.09879717907`,7095.176663560373`,100067.36498053993`,24583.912338585487`,-
76490.05325142671`,-
30927.876677372882`,8333.265964840544`,131256.80268411108`,82782.23386052142`,-
50496.96765110273`,-4698.72039186065`,-67069.02613655699`,43885.1209806597`,-
7556.0762443739995`,61677.70078717918`,68508.45349825862`,48007.26339684081`,-
7384.261720062123`,-38617.094523832435`,-118002.07802512235`,-
112829.95137245192`,27087.272019041542`,-
22518.66068621099`,134330.2381437295`,34006.66286392882`,12457.24477669264`,-
66226.43619962495`,-63008.06476412346`,-

117942.8101240441`,67090.74592309575`,5698.736828361037`,-40568.62470358325`,-
63141.51323549658`,12650.65332269775`,-60142.21846163619`,-
36363.72850166204`,37177.4659057893`,-45813.04889358436`,12028.305356065439`,-
8117.72082538738`,8052.958025690624`,-16378.121615510037`,-
7852.643883007897`,23769.996488198376`,-3807.923551080247`,-48999.71277009884`,-
60014.81937097517`,-92039.62390848267`,-109244.79815141662`,-
17892.18464085915`,10874.82027547866`,68009.70043215677`,-
64240.74675735176`,60840.26547501816`,-
11348.535643298674`,80583.6120705864`,68783.35167526567`,-65649.86598496194`,-
11072.612796907135`,14036.631822766536`,19531.853532392855`,-
19413.09117830138`,48975.40221893905`,12440.99181757703`,-
86098.05572939519`,46009.92120591592`,22017.69158636367`,-
108751.67982727982`,20641.550263858546`,-118020.98701516556`,-108330.6495126807`,-
39551.68173009911`,-47428.14478478192`,-51138.721592401904`,85124.59633702949`,-
56675.810485941096`,62092.990213341516`,-32096.286509993246`,-69732.75968579031`,-
94062.12529919304`,-41447.998058537145`,-
32063.08570379976`,17888.567177597874`,113724.41302254767`,108320.46223237719`,-
5305.342756884166`,-
39948.08491574218`,4255.36840353265`,29348.324334323548`,73812.68203869679`,128072.57
777946879`,-
13233.12819590294`,10424.714534466431`,8788.50298898995`,76367.50159212908`,42947.615
43454079`,38330.506505425874`,38352.60593646962`,54925.558478816376`,46121.0499691612
35`,57498.29113208752`,29639.023249057107`,-
1319.1081701107005`,83900.7706594558`,376.14066060919237`,116520.95536008757`,50167.3
6460644944`,24771.557972523973`,256409.82962985573`,201797.43424581276`,233901.891583
85977`,356484.0548090657`,335100.215685745`,426324.84563741944`,558545.403033756`,721
786.7041385908`,686914.5544435304`,774221.186431438`,857686.7479551871`,688804.036724
3497`,604662.9319491547`,538574.3326991197`,391707.7062356684`,381757.77184583823`,30
2516.2590808986`,203805.78509724353`,98152.89751741149`,110641.15016979871`,139366.35
995549397`,209041.801264874`,176627.6596042572`,68327.29812226343`,24409.330749639514
`,63270.65081175098`,19477.71855374494`,70414.38336678936`,176002.32081988148`,28052.
557506057005`,32221.589819384943`,114188.79914267198`,49762.08345486869`,29552.785592
410783`,-
11358.601972528335`,16108.301171101259`,68349.97104041443`,82694.89759926764`,-
31066.230544264617`,25272.380627843726`,93949.35681608203`,17531.930520653317`,-
22604.835608510064`,8776.489205651198`,2464.8186142647937`,-
15138.684020410908`,116066.44483511573`,372.3240863366536`,14921.507388786493`,1097.6
14710841721`,21824.53054289237`,54870.03805555708`,165573.43051200494`,90854.81068012
665`,50588.03622366846`,-160371.67406461833`,-
29408.57334293431`,24242.872791282472`,5188.209074888033`,-
104391.28692066655`,33563.82381880813`,-
100723.79675296776`,25189.76367332364`,60660.87472092187`,-80665.62600674466`,-
13674.829414710079`,61945.57649564962`,11120.027575997796`,-137291.70918784072`,-
37437.6812544416`,-25636.15547492023`,-15251.559131491304`,-78128.87225041467`,-
35667.93281700867`,35701.01325740854`,9806.526648972522`,-34331.36704101055`,-
63373.38227102066`,49679.339436117996`,10307.350952218585`,-
45507.40440052686`,78038.42240285134`,16188.380720604702`,-
54109.21425196668`,39671.08008566238`,104582.1879795239`,38631.98324500299`,-
77480.8713057678`,-
14716.205905647363`,66482.56382428418`,48795.03181148593`,20392.048537786282`,-
26127.95907241608`,-25036.428952660288`,24913.80597753922`,-46542.00256993798`,-

44479.534670954985`,-9514.678016959493`,14264.73955168152`,47176.91329162153`,-
20369.47680076739`,-102210.42181399393`,-
73494.69424128457`,15555.992014816764`,22203.485829855847`,-
32108.77339376385`,45936.14839107231`,-85218.63254820462`,-78125.36597660398`,-
75868.17291274822`,-83242.76867186477`,23731.251383418858`,-128314.54126484414`,-
29189.258852528554`,3432.6995303076487`,33877.4308852055`,11593.576000601299`,10473.6
42425906486`,4381.84782200769`,-26851.377749283`,-44085.235463830526`,-
101631.01189041058`,3889.997126779085`,45653.8582654336`,-
54128.29606180678`,37466.488062982644`,-
87130.96712625101`,49249.234143016554`,76301.55376832132`,-
31075.27104207163`,58644.433075536865`,34961.65965117313`,156657.60019308765`,43974.7
97797604435`,59449.66793663616`,57851.97496715407`,141237.34530850983`,160999.1474018
4092`,37999.67583770142`,-
8767.175374853394`,123452.38284028228`,31225.27643522616`,108072.9761886078`,228630.8
5329812518`,94961.10058423155`,148316.0256797663`,317978.3434798983`,354587.389741688
9`,374784.5870118445`,479600.6075019686`,648656.002477467`,647719.0573483105`,849648.
8460270785`,736738.4722750651`,714475.8329698078`,640376.0949617836`,555273.123079267
3`,422791.37494775234`,348149.22043583676`,279169.58928943385`,214054.8238599256`,172
976.47408223615`,53265.65324883797`,92814.48412667563`,105501.37659672827`,29486.4519
82100054`,118052.71337583635`,64555.457016306354`,17410.460271907777`,-
2306.593084720084`,82055.89617938235`,93659.23536786454`,-44024.0811533397`,-
68251.5603121259`,-39647.12520764907`,-
95198.4292118013`,45740.85794815305`,47287.78172604521`,-40612.09946808307`,-
15628.3327924146`,-21065.01787758323`,31729.63998322666`,-
10810.366900599956`,69024.42293768667`,30308.440663984005`,34750.64286955298`,-
48821.5007790402`,-33634.51063327765`,38953.71998252559`,-61845.75288002251`,-
5181.156387674922`,120068.15946677112`,57231.552733937344`,2206.2789744911447`,121760
.15705670044`,66140.56891002628`,18924.818646123942`,2940.8144535023393`,-
4482.549321637529`,40499.258922004236`,-30480.146303563593`,7939.217884775675`,-
8663.540649352979`,-17315.23989533913`,-49348.37188741465`,-13892.862021000536`,-
14182.466670474247`,5302.991779377923`,-60506.94550234212`,-31145.50668838888`,-
40823.90061768704`,71099.47763295767`,23426.307477636175`,64613.113641905795`,61878.0
6202923555`,1521.9366103725883`,27010.79866221541`,76028.80630628162`,25592.489494453
86`,60987.45519348206`,89923.3754935538`,-23629.17751400852`,13036.303637926107`,-
28634.83218422893`,-48618.06332040721`,-128169.28525219887`,-163146.86757893098`,-
510.8772872676102`,-62608.82360124564`,-80189.39829391554`,-25063.646330421463`,-
47172.71179306704`,-15208.285290402388`,-11012.874402408586`,-
10414.228351616608`,21303.748646230986`,-
100584.90883773797`,37099.336103944835`,7166.074701253711`,-
65378.68916913682`,75121.82284599668`,69620.63095018403`,47200.98296935321`,-
6985.178929597801`,-132316.72245013624`,25857.621983034125`,-45137.208314831434`,-
66268.40458762759`,10263.012896857732`,24376.023555430897`,-
33261.95564446047`,10366.836413784511`,93517.47173214292`,53963.150525350226`};

Slide 2

Two-state lineshape fitting

spectrum at 1.57 guest equiv.
δA1 and δA2 are obtained independently from peak maxima at lower No. of equivalents (see next slide)
pA1 = pA2 = 1/2

```
fixParRule={RA1->30,RA2->30,δA1->13.079,δA2->11.759};
fitRule=FindFit[Transpose@{ppmData,spectralData2states},{ twoStateExchFunc[δ,{δA1,δA2
}/.fixParRule,{RA1,RA2}/.fixParRule,{kA,kA}/.fixParRule,M] ,M>0,kA>0},{{kA,50},{M,5
10^8}},δ]
allParRule=fixParRule~Join~fitRule;
plotData=Transpose@{ppmData,spectralData2states};
plotDataFit=Transpose@{ppmData,twoStateExchFunc[ppmData,{δA1,δA2}/.allParRule,{RA1,RA
2}/.allParRule,{kA,kA}/.allParRule,M/.allParRule]};
ListLinePlot[{plotData,plotDataFit},ScalingFunctions->{"Reverse",Identity},PlotRange-
>{{8,14},Full},ImageSize->450,AspectRatio->1/1.618,Axes->False,
 Frame->{{False,False},{True,False}},BaseStyle->{FontSize->24},FrameLabel-
>{Style["ppm"]},PlotStyle->{Black,Red}]
```

{kA->443.46,M->2.31207*10^{8}}

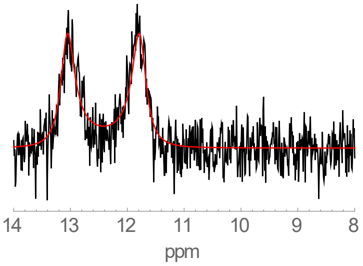


```
twoStateExchFunc[δ_,δ0List_,R2List_,kList_,M0_]:=Module[{αA,αB,δA,δB,δToΩConvFac,Ω,ΩA
,ΩB,RA,RB,kAB,kBA,pA,pB,P,Q},δToΩConvFac=2π*500.13;
   {δA,δB}=δ0List;
   Ω=δ*δToΩConvFac;
   ΩA=δA*δToΩConvFac;
   ΩB=δB*δToΩConvFac;
   {kAB,kBA}=kList;
   {RA,RB}=R2List;
   {pA,pB}={kBA/(kAB+kBA),kAB/(kAB+kBA)};
   αA=RA+I (Ω-ΩA);αB=RB+I (Ω-ΩB);
   P=pA αB+pB αA+kAB+kBA;
   Q=αA αB+kAB αB+kBA αA;
   M0 Re[P/Q]];
```

Slide 3

Three-state lineshape fitting

spectrum at 0.47 guest equiv.
δA1, δA2 and δC are obtained independently from peak maxima
pA, pC are obtained independently from peak integrals (2 pA + pC = 1)

```
fixParRule={RA1->30,RA2->30,RC->30,δA1->13.079,δA2->11.759,δC->9.433,pA->0.243,pC->0.512};
fitRule=FindFit[Transpose@{ppmData,spectralData3states},{ threeStateExchFunc[δ,{δA1,δA2,δC}/.fixParRule,{RA1,RA2,RC}/.fixParRule,{kA,kA,kAC,kAC pA/pC,kAC,kAC pA/pC}/.fixParRule,M] ,M>0,kA>0,kAC>0},{{kA,50},{kAC,100},{M,5 10^8}},δ]
allParRule=fixParRule~Join~fitRule;
plotData=Transpose@{ppmData,spectralData3states};
plotDataFit=Transpose@{ppmData,threeStateExchFunc[ppmData,{δA1,δA2,δC}/.allParRule,{RA1,RA2,RC}/.allParRule,{kA,kA,kAC,kAC pA/pC,kAC,kAC pA/pC}/.allParRule,M/.allParRule]};
ListLinePlot[{plotData,plotDataFit},ScalingFunctions->{"Reverse",Identity},PlotRange->{{8,14},Full},ImageSize->450,AspectRatio->1/1.618,Axes->False,
 Frame->{{False,False},{True,False}},BaseStyle->{FontSize->24},FrameLabel->{Style["ppm"]},PlotStyle->{Black,Red}]
```

{kA->55.483,kAC->50.3021,M->4.46579*10^8}

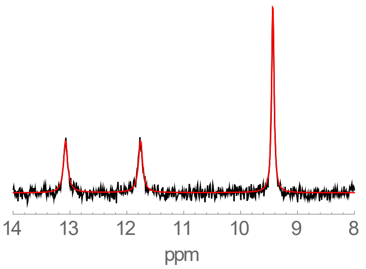


```
threeStateExchFunc[δ_,δ0List_,R2List_,kList_,M0_]:=Module[{αA,αB,αC,δA,δB,δC,δToΩConvFac,Ω,ΩA,ΩB,ΩC,RA,RB,RC,kAB,kBA,kAC,kCA,kBC,kCB,pA,pB,pC,πA,πB,πC,P,Q},δToΩConvFac=2π*500.13;
   {δA,δB,δC}=δ0List;
   Ω=δ*δToΩConvFac;
   ΩA=δA*δToΩConvFac;
   ΩB=δB*δToΩConvFac;
   ΩC=δC*δToΩConvFac;
   {kAB,kBA,kAC,kCA,kBC,kCB}=kList;
   πA=kBA kCA+kBC kCA+kBA kCB;
   πB=kAB kCA+kAB kCB+kAC kCB;
   πC=kAC kBA+kAB kBC+kAC kBC;
   {RA,RB,RC}=R2List;
   {pA,pB,pC}={πA,πB,πC}/(πA+πB+πC);
   αA=RA+I (Ω-ΩA);αB=RB+I (Ω-ΩB);αC=RC+I (Ω-ΩC);
   P=pA (αB αC+αB (kCA+kCB+kAC)+αC (kBA+kBC+kAB))+pB (αA αC+αA (kCA+kCB+kBC)+αC (kAB+kAC+kBA))+pC (αA αB+αA (kBA+kBC+kCB)+αB (kAB+kAC+kCA))+(πA+πB+πC);
   Q=αA αB αC+αA αB (kCA+kCB)+αA αC (kBA+kBC)+αB αC (kAB+kAC)+αA πA+αB πB+αC πC;
   M0 Re[P/Q]];
```
